# Antifungal activity and biocompatibility assessment with molecular docking and dynamic simulations of new pyrazole derivatives

**DOI:** 10.1186/s12896-025-00948-8

**Published:** 2025-02-06

**Authors:** Basma T. Abd-Elhalim, Ghada G. El-Bana, Ahmed F. El-Sayed, Ghada E. Abdel-Ghani

**Affiliations:** 1 Department of Agricultural Microbiology, Faculty of Agriculture, Ain Shams University, PO Box 68, Hadayek Shoubra, Shubra El-Khaimah, Cairo, 11241 Egypt; 2 Department of Chemistry, Faculty of Science, Mansoura University, El-Gomhoria Street, Mansoura, ET-35516 Egypt; 3 Mansoura University Student’s Hospital, Mansoura University, El-Gomhoria Street, Mansoura, ET-35516 Egypt; 4 Microbial Genetics Department, Biotechnology Research Institute, National Research Centre, Giza, Dokki, 12622 Egypt; 5 Egypt Center for Research and Regenerative Medicine (ECRRM), Cairo, Egypt

**Keywords:** Antifungal activity, Biocompatibility activity, Dynamic simulations, Molecular docking, Pyrazole derivatives

## Abstract

**Background:**

Because of their many bioactivities, which include psychoanalytic, antifungal, antihypertensive, anti-inflammatory, and antiviral properties, pyrazoles and their derivatives are attracting interest in pharmacology and medicine, the pressing need for novel fungicides is increased for lessened by the growing microbiological resistance of illnesses to recognized antibiotics.

**Objective:**

The current work validates the results and pyrazole binding sites as potent antifungals by investigating many pyrazole derivatives as antifungal agents. The biocompatibility was assessed using an HFB4 normal human skin cell line.

**Methods:**

The biocompatibility was evaluated using an HFB4 normal human skin cell line and the findings of pyrazole binding sites were confirmed using molecular docking. The antifungal investigation was against 4 fungal pathogens: *Aspergillus flavus* ATCC 9643, *A. niger* ATCC 11414, *Rhizopus oryzae* ATCC 96382, and *Penicillium chrysogenum* ATCC 10106.

**Results:**

Among 20 different Pyrazole derivatives, Pyrazole **3b** is the most effective compound against *A. niger* ATCC 11414 and *A. flavus* ATCC 9643 with IZDs and AIs of 32.0 mm (1.10) and 30.0 mm (1.0), respectively. Followed by compound **10b** scored 28 and 20 mm for *A. niger* and *P. chrysogenum* ATCC 10106, respectively. While *R. oryzae* ATCC 96382 exhibited resistance with all pyrazole compounds. The study found that pyrazole **3b** showed 100% antifungal activity between 1000 and 500 μg/ml, 50% at doses of 250 μg/ml, and no antifungal action at a dose of 125 μg/ml against the studied pathogenic fungal strains. The biocompatibility investigation showed that the **3b** compound was completely safe with no IC_50_ dose obtained. The effectiveness of several pyrazole compounds against fungal targets was confirmed through molecular docking studies. The results highlighted that compounds **3b, 3g, 3h, 10b, 7**, and **12** displayed strong binding energies, effectively engaging with the active sites of key proteins in various fungi such as FDC1 in *A. niger*, uridine diphosphate *N*-acetylglucosamine (UDP-GlcNAc) in *A. flavus*, and Adenosine 5′-phosphosulfate kinase in *P. chrysogenum*. These interactions encompassed diverse molecular bonding types, suggesting these compounds’ potential to hinder enzyme activity and demonstrate notable antifungal properties. Additionally, the computational ADMET “Absorption–distribution–metabolism–excretion–toxicity” analysis of these compounds revealed adherence to Lipinski’s rules, indicating favorable physicochemical characteristics. The molecular dynamic simulations of Adenosine 5’-phosphosulfate kinase in *P. chrysogenum*, UDP-N-acetylglucosamine in *A. flavus*, and FDC1 in *A. niger* with **10b** also demonstrated the formation of stable complexes with favorable values of Root Mean Square Deviation (RMSD), Root Mean Square Fluctuation (RMSF), Solvent Accessible Surface Area (SASA), and Radius of Gyration (Rg). These findings support the compounds’ potential in ongoing therapeutic development projects.

**Conclusion:**

The study found that pyrazole **3b** was the most effective antifungal agent. The compounds’ strong binding energies with fungi proteins suggest potential drug development.

**Supplementary information:**

The online version contains supplementary material available at 10.1186/s12896-025-00948-8.

## Introduction

**Fig. 1:**
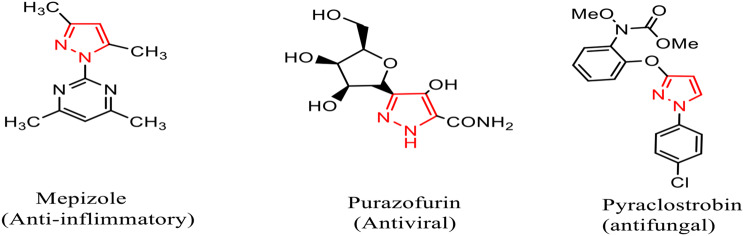
Bioactivities of pyrazole derivatives as drugs and pyrazole-containing natural products

Several recent studies have focused on the production and biological activities of many novel antifungal compounds. The pressing need for novel fungicides is made easier by infections’ growing microbial resistance to recognized antibiotics [1]. One of these compounds are pyrazoles and their derivatives which gain attention in the fields of pharmacology and medicine due to their many bioactivities, which include psychoanalytic, antifungal, antihypertensive, anti-inflammatory, ulcerogenic, cytotoxic, muscle-relaxant, antibacterial, antioxidant, tranquilizing, hypnotic, antidepressant, and analgesic activities [2–13] (Fig. 1). In Agri chemistry, they could be applied as fungicidal [11], insecticidal [12], and herbicidal [13–15], and acaricidal agents [16]. Pyrazoles are associated with 3 carbon atoms and 2 nitrogen atoms arranged in close proximity make up the five-membered ring structure of heterocyclic organic compounds known as pyrazoles [1, 3, 7, 17]. This category includes compounds such as isoxazole, oxazole, pyrazole, oxadiazole, imidazole, triazole, tetrazole, and thiazole [2–5]. A few dermatophytes (fungus that cause ringworm) and *Aspergillus* sp. are examples of resistant fungi. A more recent species, *Candida auris*, is very resistant to antifungal medications and may spread rapidly in medical environments [7, 17]. A series of pyrazole compounds have been developed and marketed as fungicides, including furametpyr, bixafen, penflufen, penthiopyrad, and isopyrazam which have the ability to inhibit succinate dehydrogenase [17]. Furthermore, a number of isoxazole compounds, such as sulfamethoxazole and oxacillin, have been created as medications and insecticides due to their antiviral, antifungal, insecticidal, and herbicidal properties. The extensive usage of isoxazole derivatives in pesticide chemistry and medicine has drawn a lot of attention [18]. Significant fungicidal activity were demonstrated by a variety of cycloadducts–pyrazoles against the necrotrophic plant disease *Corynespora cassiicola* [16, 17]. *Rhizoctonia solani* was well inhibited by isoxazolol pyrazole carboxylate [17]. The new pyrazole-thiazole carboxamides outperformed the conventional fungicide thifluzamide *in vitro* against *R. cerealis* [17, 18]. The current study examines several pyrazole derivatives as antifungal agents confirming the findings and pyrazole binding sites as effective antifungals. The most active pyrazole compound will be selected according to its high bioactivity as an antifungal agent according to estimating the clear zone of inhibition and activity index. It also evaluates the biocompatibility of the compounds using an HFB4 normal human skin cell line to be applied in future investigations as an effective antifungal agent in the pharmaceutical or agriculture sector.

## Materials and methods

### Chemicals and media sources

Supplier of malt agar (CM0059) was OXOID in the UK. The Amoun Pharmaceutical Company in Cairo, Egypt provided the standard antibiotic Fluconazole (1000 µg/ml). Every chemical is of analytical grade.

### Fungal strains source

This study investigated strains of four distinct fungal pathogens: *Aspergillus flavus* ATCC 9643, *A. niger* ATCC 11414, *Rhizopus oryzae* ATCC 96382, and *Penicillium chrysogenum* ATCC 10106. Every strain was acquired from the Dep. of Micro. Fac. Agric., Ain Shams Uni., in Egypt. Initially kept and preserved in malt agar medium at 4 °C, the pathogens were then grown overnight at 28 °C in malt broth medium to evaluate the antifungal activity.

### Fungal standard inoculum preparation

Fungal pathogen standard inoculum was prepared by following the procedure described by [19, 20]. To assess the antifungal activity, the pathogens were first maintained and stored at 4 °C in malt agar medium. They were then cultured in malt broth medium for a whole night at 28 °C.

### Preparation of pyrazole derivatives

**Scheme 1:**
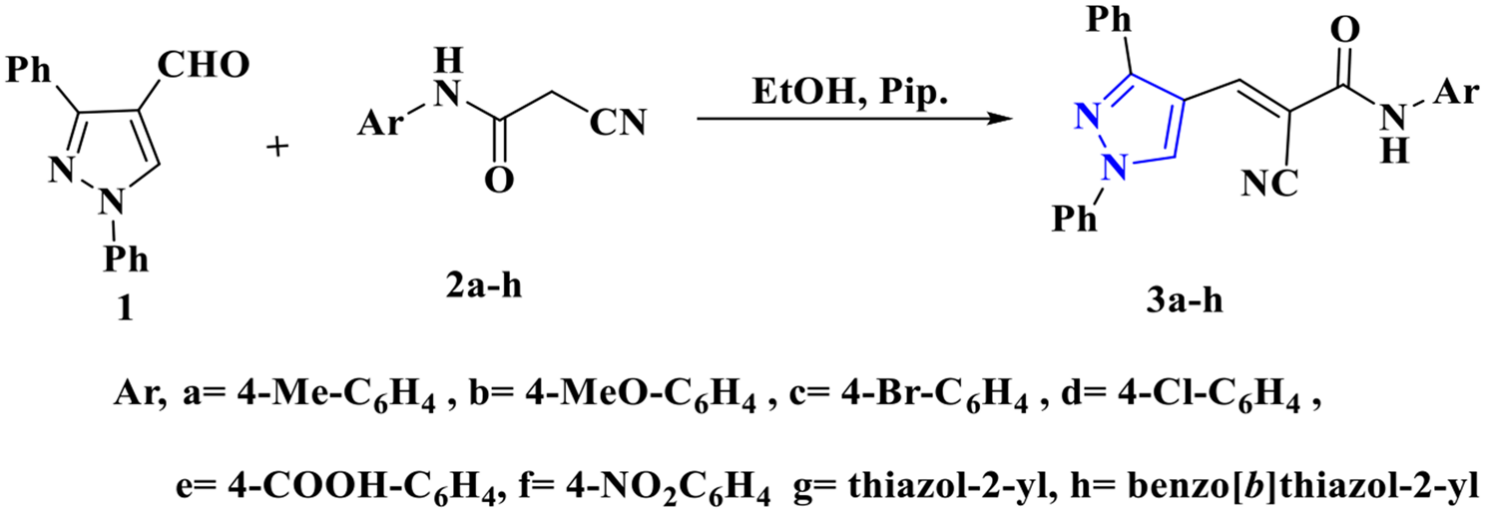
Synthesis of acrylamide derivatives **3a-h**

From the previous work of [21], 1,3-diphenyl-1*H*-pyrazole-4-carbaldehyde (**1**), which was utilized as precursor for the preparation of new pyrazole derivatives and investigated for their antifungal activity. The Structure of synthesized compounds were identified and confirmed using spectral analyses as IR, MS, NMR analysis. First, 2-cyano-3-(1,3-diphenyl-1*H*-pyrazol-4-yl)-*N*-Substituted-acrylamide derivatives **3a-h** was synthesized *via* condensation of 1,3-diphenyl-1*H*-pyrazole-4-carbaldehyde (**1**) with *N*-substituted cyanoacetamide derivatives **2a-h** in EtOH/Pip. (Scheme 1).

The existence of the characteristic bands at *v* = 1687–1614, 2217–2204, and 3363–3326 cm^−1^ for CO, CN, and NH, respectively, allowed the acrylamide derivatives **3a–h** to be identified by their structures in their IR spectra. Additionally, singlet signals belonging to NH_hydrazone_, CH_pyrazole_, and =CH protons were detected in the ^1^H NMR spectra of compounds **3a–h** at *δ* ranging from 10.19 to 13.24, 9.21 to 9.25, and 8.14 to 8.98 ppm, respectively. Furthermore, the ^13^C NMR spectra of **3a-g** showed signals at (*δ*) between 155.26–161.58, 155.02–155.49, and 115.25–117.07 ppm which were ascribed to C=O, =CH, and CN, respectively.

**Scheme 2:**
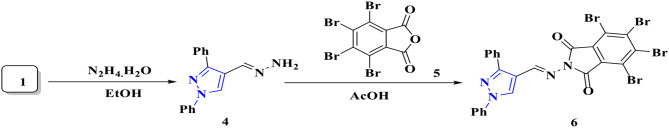
Synthesis of 4,5,6,7-tetrabromo-2-(((1,3-diphenyl-1*H*-pyrazol-4-yl)methylene) amino)isoindoline-1,3-dione (**6**)

Additionally, hydrazineyl-pyrazole **4** was prepared from refluxing compound **1** with hydrazine hydrate (N_2_H_4_.H_2_O) in EtOH. Compound **4** reacted with 4,5,6,7 tetrabromoisobenzofuran-1,3-dione (**5**) in AcOH to give 4,5,6,7-tetrabromo-2-(1,3-diphenyl-1*H*-pyrazol-4-yl)methylene)amino) isoindoline-1,3-dione (**6**) (Scheme 2). Compound **6’**s IR revealed a new peak for the 2 (C=O) groups at 1723 and 1754 cm^−1^. Additionally, compound **6**’s mass spectrum revealed a molecular ion peak at m/z = 708 that matches their suggested structure and the molecular formula C_24_H_12_Br_4_N_4_O_2_.

**Scheme 3:**
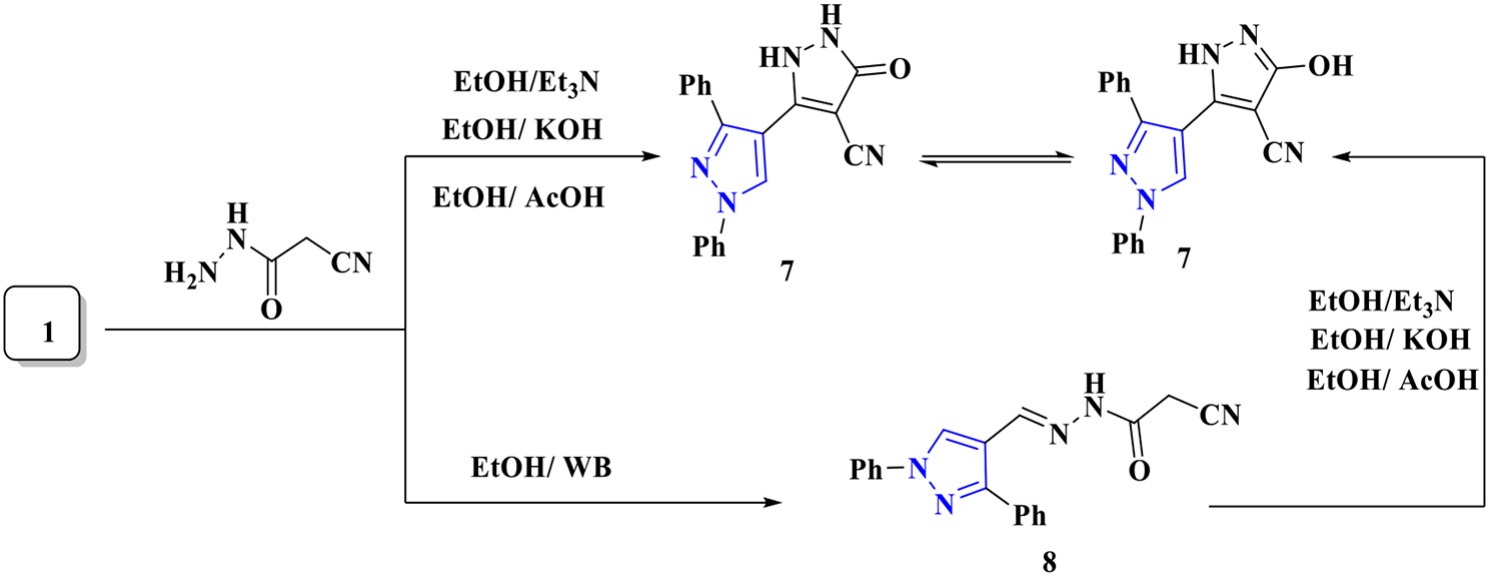
Preparation of pyrazole derivatives **7** and **8**

Moreover, pyrazolinone derivative **7** was synthesized from reaction of cyanoacetohydrazide with pyrazole **1** under basic and/ or acidic conditions in Scheme 3. While pyrazoloacetohydrazide **8** is formed as an *N*-condensation product by performing the same reaction in the absence of a base in a water bath as shown in (Scheme 3).

Compound **7**’s ^1^H NMR spectra showed multiple signals for aromatic protons at *δ* = 7.34–8.23 ppm, in addition to two singlet signals for NH and CH_pyrazol_ protons at *δ* = 8.58, 9.28 ppm. On the other hand, a mixture of syn- and anti-isomer in a 25% and 75% ratio, respectively, was identified by the ^1^H NMR analysis of **8**. Two singlet signals for CH_2_ protons and two singlet signals for the =CH proton were detected in the ^1^H NMR analysis of **8** at *δ* = 4.13, 8.13 ppm for the anti-isomer and *δ* = 3.79, 8.25 ppm for the syn-isomer, respectively.

**Scheme 4:**
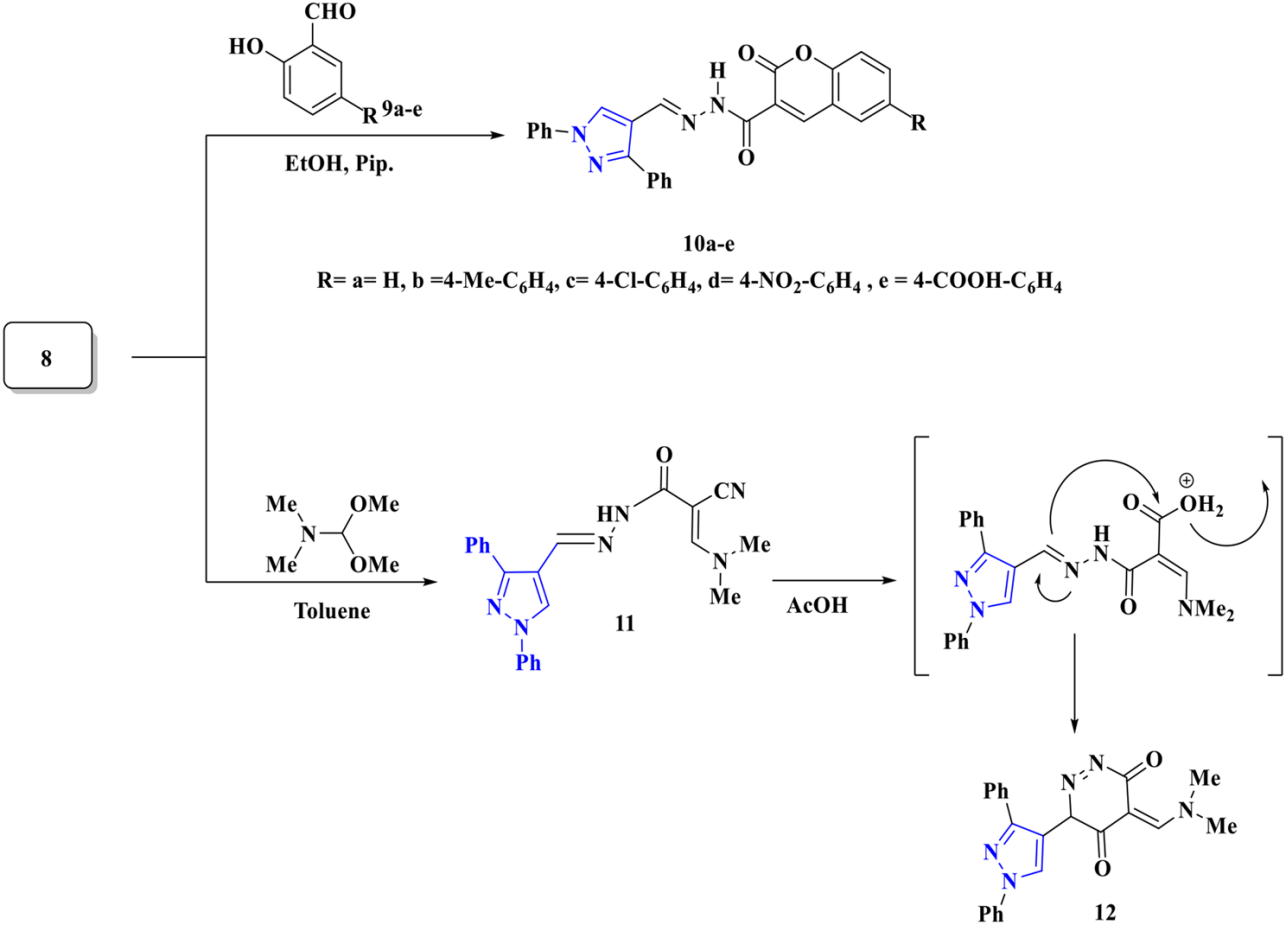
Formation of chromene-3-carbohydrazide derivatives **10a-e** and pyridazine derivative **12**

Furthermore, condensation of acetohydrazide **8** with substituted salicylaldehyde **9a–e** at 50 °C under stirring condition yielded pyrazolo carbohydrazide chromene derivatives **10a–e** (Scheme 4). The absorption bands at 1675–1682 cm^−1^ were visible in the IR spectra of **9a–e**, suggesting the existence of C=O groups. Additionally, ^1^H NMR revealed peaks for the NH protones at *δ* ranging from 11.72–13.46 ppm, which validated the structure for **9–e**.

Finally, refluxing acetohydrazide **8** with DMF-DMA afforded acrylohydrazide **11** in excellent yield. Moreover, pyrazolo pyridazine **12** was produced by acidic hydrolysis of the cyano group and cyclization reaction of compound **11** under acidic medium (AcOH) (Scheme 4). Compound **11**’s ^1^H NMR spectra showed two singlet signals at 3.23 and 3.30 ppm, which correspond to two Methyl groups, which appeared at 47.57 ppm in its ^13^C NMR spectrum. Additionally, compounds **11** and **12**’s mass spectra showed molecular ion peaks that corresponded to their molecular formulae, supporting their suggested structures.

## Antifungal influence of pyrazole derivatives

According to [22], the well-diffusion technique was used to test the antifungal activity. This included using a 9.0 mm cork borer that had been sterilized to create wells in the malt agar layer. Sterile malt agar was poured into each sterile petri plate after 50 μl of the standard spore suspension of each fungus (10^8^ Spores/ml) had been planted and the medium had been equally distributed across the petri dish. After adding 1000 µg/ml of control pyrazole compounds and the standard antifungal (Fluconazole 1000 µg/ml) to each well, the petri dishes were incubated at 28 °C for 72 h. The diameter of the standard reference antifungal was compared to the millimeters measurements of the inhibitory zones. The formula according to [22] was utilized to compute the activity index (AI).

1 $$AI = {\text{ }}\frac{{Inhibition{\text{ }}zone{\text{ }}diameter{\text{ }}of{\text{ }}the{\text{ }}tested{\text{ }}pyrazole}}{{Inhibition{\text{ }}zone{\text{ }}diameter{\text{ }}of{\text{ }}the{\text{ }}standard{\text{ }}antifungal{\text{ }}agent}}$$

## Evaluation of pyrazoles minimal fungal inhibitory concentration (MFIC)

MFIC, or minimal fungal inhibitory concentration, is the lowest amount of natural or synthetic antifungal medication that stops the fungus from growing significantly. In diagnostic laboratories, minimum inhibitory concentrations (MICs) are essential for confirming fungal resistance to an antifungal drug and monitoring the effectiveness of new antifungal medicines. MICs were computed in accordance with the recommendations established by the National Committee for Clinical Laboratory Standards (CLS) [19, 22]. In dimethyl sulfoxide (DMSO), pyrazoles were serially diluted twice (1/2, 1/4, and 1/8) and heated at 70 °C for 10 min to get final concentrations of 1000–125 μg/ml, respectively. After that, these dilutions were put into wells that had already been created on plates with fungal infestations. Using a calibrated micropipette, fungus spore suspensions were prepared and added to malt agar welled plates then incubated for 72 h at 28 °C.

### Pyrazoles minimal lethal fungal concentration (MLFC)

The term “minimal lethal fungal concentration” (MLFC) refers to the lowest concentration of an antifungal drug that may prevent the visible growth of fungi. The fungus was initially subjected to different concentrations of the pyrazole compounds in order to ascertain this concentration. After that, the growth from the MIFC stage was moved to regular malt agar plates. After that, these plates are incubated for 72 h at 28 °C. After then, any growth—or lack thereof—is noted and studied [19, 22].

### Pyrazole derivatives effect

Following the acquisition of the MFIC and MLFC records, the ratio was computed. The fungicidal activity of a pyrazole compound is defined as a ratio higher than or equal to 4. However, ratios of two or less suggest that activity is at a standstill [19–22].

2 $$Pyrazoles{\text{ }}derivatives{\text{ }}effect = \frac{{Pyrazole{\text{ }}minimum{\text{ }}lethal{\text{ }}fungal{\text{ }}centration{\text{ }}\left( {MLFC} \right)}}{{Pyrazole{\text{ }}minimum{\text{ }}fungal{\text{ }}inhibition{\text{ }}concentration{\text{ }}\left( {MIFC} \right)}}$$

### Molecular docking of pyrazole-synthesized compounds

Receptors for proteins derived from RCSB PyMOL software was used to enhance the structure of Table 1. This required getting rid of ions, water molecules, and preexisting substances. Using BIOVIA drawing, the compounds’ chemical structures were shown. Open Babel was then used to convert each chemical into the mol2 format [23]. For docking, the molecules were then converted into the pdbqt format using AutoDock tools. AutoDock Vina was used to build ligand-centered maps before the actual docking procedure [24]. All information related to grid boxes centers and sizes for the three protein were mentioned in Table 1. Finally, the 2-D interactions between the target proteins and the ligands were analyzed using the Discovery Studio program.

**Table 1:** List of fungal strains, PDB IDs, active sit coordinates center and sizes, and reference compounds

Organism	Targets	PDB ID	Active sit coordinates Centre and sizes			Binding site residues	Native Ligand Id	Reference	RMSD value
			X-center X-size	Y -Center Y-Size	Z -Center Z-Size				
*A. niger*	The FDC1 protein	4ZA5 (1.10 Å)	19.96 (25.0)	5.08 (25.0)	20.12 (25.0)	Ile171 Gln190 Ser223	4LU	Fluconazole	0.240
*A. flavus*	UDP-N-acetylglucosamine	6G9V (1.75 Å)	27.52 (25.0)	63.35 (25.0)	−2.29 (25.0)	Tyr330 Glu407 Glu329	UD1	Fluconazole	0.845
*P. chrysogenum*	Adenosine 5’-phosphosulfate kinase	1M7G (1.43 Å)	72.07 (25.0)	36.33 (25.0)	11.54 (25.0)	Gly37 Lys38 Ile106	AV2	Fluconazole	0.684

### In silico ADMET prediction

The ADMET lab 2.0 server was used to calculate the compound’s physicochemical characteristics and Absorption, Distribution, Metabolism, Excretion and Toxicity (ADMET) [25].

### Molecular dynamics (MD) simulation

To further validate the logic and dependability of the docking results, MD simulations were run for 50 ns as simulation time with the GROMACS 2018 program. CHARMM36 force field parameters were used to design the protein’s topology. The compound topology was also built using the Geoff server. Ligands were subjected to position constraints following coordination. NVT and NPT equilibrium tests were carried out for 1000 ps at 300 K and 1.0 bar. Following the MD simulations, the radius of gyration (Rg), root mean square deviation (RMSD), and root mean square fluctuation (RMSF) were calculated [26].

### Biocompatibility of the most active pyrazole compound

The biocompatibility of the most active pyrazole compound was evaluated using HFB4 normal human skin cell line. A complete monolayer sheet was created by incubating the 96-well tissue culture plate at 37 °C for 24 h following inoculation with 1 × 10^5^ cells/ml (100 µl/well), following the procedure described by [27]. Following the establishment of a confluent sheet of cells, the growth material was collected from the 96-well micro titer plates, and the cell monolayer underwent two washing media washes. In RPMI medium with 2% serum (maintenance medium), the most active pyrazole compound was divided into twofold dilutions. They then evaluated 0.1 ml of each dilution in several wells. We looked at the cells for physical signs of toxicity, including granulation, rounding, shrinkage, and partial or complete loss of the monolayer. Following the phosphate buffer solution’s preparation, each well received 20 µl of a 5 mg/ml MTT (3-(4,5-dimethylthiazol-2-yl-)-2,5 diphenyltetrazolium bromide) solution from Bio Basic Canada Inc. After that, place it on a shaking table and shake it for five minutes at 150 rpm to completely integrate the MTT. to allow MTT to metabolize, followed by incubation for 4 h at 37 °C with 5% CO_2_. Media was taken out if necessary, and paper towels were used to wipe out any remaining material from the plate. One may re-dissolve formazan, a metabolic product of MTT, in 200 µL of DMSO. Put on a shaking table and shaken for five minutes at 150 rpm to fully mix the formazan and solvent. At 560 nm, optical density was measured, and at 620 nm, background was subtracted. There should be a direct relationship between optical density and cell number.

3 $\% {\text{ }}Cell{\text{ }}viability = \frac{{Mean{\text{ }}Abs{\text{ }}control - Mean{\text{ }}Abs{\text{ }}of{\text{ }}Pyrazol{\text{ }}}}{{Mean{\text{ }}Abs{\text{ }}control}}{\text{ }} \times {\text{ }}100$

Abs absorbance at 560 nm.

### Statistical analysis

All collected data were analyzed using IBM® SPSS® Statistics software (2017), and a Duncan test with a P-value of 0.05 was performed in compliance with [28].

## Results

### Pyrazole derivatives antifungal activity

After evaluating the antifungal efficiency of pyrazole derivatives against dangerous fungi, Table 2 showed that, out of the twenty pyrazole compounds, compound **3b** was the most effective. The inhibition zone diameters (IZD) on well-agar diffusion plates ranged from 32.0 mm to 11.0 mm. The IZDs of the Fluconazole control antibiotic ranged from 32.0 mm to 20.99 mm. *A. niger* and *A. flavus* had the highest IZDs and AIs of all the investigated fungi, measuring 32.0 mm (1.10) and 30.0 mm (1.0), respectively, in the case of the **3b** compound which investigated as the most effective pyrazole compound. In the second rank came compound **10b** which scored IZDs of 28 and 20 mm in the case of *A. niger* and *P. chrysogenum* with 0.87 AI for both. The 3^rd^ ranking for pyrazole **12** with *A. niger* and *A. flavus* of 25.0 and 23.0 mm and AI values of 0.78 and 0.72 in the same sequence. On the other hand, *R. oryzae* exhibited resistance with all the fungal strains investigated.

**Table 2:** IZD for fungal strains administered with pyrazole derivatives following a 72 h incubation period at 28 °C

Pyrazole derivatives (1000 µg/ml)	*A. niger* ATCC 11414		*A. flavus* ATCC 9643		***P. chrysogenum*** ATCC 10106		***R. oryzae*** ATCC 96382	
	IZD (mm)	AI	IZD (mm)	AI	IZD (mm)	AI	IZD (mm)	AI
**1**	0	0	0	0	0	0	0	0
**3a**	0	0	0	0	0	0	0	0
**3b**	32 ± 0.14^a^	1.0	30 ± 0.13^b^	1.0	20 ± 0.11^e^	0.87	0	0
**3c**	0	0	0	0	0	0	0	0
**3d**	0	0	0	0	0	0	0	0
**3e**	0	0	0	0	0	0	0	0
**3f**	0	0	0	0	0	0	0	0
**3g**	18 ± 0.77^f^	0.56	0	0	0	0	0	0
**3h**	0	0	0	0	15 ± 0.25^g^	0.65	0	0
**5**	0	0	0	0	0	0	0	0
**6**	0	0	0	0	0	0	0	0
**7**	0	0	0	0	13 ± 0.11^h^	0.56	0	0
**8**	0	0	0	0	0	0	0	0
**10a**	0	0	0	0	0	0	0	0
**10b**	28 ± 0.12^c^	0.87	0	0	20 ± 0.13^e^	0.87	0	0
**10c**	0	0	0	0	0	0	0	0
**10d**	0	0	0	0	0	0	0	0
**10e**	0	0	0	0	11 ± 0.04^i^	0.48	0	0
**11**	0	0	0	0	0	0	0	0
**12**	25 ± 0.08^f^	0.78	23 ± 0.02^d^	0.72	0	0	0	0
Standard antifungal (Fluconazole 1000 µg/ml)	32.0 ± 0.20^a^		30.02 ± 0.05^b^		23.1 ± 0.06^d^		20.99 ± 0.05^e^	

According to [28], the same-letter variables do not differ much from one another

*AI* Activity index, *mm* Millimeter

### Evaluation of MFIC and MLFC of pyrazole derivatives

**Fig. 2:**
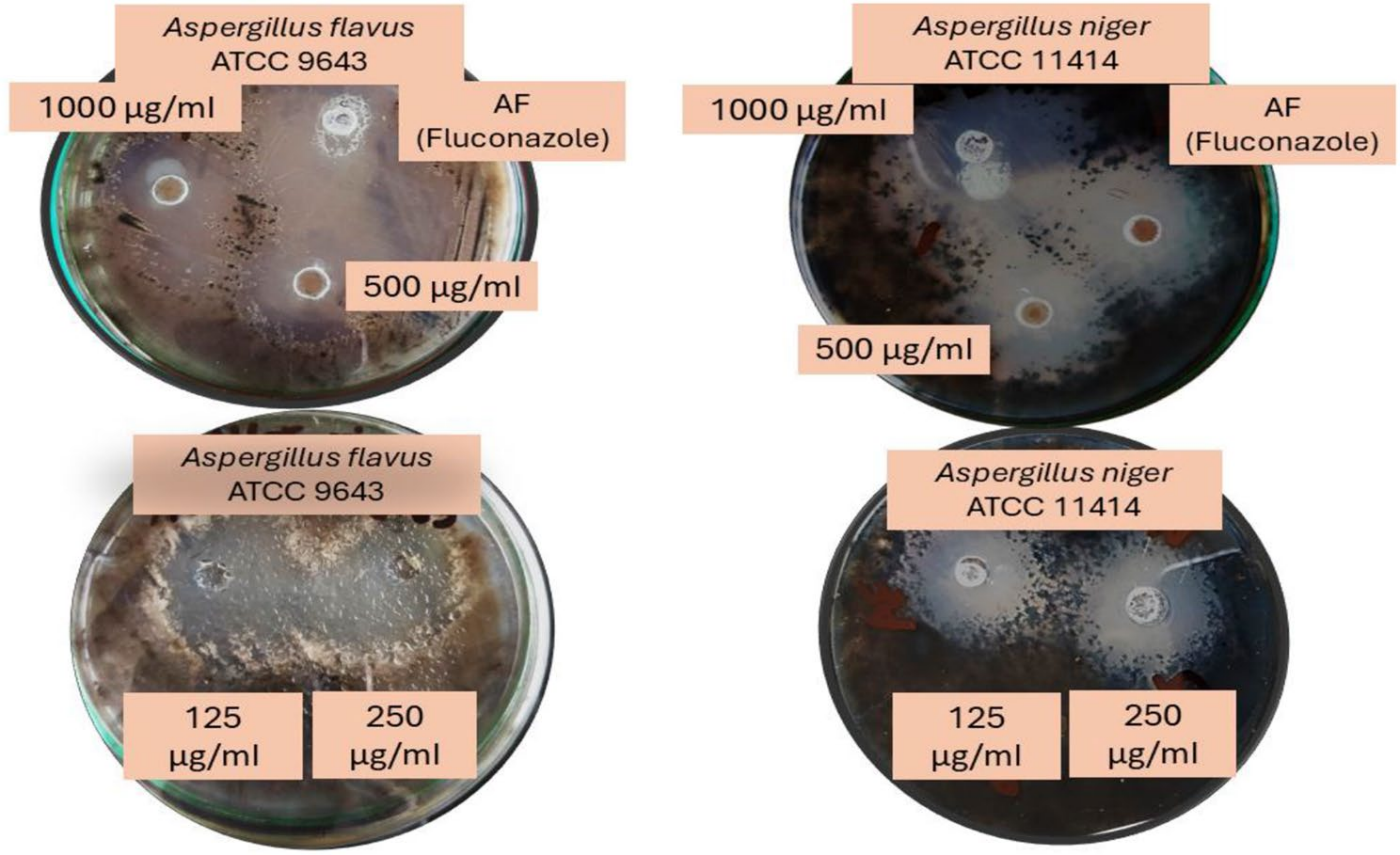
Observation of the compound **3b** treated pathogenic fungal strains’ inhibition zones during a 72 h. incubation period at 28 °C

**Fig. 3:**
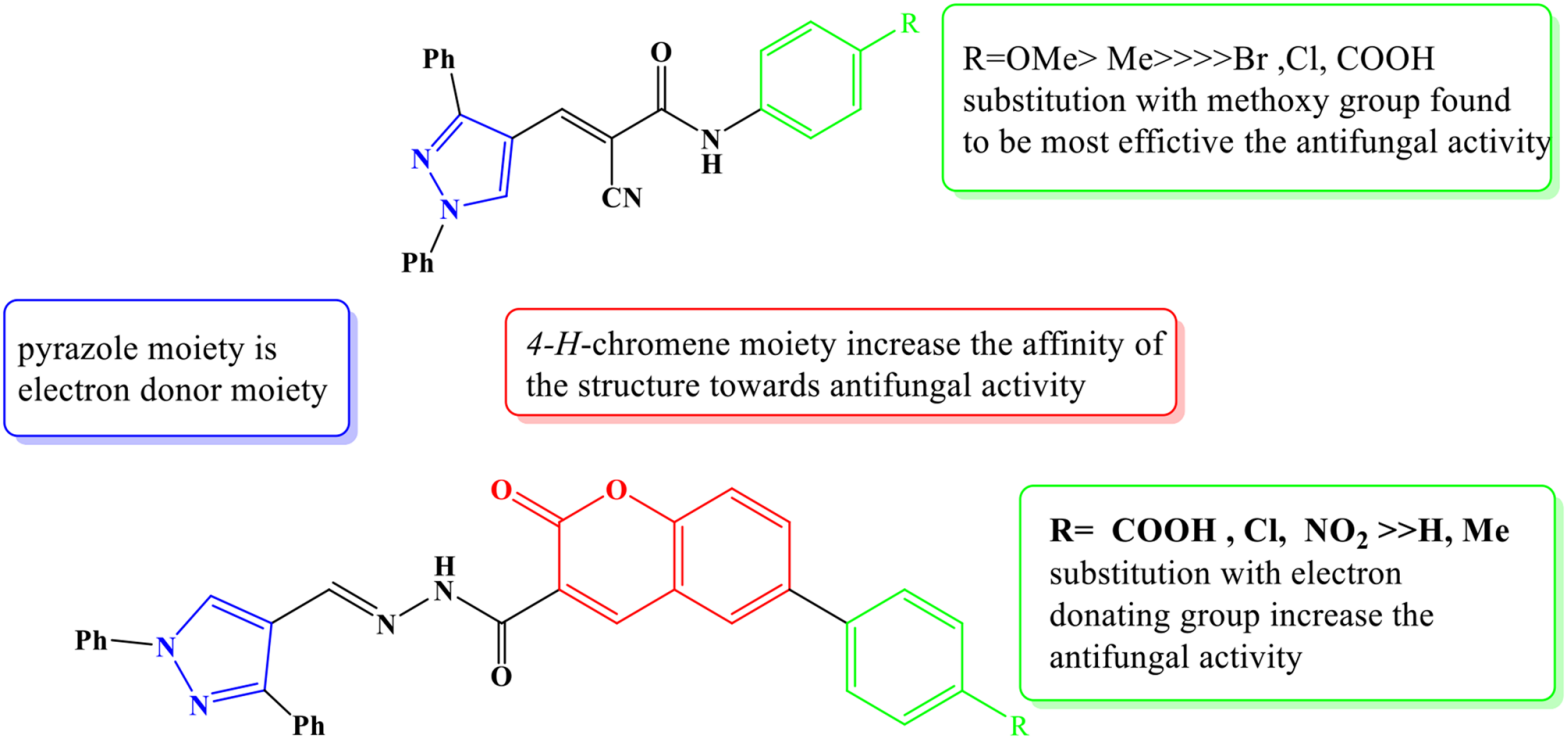
Structure-activity relationship SAR studies of tested compounds against antifungal activities

Data on pyrazole **3b** MFIC values against the studied fungi ranged between 250 to 1000 μg/ml, as demonstrated in Table 3 and Fig. 2. For *A. flavus* and *A. niger*, the MFIC values were at 250 μg/ml. On the other hand, the MLFC values for every pathogenic fungus examined were 500 μg/ml. Between 1000 and 500 μg/ml, the activity spectra were found to have 100% antifungal activity; at doses of 250 μg/ml, the activity rated 100%. Additionally, against the pathogen strains studied, a dose of 125 μg/ml revealed no antifungal action. Figure 3 may be used to explain the compounds’ antifungal activity based on the previous data by altering the kinds of functional groups on benzene rings.

**Table 3:** Minimal fungal inhibitory concentration (MFIC) and minimal lethal fungal concentration (MLFC) of the Pyrazole **3b** compound following a 72 h incubation period at 28 °C

Pyrazole 3b (Conc. µg/ml)	*A. niger* ATCC 11414	*A. flavus* ATCC 9643	Activity spectrum
**Minimum fungicidal inhibition concentration (MFIC)**			
1000	-	-	2/2 (100%)
500	-	-	2/2 (100%)
250	-	-	2/2 (100%)
125	+	+	0/2 (0%)
**Dose of MFIC (µg/ml)**	250	250	
**Minimum lethal fungal concentration (MLFC)**			
1000	-	-	2/2 (100%)
500	-	-	2/2 (100%)
250	**+**	+	0/2 (0%)
125	+	+	0/2 (0%)
**Dose of MLFC (µg/ml)**	500	500	
**Antifungal effect**	**2**	**2**	

- = No detected growth, + = Growth observed. Results according to averages 3 replicates, Fungicide agent = ≤2 and Fungistatic agent = ≥2

### Docking and molecular interaction of pyrazole-synthesized compounds

To validate their effectiveness, molecular docking was used to investigate the binding interactions of all synthesized compounds with protein targets derived from fungal strains with antifungal properties. Table 4 presents the findings of the evaluation of the compounds’ binding affinities to three fungal protein receptors.

**Table 4:** Binding affinity of compounds with targets of antifungal activity

No	Compounds	Affinity (kcal/mol⁻¹)		
		***A. niger***	***A. flavus***	***P. chrysogenum***
		(PDB.ID: 4za5)	(PDB.ID: 6g9v)	(PDB.ID: 1m7g)
1	**10a**	−10.3	−9.0	−9.7
2	**10b**	−16.1	−9.3	−10.5
3	**10c**	7.6	−9.2	−9.0
4	**10d**	7.1	−8.8	−9.6
5	**10e**	7.0	−8.8	−8.9
6	**11**	−11.5	−8.1	−8.8
7	**12**	−13.6	−9.5	−9.0
8	**1**	−9.3	−6.7	−7.7
9	**3a**	−11.4	−8.8	−8.9
10	**3b**	−13.7	−9.7	−10.3
11	**3c**	−10.3	−8.7	−8.3
12	**3d**	−11.1	−8.5	−8.0
13	**3e**	−11.7	−8.6	−8.0
14	**3f**	−11.1	−8.8	−8.6
15	**3g**	−13.5	−8.2	−8.6
16	**3h**	−10.9	−8.7	−9.9
17	**5**	−11.5	−6.8	−7.7
18	**6**	−10.2	−8.1	−8.8
19	**7**	−10.1	−8.4	−10.2
20	**8**	−10.4	−8.0	−8.7
21	Fluconazole	−10.7	−6.6	−6.2

### Docking and interaction with *Aspergillus niger’s* fdc1 (PDB:ID 4ZA5)

**Fig. 4:**
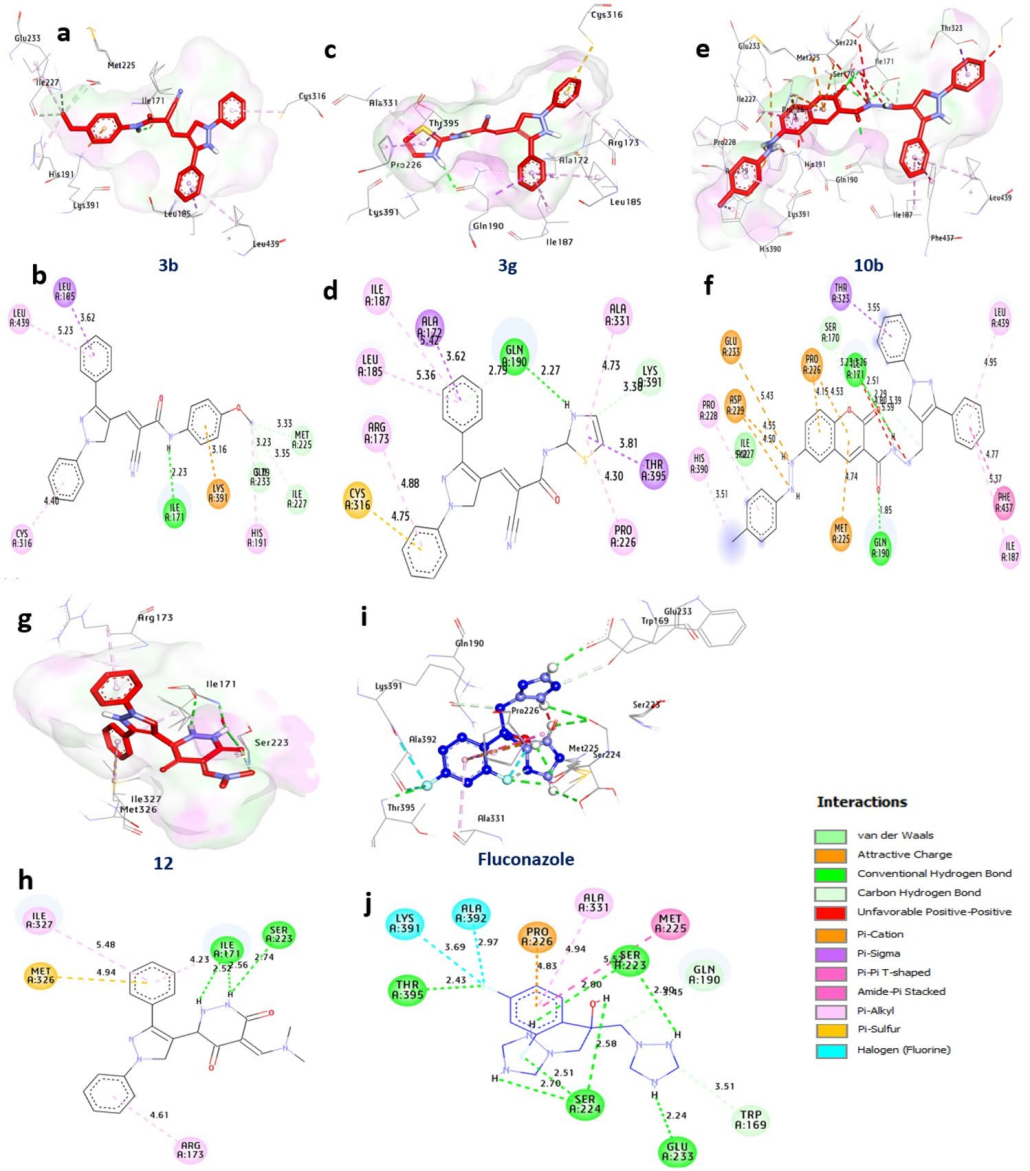
3D-binding of pyrazoles at the inhibitory site of *A.* niger’s fdc1 (PDB: ID 4ZA5). **3b** (**a**, and **b**), **3g** (**c**, and **d**), **10b** (**e**, and **f**), **12** (**g**, and **h**), Fluconazole (**i** and **j**)

The breakdown of aromatic compounds in *A. niger* is associated with the FDC1 protein, which is essential for producing the distinct volatile aromas of fermented liquids like wine and beer. Through docking study, it has been revealed that compounds **3b, 3g, 10b,** and **12** exhibit robust affinities, showcasing binding energies of −13.70, −13.50, −16.10, and −13.60 kcal/mol respectively, surpassing Fluconazole at −10.70 kcal/mol. These compounds establish crucial hydrogen bonds with key amino acids such as Ile171, Gln190, and Ser223, fostering vital interactions. Additionally, non-hydrophilic interactions like alkyl bonds with various amino acids, Pi-sigma bonding with others, Pi-sulfur interactions, Amid-Pi-stack interactions, Carbon-H bonding, and Pi-cation interactions contribute to the intricate binding network. Notably, the residues Ile171, Gln190, and Ser223 within the catalytic site positively influence the binding affinity of these compounds. In conclusion, the data strongly suggests that compounds **3b, 3g, 10b,** and **12** show considerable potential for further investigation as possible inhibitors of fungal FDC1 in *A. niger* (Table 5 and Fig. 4).

**Table 5:**
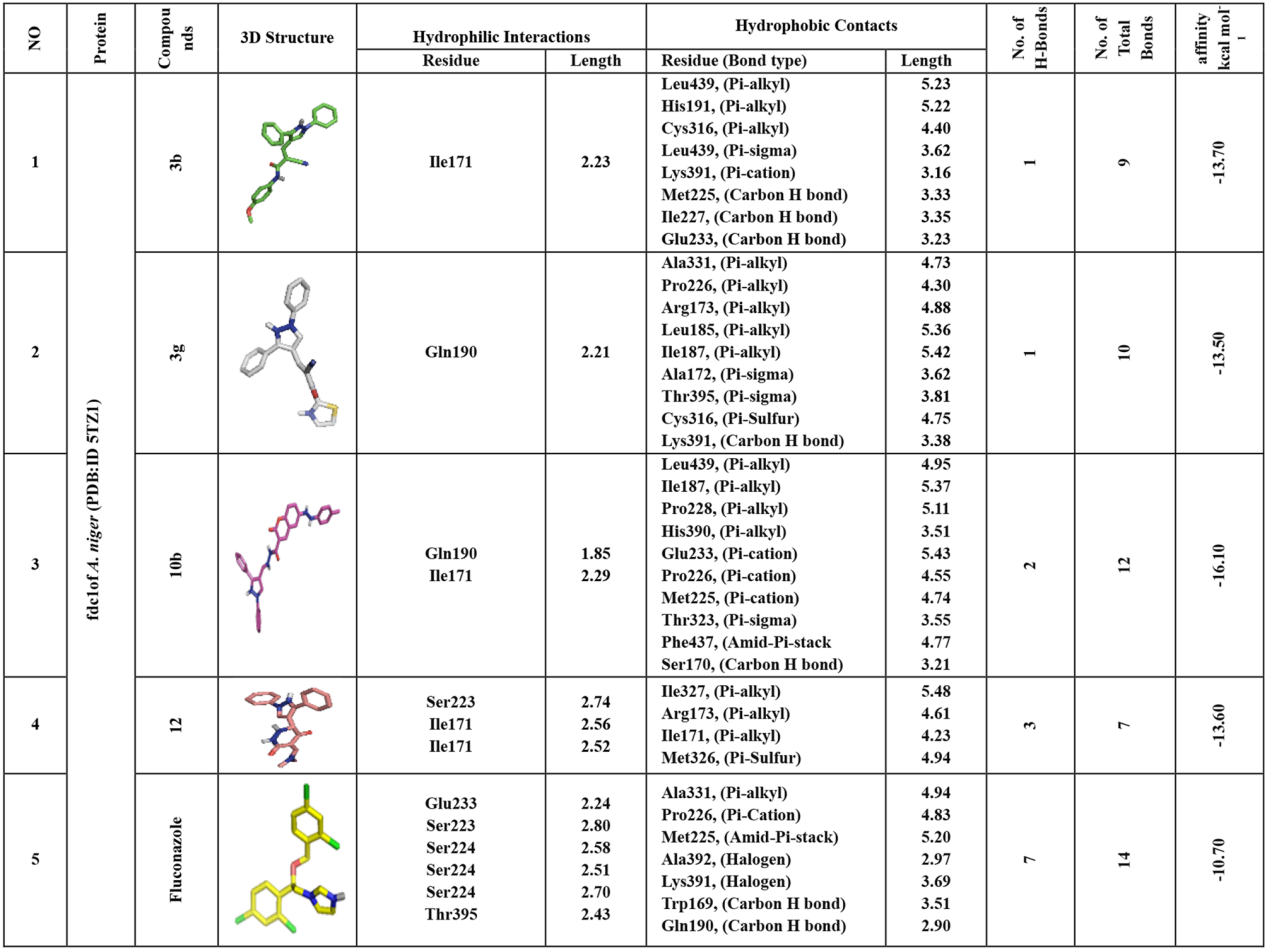
Interactions of pyrazoles **3a**, **3g**, **10b**, and **12** with *A.niger’s* fdc1 (PDB: ID 4ZA5)

### Docking and interaction studies with *A. flavus’s* UDP-*N*-acetyl glucosamine

**Fig. 5:**
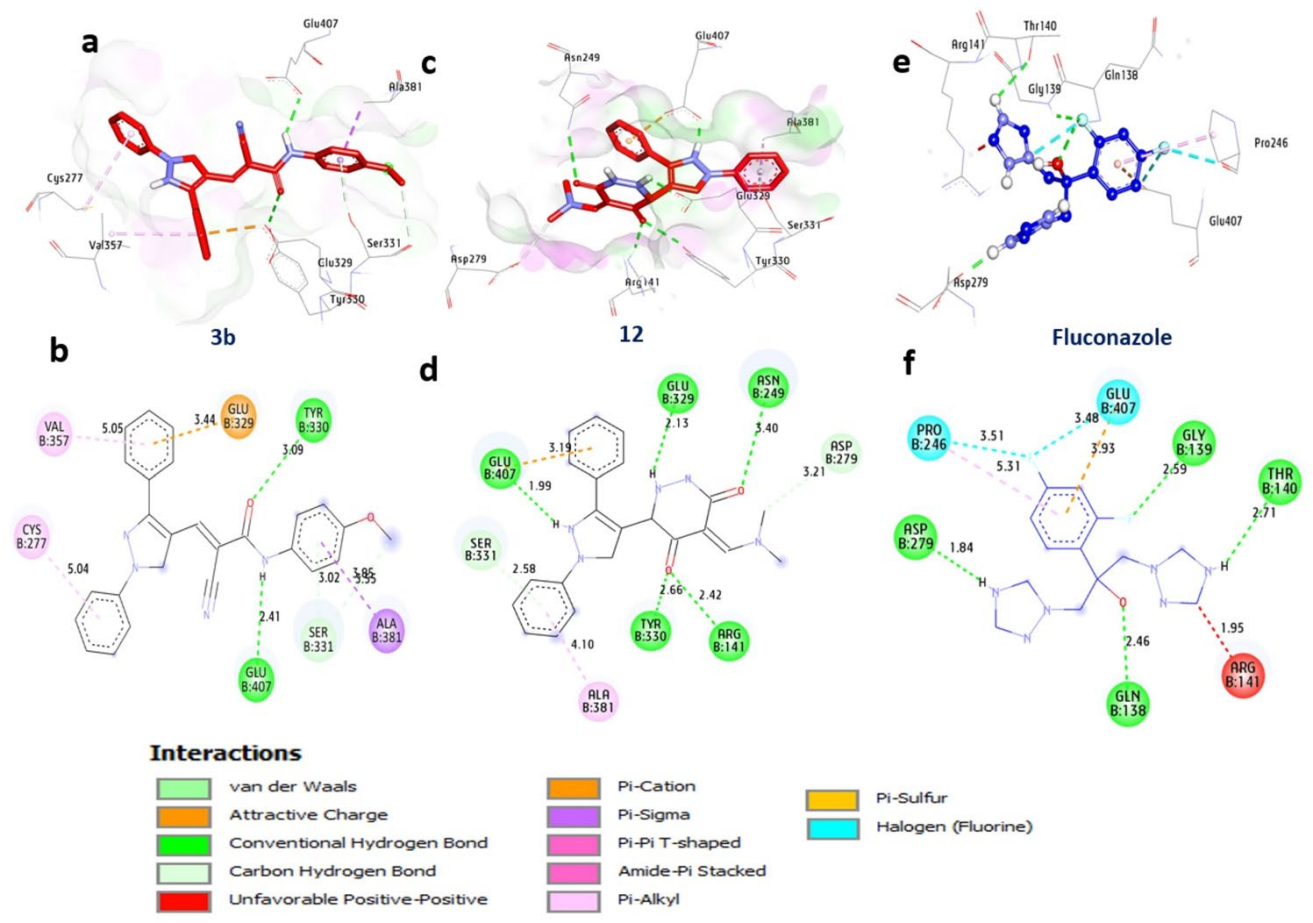
3D-binding of pyrazoles at the inhibitory site of *A. flavus’s* UDP-*N*-acetylglucosamine (PDB: ID 6G9V): **3b** (**a**, and **b**), **12** (**c**, and **d**), Fluconazole (**e** and **f**)

UDP-N-acetylglucosamine is an essential component in the manufacture of chitin and β-(1,3)-glucan, two crucial components of fungal cell walls, and it is involved in several biological processes and metabolic pathways. As per the docking outcomes, compounds **3b** and **12** exhibit notable affinities, boasting binding energies of −9.70 and −9.50 kcal/mol, surpassing Fluconazole at −6.60 kcal/mol. These compounds establish hydrogen bonds with significant amino acids, including Arg141, Tyr330, Glu329, Asn249, and Glu407, fostering essential interactions. Furthermore, non-hydrophilic interactions such as alkyl bonds with Val357, Cys277, and Ala381, Pi-Sigma bonding with Ala381, Carbon-H bonding with Ser331 and Asp279, and Pi-cation interactions with Glu407 and Glu329 contribute to the intricate bonding network. The beneficial effect of the active site residues Tyr330, Glu407, Ala381, and Glu329 on the compounds’ binding affinity is noteworthy. In summary, the results clearly support compounds **3b** and **12** as possible and promising UDP-N-acetylglucosamine inhibitors in *A. flavus* (Table 6 and Fig. 5).

**Table 6:**
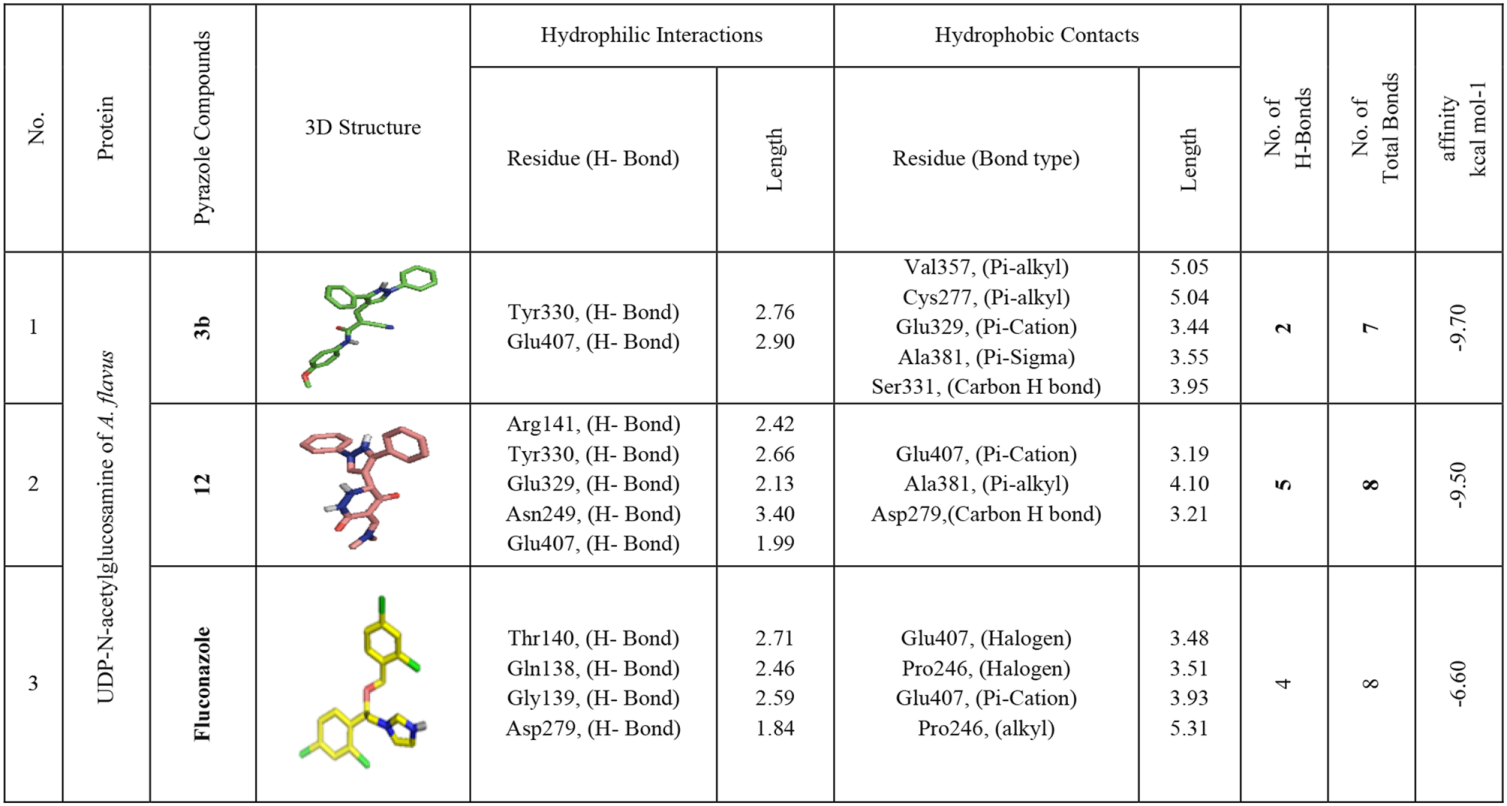
Interactions of pyrazoles **3a**, **3g**, **10b**, and **12** with *A. flavus’s* UDP-*N*-acetylglucosamine (PDB:ID 6G9V)

### Docking and interaction with *P. chrysogenum’s* Adenosine 5’-phosphosulfate kinase

**Fig. 6:**
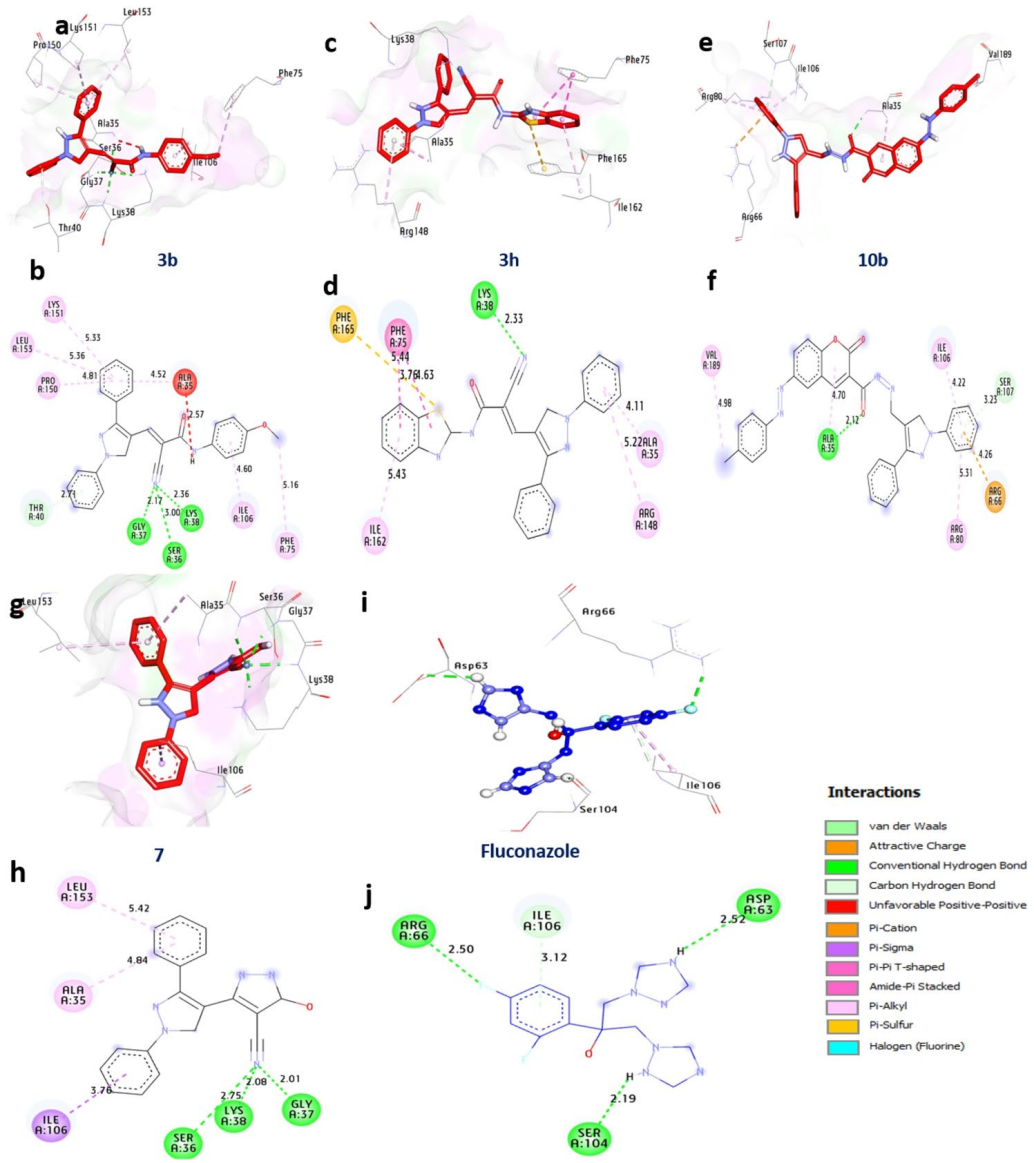
3D-binding of pyrazoles at the inhibitory site of *P. chrysogenum’s* Adenosine 5′-phosphosulfate kinase (PDB: ID 1M7G). **3b** (**a**, and **b**), **3h** (**c**, and **d**), **10b** (**e**, and **f**), **7** (**g**, and **h**) Fluconazole (**i** and **j**)

In many different organisms, the enzyme adenosine 5-phosphosulfate (APS) kinase is responsible for metabolizing compounds that include sulfur. The findings from molecular docking reveal that compounds **3b, 3 h, 10b,** and **7** exhibit significant binding affinities, with binding energies values −10.3, −9.90, −10.5, and −10.2 kcal/mol respectively, surpassing that of Fluconazole at −6.20 kcal/mol. These compounds establish hydrogen bonds with pivotal amino acids, including Gly37, Lys38, Ser36, Ala35, and Gly37, forming essential interactions. Additionally, non-hydrophilic interactions such as alkyl bonds with Pro150, Leu153, and others, Pi-sulfur interactions with Phe165, and various other bond types contribute to the bonding network. Interestingly, the active site residues Gly37, Lys38, Ala35, and Ile106 have a beneficial effect on these compounds’ binding affinities. As a result, chemicals **3b, 3h, 10b**, and **7** stand out as viable options for additional investigation as possible inhibitors of *P. chrysogenum*’s Adenosine 5’-phosphosulfate kinase (Table 7 and Fig. 6).

**Table 7:**
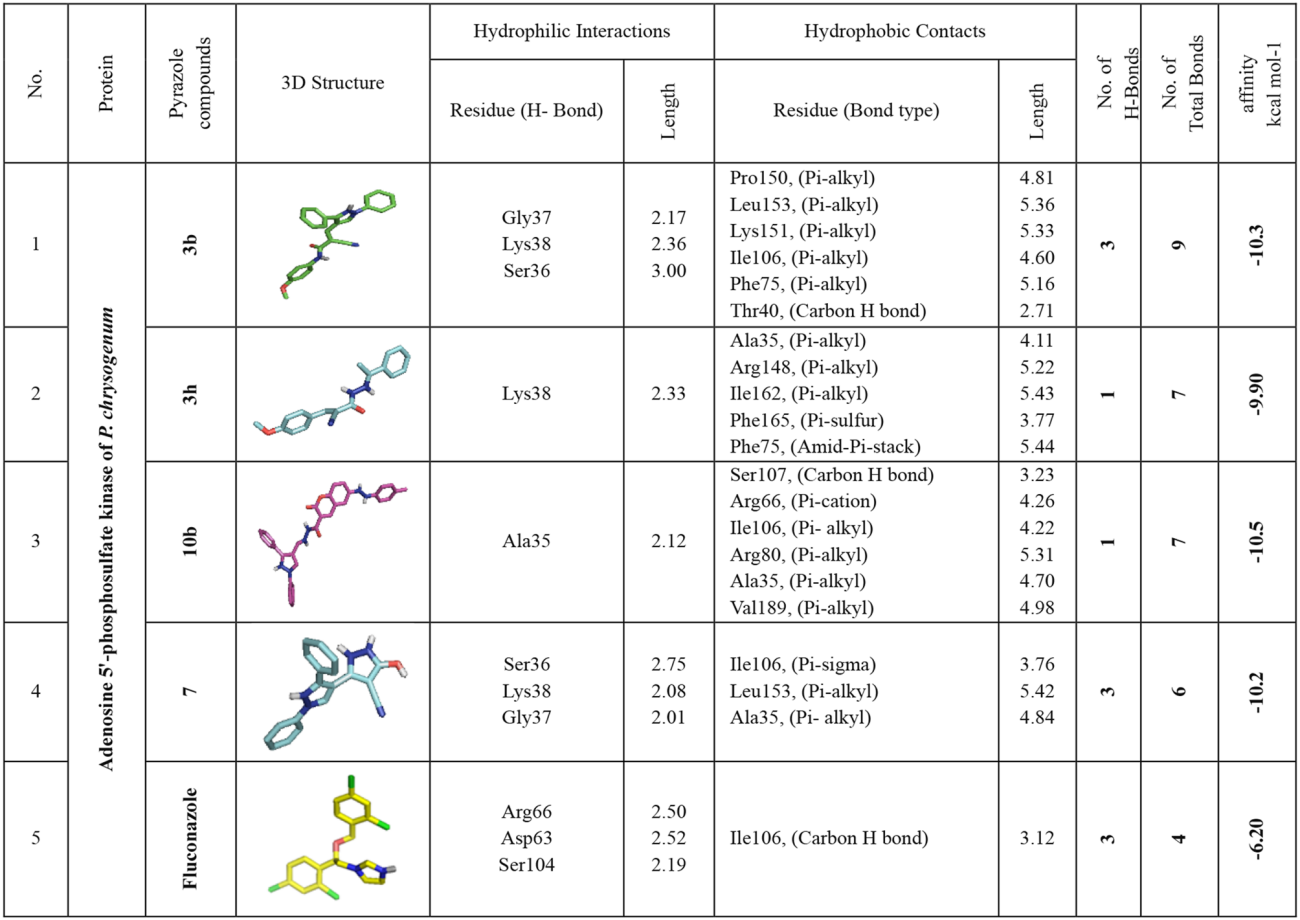
Interactions of pyrazoles **3a**, **3g**, **10b**, and **12** with *P. chrysogenum’s* Adenosine 5′-phosphosulfate kinase

### In silico pharmacokinetics ADME prediction of synthesized compounds

**Fig. 7:**
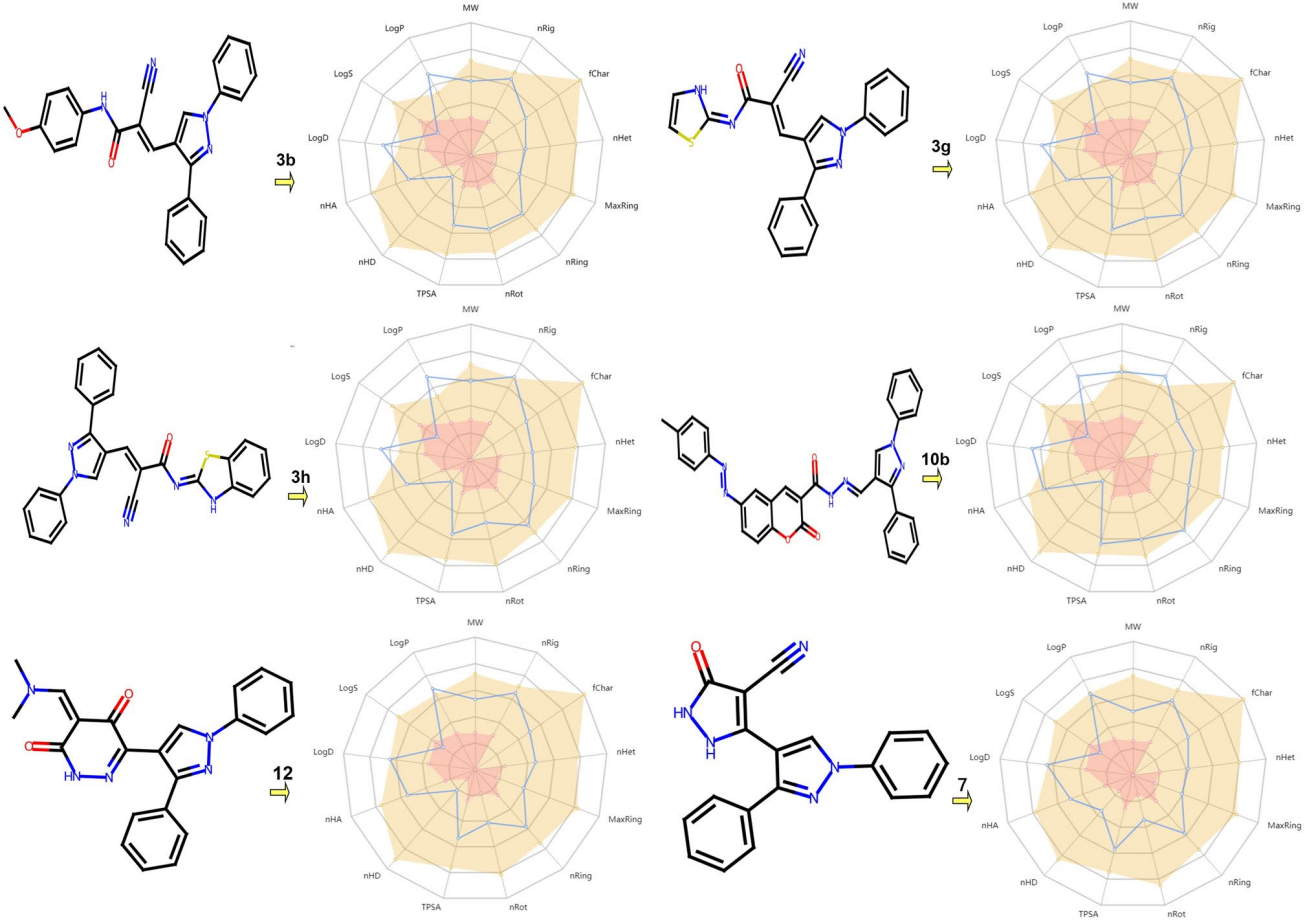
Graph of oral bioavailability for pyrazole substances

The most promising compounds (**3b, 3g, 3h, 10b, 12,** and **7**) were identified based on their molecular docking results, considering their high affinity along with ADME and toxicity profiles. First, Table 8 and Fig. 7 display the testing substances’ physiochemical characteristics. We looked at and assessed every physiochemical criterion. As a result, all compounds showed considerable structural flexibility and enough rotatable bonds (RBs 3–8). All compounds had less than 10 hydrogen bond acceptors (HBA) and less than 5 HBD, indicating a favorable balance of HBD and HBA and a better probability of oral bioavailability. Additionally, the compounds’ TPSA values were comparatively high, lying between 60 to 140, which is the ideal range for both oral bioavailability and gastrointestinal absorption. Secondly, compounds **3b**, **3g**, **3h**, **10b**, **7**, and **12** were further assessed for lipophilicity and water solubility. The results demonstrated that all active compounds were highly soluble in water, with Log S values ranging from −3.876 to −5.341, demonstrating high water solubility. These properties make it easier to synthesize, handle, and formulate these bioactive compounds. Thirdly, pharmacokinetic experiments showed that the compounds had a remarkable theoretical accessibility, indicating that they may develop into drug-like substances. However, there was evidence of substantial intestinal absorption and the possibility of medication interactions due to the inhibition of the CYP1A2, CYP2C19, and CYP2C9 enzymes. Fourth, analyses using the Lipinski, Golden Triangle, and Pfizer guidelines showed that every molecule had the physicochemical characteristics needed for drug development and satisfied the drug-likeness requirements. All drugs showed more than 99% binding, according to the assessment of plasma protein binding (PPB), which suggests a low therapeutic index and a low proportion of unbound plasma. Additionally, all compounds were anticipated to be BBB- and incapable of passing through the blood-brain barrier, improving their safety profiles. Lastly, Table 9 shows that these chemicals appear to be non-toxic and reasonably safe based on computational evaluations.

**Table 8:** Pharmacokinetics and physicochemical properties prediction of pyrazole compounds

	ID	3b	3g	3h	10b	12	7		ID	3b	3g	3h	10b	12	7
**Physicochemical Properties**	MW	420.16	397.1	447.12	552.19	385.15	327.11	**Metabolism**	CYP1A2-inh	0.344	0.867	0.677	0.169	0.943	0.931
	Vol	446.047	400.215	455.569	570.244	395.769	335.091		CYP1A2-sub	0.097	0.07	0.059	0.077	0.925	0.052
	Dense	0.942	0.992	0.981	0.968	0.973	0.976		CYP2C19-inh	0.714	0.951	0.851	0.517	0.578	0.886
	nHA	6	6	6	9	7	6		CYP2C19-sub	0.071	0.064	0.06	0.064	0.346	0.048
	nHD	1	1	1	1	1	2		CYP2C9-inh	0.954	0.95	0.966	0.876	0.673	0.894
	TPSA	79.94	86.83	86.83	114.21	79.59	90.26		CYP2C9-sub	0.849	0.442	0.112	0.77	0.079	0.965
	nRot	7	5	5	8	4	3		CYP2D6-inh	0.03	0.483	0.093	0	0.001	0.594
	nRing	4	4	5	6	4	4		CYP2D6-sub	0.539	0.094	0.063	0.422	0.094	0.219
	MaxRing	6	6	9	10	6	6		CYP3A4-inh	0.579	0.619	0.591	0.18	0.135	0.707
	nHet	6	7	7	9	7	6		CYP3A4-sub	0.763	0.533	0.721	0.704	0.46	0.194
	fChar	0	0	0	0	0	0	**Excretion**	CL (Clearance)	7.504	5.581	4.283	1.622	5.135	4.876
	nRig	26	26	31	38	26	24		T12	0.086	0.178	0.058	0.044	0.044	0.282
	Flex	0.269	0.192	0.161	0.211	0.154	0.125	**Toxicity**	hERG Blockers	0.057	0.02	0.008	0.064	0.027	0.03
	nStereo	0	0	0	0	0	0		H–HT	0.847	0.988	0.991	0.96	0.746	0.975
**Solubility**	LogS	−6.972	−5.685	−6.75	−9.21	−4.963	−5.236		DILI	0.992	0.997	0.997	0.993	0.996	0.997
	LogD	4.276	4.087	4.587	4.438	3.008	3.026		AMES Toxicity	0.256	0.065	0.16	0.239	0.066	0.11
	LogP	5.083	4.065	5.193	7.171	3.486	2.93		Rat Oral Acute Toxicity	0.271	0.38	0.187	0.042	0.227	0.023
	ESOL Log S	−2.45	−2.99	−2.18	−2.75	−3.12	−2.44		FDAMDD	0.92	0.934	0.908	0.921	0.961	0.836
	Ali Log S	−6.111	−6.210	−7.400	−7.321	−6.210	−6.210		Skin Sensitization	0.054	0.044	0.031	0.326	0.041	0.097
	Silicon-IT class	Soluble	Soluble	Soluble	Soluble	Soluble	Soluble		Carcinogenicity	0.302	0.057	0.592	0.797	0.177	0.046
**Drug-likeness**	Lipinski Rule	Accepted	Accepted	Accepted	Rejected	Accepted	Accepted		Eye Corrosion	0.003	0.003	0.003	0.003	0.003	0.003
	Pfizer Rule	Accepted	Accepted	Accepted	Accepted	Accepted	Accepted		Eye Irritation	0.279	0.175	0.489	0.227	0.019	0.68
	Golden Triangle	Accepted	Accepted	Accepted	Rejected	Accepted	Accepted		Respiratory Toxicity	0.146	0.804	0.298	0.057	0.801	0.954
**Absorption**	Pgp-inh	0.996	0.841	0.966	0.998	0.951	0.857	**Toxicophoric rules**	N-G Carcinogenicity	0	0	0	0	0	0
	Pgp-sub	0	0	0	0.002	0	0		LD50_oral	0	0	0	0	0	0
	HIA	0.022	0.645	0.047	0.014	0.007	0.015		Genotoxic Carcinogenicity	1	0	0	6	1	0
	F (20%)	0.006	0.025	0.007	0.447	0.002	0.002		Sure ChEMBL	0	0	0	1	0	0
	F (30%)	0.006	0.01	0.006	0.993	0.003	0.001		Non-Biodegradable	0	0	0	1	2	0
	Caco-2	−5.025	−4.844	−4.962	−4.852	−4.659	−5.407		Skin Sensitization	5	1	1	1	1	0
	MDCK	8.21E-06	1.63E-05	1.13E-05	1.30E-05	2.27E-05	1.08E-05		Aquatic Toxicity Rule	4	3	3	1	3	0
Distribution	BBB	0.131	0.284	0.26	0.002	0.51	0.262	**Medicinal Chemistry**	Toxicophores	4	4	4	6	4	2
	PPB	99.93%	98.67%	99.88%	107.22%	97.59%	98.63%		QED	0.348	0.418	0.307	0.098	0.552	0.606
	VDss	0.603	0.418	0.515	1.222	0.579	0.416		Synth	2.141	3.009	2.823	2.759	2.774	2.532
	Fu	0.82%	0.68%	0.53%	0.53%	1.47%	1.54%		Fsp3	0.038	0	0	0.03	0.091	0

*MW* Molecular weight, *nRig* number of rigid bonds, *fChar* formal charge, *nHet* number of heteroatoms, *MaxRing* number of atoms in the largest ring, *nRing* number of rings, *nRot* number of rotatable bonds, *TPSA* topological polar surface area, *nHA* number of hydrogen bond acceptors, *nHA* number of hydrogen bond donors, *logS* Log of the aqueous solubility, *logP* Log of the octanol/water partition coefficient and *logD* logP at physiological pH 7.4

**Table 9:** Prediction of pyrazole chemical toxicity risks and outcomes

No	Pyrazole compounds	Toxicity risks				Physicochemical properties					
		Mutagenic	Tumorigenic	Irritant	Reproductive	CLogP	Solubility	Molecular Weight	TPSA	Drug likeness	Drug score
1	**3b**	(−)	(−)	(−)	(−)	3.84	−5.30	420.0	79.94	0.51	0.44
2	**3g**	(−)	(−)	(−)	(−)	2.78	−5.40	397.0	108.3	1.17	0.52
3	**3h**	(−)	(−)	(−)	(−)	3.39	−6.68	447.0	108.3	1.75	0.41
4	**10b**	(+)	(+)	(−)	(+)	6.18	−8.41	552.0	110.3	−4.51	0.02
5	**12**	(−)	(−)	(−)	(−)	1.10	−3.63	385.0	79.59	7.4	0.64
6	**7**	(−)	(−)	(−)	(−)	0.50	−3.90	327.0	82.74	1.47	0.72

### Molecular dynamics simulation (MDS)

**Fig. 8:**
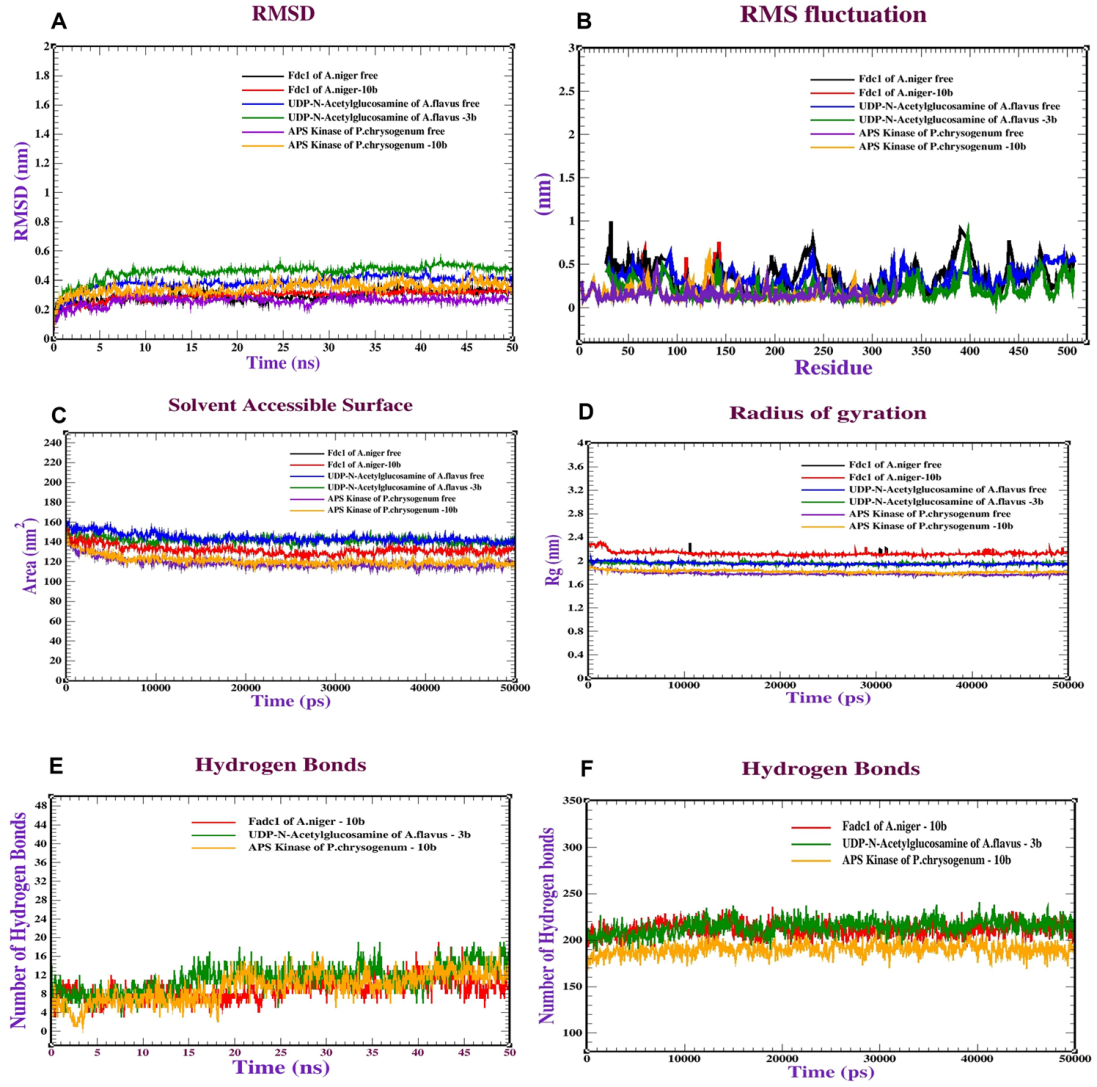
Dynamics simulations of *A. niger*’s fdc1 (PDB: 4ZA5), *A. flavus*’s UDP-N-acetylglucosamine, and *P. chrysogenum*’s APS kinase complexed with **10b** and **3b**: (**A**) RMSD, (**B**) RMSF, (**C**) SASA, (**D**) Rg, and (**E** and **F**) Intermolecular and intramolecular H bonds

Based on the docking results of three antifungal activity receptors with the promising compounds **10b** and **3b**, dynamic simulations were conducted to explore the behavior and stability of the protein complex at the atomic level. Various analyses were carried out on the MDS complex with **10b** and **3b** to evaluate the stability and dynamics of these complexes. The RMSD was utilized to assess the stability of protein structures. For instance, the stability of the fdc1 complex of *A. niger* (PDB: 4ZA5) with **10b** was observed to range between 0.20–0.25 nm, with stabilization occurring after 15 ns. Similarly, the UDP-N-acetylglucosamine of *A. flavus* (PDB: ID 6G9V) complexed with **3b** exhibited steady RMSD values within the range of 0.40–0.45 nm, reaching a stable state after 20 nm. The APS kinase of *P. chrysogenum* complexed with compound **10b** also demonstrated stability, with RMSD ranging from 0.20–0.30 nm and reaching stability after 30 ns. Additionally, RMSF analysis was used to assess the flexibility of amino acid residues during the simulation; the majority of residues had very minor variations (0.1–0.7 nm), suggesting relative stability. The fdc1 complex of *A. niger*, UDP-N-acetylglucosamine of *A. flavus*, and APS kinase of *P. chrysogenum* complexes had Rg values ranging from 2.20–2.35 nm, 1.90–2.00 nm, and 1.75–1.95 nm, respectively. Rg analysis was performed to evaluate the general shape of the protein complexes. These numbers shed light on how compact or expansive the protein structures were during the simulation. *A. niger*’s fdc1 complex, *A. flavus*’s UDP-N-acetylglucosamine, and *P. chrysogenum*’s APS kinase all had SASA values between 135 and 145 nm^2^, 155 and 165 nm^2^, and 115 and 130 nm^2^, respectively. Additionally, SASA analysis was carried out to comprehend the stability and kinetics of protein folding. Finally, the stability of the proteins was assessed by examining the development and variation of intramolecular and intermolecular hydrogen bonds. While intermolecular hydrogen bonds displayed varying degrees of interactions, intramolecular hydrogen bonds within the fdc1 complex of *A. niger*, UDP-N-acetylglucosamine of *A. flavus*, and APS kinase of *P. chrysogenum* complexes varied within specific ranges (1–10 bonds), which greatly contributed to the stability of the complex structures (Fig. 8).

### Biocompatibility of the most active pyrazole compound

**Fig. 9:**
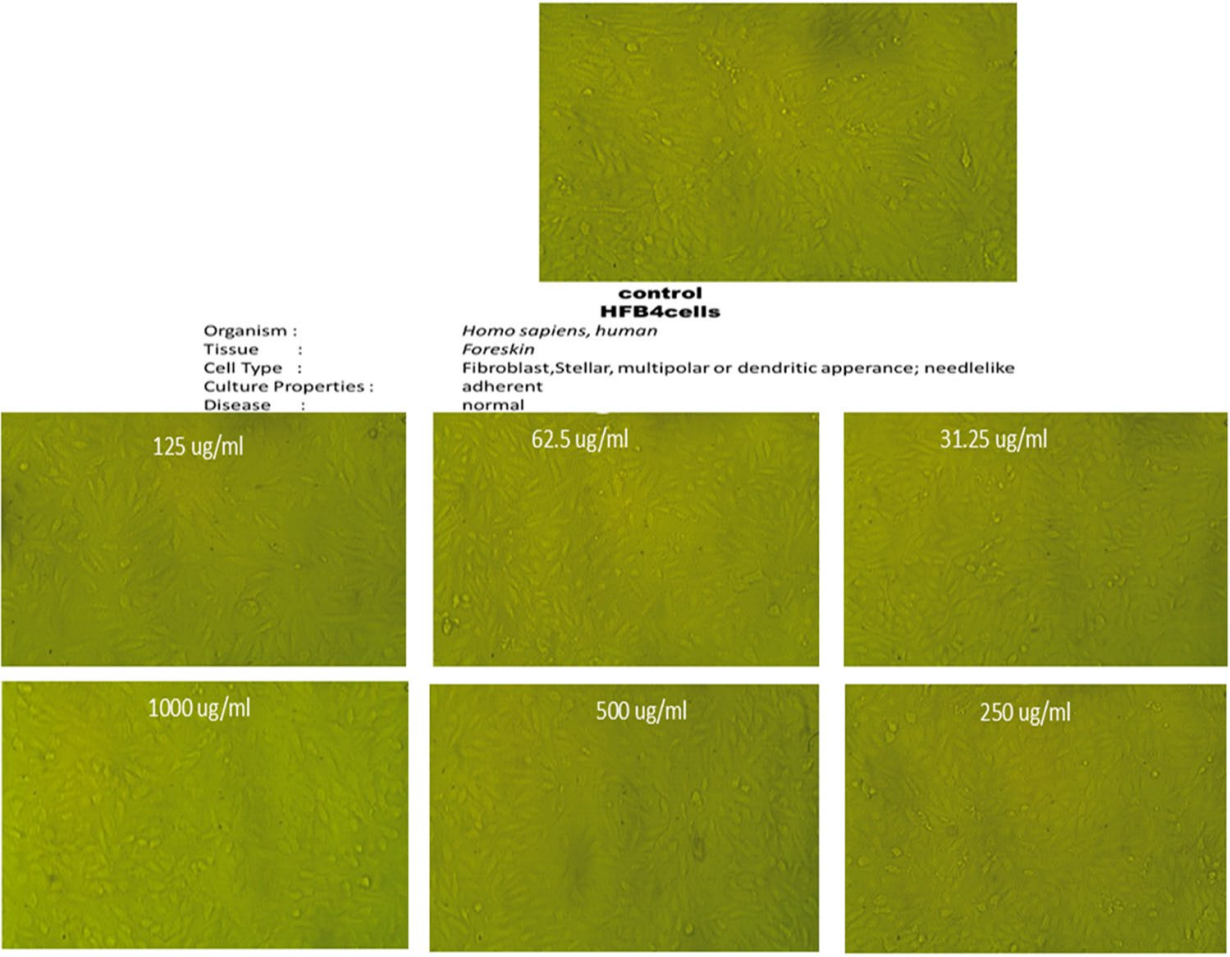
Microscopic images for HFB4 normal human skin cell line prior and after exposure to various doses of pyrazole **3b**

The biocompatibility investigation of the most active pyrazole compound (**3b**) using HFB4 normal human skin cell line showing no significant changes in the different concentrations ranged between 31.25–1000 µg/ml (Fig. 9 and Table 1^0^).

**Table 10:** HFB4 normal human skin cell line viability affected by various concentrations of pyrazole **3b**

ID	µg/ml	O.D			Mean O.D	±SE	Viability %	Toxicity %	IC_50_ ± SD
HFB4	——–	0.568	0.561	0.566	0.565000	0.002082	100	0	µg/ml
**3b**	1000	0.569	0.560	0.560	0.563000	0.003000	99.23303835	0.766961652	—–
	500	0.565	0.566	0.562	0.564333	0.001202	99.64601770	0.353982301	
	250	0.564	0.567	0.562	0.564333	0.001453	99.76401180	0.235988201	
	125	0.563	0.567	0.561	0.563667	0.001764	99.88200590	0.117994100	
	62.5	0.561	0.567	0.564	0.564000	0.001732	99.82300885	0.117994100	
	31.25	0.558	0.564	0.560	0.560667	0.001764	99.88200590	0.176991150	

## Discussion

The continuous development of novel classes of fungicide active compounds with high efficacy, low toxicity, minimal residue, and broad-spectrum biological activity is currently a top priority in research and development [1, 3, 7, 29]. Pyrazole derivatives, target structures that have been developed in the interim, exhibit a broad spectrum of biological activities, such as antituberculosis, antibacterial, antifungal, and anti-inflammatory characteristics. They have attracted a lot of attention since they are an important class of compounds for the creation of medications. Numerous studies have verified the antifungal efficacy of pyrazole derivatives, according to [13, 30, 31]. The first broad-spectrum oral imidazole-based antifungal medication was ketoconazole [32, 33]. The first oral triazole derivative with anti-Aspergillus qualities was itraconazole [34, 35]. For the prevention and treatment of superficial and invasive fungal infections, doctors frequently prescribe fluconazole, another azole antifungal with two triazole rings [36]. Fluconazole is an excellent antifungal drug for control because of its distinct structural features, which also provide it a superior absorption rate than other azoles [37]. Voriconazole is another triazole antifungal that works well for treating invasive aspergillosis in the first line, especially against species that are fluconazole-resistant [38].

With IZDs of 20.5 mm and 18.3 mm against *A. fumigatus* and *A. clavatus*, respectively, the presence of 3-methoxyphenyl and phenyl (45) at nitrogen atoms in the core ring offered greater antifungal activity [39]. In the study of [40], employed a series of triazole derivatives, such as 1,2,3-benzotriazin-4-one ring, and found that most of the compounds had more potent antifungal effects *in vitro* than fluconazole; compound 6, which exhibited good antifungal activities against *A. fumigatus, A. niger*, and *A. flavus* with a minimum inhibitory concentration of 250 μg/ml, was one such compound. On the other hand, fluconazole derivatives called Nitrotriazole did not exhibit any sensitivity to any *Aspergillus* species [41]. whereas pyrazole compounds **2–5**, with MICs of 750, 92, and 82 µg/ml, respectively, were more effective against *A. niger*.

As with *Penicillium marneffei*, all of the synthetic pyrazole compounds by [42] demonstrated intense activity, with the exception of **2a–c, 6a, 8** and **9a. 10** and **11** as well as **3a, 3c**, were only moderately active against *A. fumigatus*, whereas **3a, 3d–f, 6b, 7, 9b–c**, and **2e, 3** were more active. Compounds **2e, 10** and **11** had the maximum activity against *A. flavus*, whereas compounds **3b–c, 3e, 6b**, and **9c** showed the lowest activity. Using docking to evaluate the inhibitory interaction between a molecule and a Penicillin-binding protein [43], molecular docking was utilized to clarify the binding interactions of inhibitors with β-lactamase and FabH enzyme targets [44], and our *in silico* results provide strong evidence that compounds **3b** and 12 are effective inhibitors of UDP-N-acetylglucosamine in *A. flavus*. We also found that compounds **3b, 3h, 10b,** and **7** have a higher binding affinity for the APS kinase of *P. chrysogenum*. These findings align with [45] which identified and synthesized compounds with potential as kinase inhibitors. Compound **5d** demonstrated 70.82% inhibition of topoisomerase IIα at 100 μM, with a maximum docking score of −8.24. According to ADME prediction results, the majority of these compounds had *in silico* drug-likeliness characteristics that fell within the optimal range [46].

They also revealed that the ligand **5d** of the parent analogue, which contained a Br group at the m-substituted phenyl, exhibited hydrophobic interaction with residues Gly161, Gly166, Tyr165, Arg98, Ser149, Ile141, Ile125, Phe142, Val137, Asn95, and Asn91, in addition to H-bonding with these amino acid residues. With a docking score of −8.24 and a glide energy of −59.08 kcal mol^−1^, this ligand produced the best results. The other ligands have similar glide energies and moderate docking scores. In the docking investigations, the topoisomerase IIα was based on the X-ray crystal structure of the human topoisomerase IIα (1ZXM) ATPase domain. Because of their ability to prevent binding to the ATP Binding catalytic site of 1ZXM, the majority of the potential inhibitors that have been found fall into the category of catalytic inhibitors. The majority of these substances possessed physiochemical characteristics that were within the ideal range, according to the results of ADME studies [47].

Compound **3b** showed high safety with no IC_50_ dose was determined. In the same trend, the IC_50_ values, the majority of **5d, 5e, 5f, 5g, 6d, 6e,** and **6g** exhibited modest cytotoxicity toward normal human embryonic kidney cells and moderate to excellent cytotoxicity against cell lines representing breast, cervical, and lung cancer [47]. Analogs **5d, 5e, 5f, 5g, 6d, 6e**, and **6g** shown significant cytotoxicity in comparison to the reference drug, etoposide. To ascertain the effect of the substituents on the cytotoxic properties of the produced compounds, we subsequently conducted structure activity relationship (SAR) studies. Compounds with a meta substituted Br group on the benzene ring showed the most cytotoxic activity, whereas compounds with substituents Br and NO_2_ on the benzene ring showed beneficial benefits. In malignant cell types, substances having a meta substituted electron-withdrawing group exhibit a deadly action sequence where Br > NO_2_. The cytotoxic strength of compounds exhibiting para substituted electron-withdrawing in malignant cell lines is NO_2_ > Br > F > Cl. To sum up, for the malignant cell line, compounds having an electron-donating substituent in the para position of the benzene ring show a cytotoxic strength order of OCH_3_ > CH_3_ [48].

## Conclusions

The increasing microbial resistance to antibiotics has led to a need for innovative fungicides. The study investigated twenty pyrazole derivatives as antifungal agents, with pyrazole **3b** being the most effective against *A. niger* and *A. flavus*. Pyrazole **3b** showed 100% antifungal activity and 50% at doses of 250 μg/ml. The biocompatibility of the compounds was confirmed using HFB4 normal human skin cell line. The compounds displayed strong binding energies with key proteins in fungi, suggesting their potential to hinder enzyme activity and demonstrate antifungal properties. These results lend credence to the chemicals’ potential for use in future research on medication development. It could be an alternative safe and biocompatible with no side effects like antifungal formulas such as spray, emulsion, and cream which could be approved with extensive study. Future research should expand the pyrazole compounds library to identify more potent antifungal agents. Testing against a wider range of fungal pathogens, including those resistant to existing treatments, is crucial for clinical trials. In vivo studies, mechanistic studies, and formulation development are also essential. Long-term toxicity studies and combination therapies could help overcome resistance. Addressing these limitations and pursuing future research directions will help realize the potential of pyrazole derivatives as effective antifungal agents.

## Electronic supplementary material

Below is the link to the electronic supplementary material.

Supplementary Material 1

## Data Availability

The following strain suppliers received all of the microbial strains from the Agricultural Microbiology Department of the Faculty of Agriculture at Ain Shams University in Cairo, Egypt. (a) *Aspergillus niger* ATCC 11414 https://www.atcc.org/products/11414. (b) *A. flavus* ATCC 9643 https://www.atcc.org/products/9643. (c) *Rhizopus oryzae* ATCC 96382 https://www.atcc.org/products/96382. (d) *Penicillium chrysogenum* ATCC 10106 https://www.atcc.org/products/10106.

## Abbreviations

AcOH: Acetic acid
ADMET: Absorption–distribution–metabolism–excretion–toxicity
EtOH: Ethanol
IR: Infrared spectroscopy
MS: Mass spectrometry
MFIC: Minimal fungal inhibitory concentration
MLFC: Minimal lethal fungal concentration
MD: Molecular docking simulation
MD: Molecular dynamics simulation
NMR: Nuclear magnetic resonance
Pip: Piperidine
KOH: Potassium hydroxide
Rg: Radius of gyration
RMSD: Root mean square deviation
RMSF: Root mean square fluctuation
SASA: Solvent accessible surface area
Et3N: Triethylamine
WB: Water bath
