# Supplementary material for: Antifungal activity and biocompatibility assessment with molecular docking and dynamic simulations of new pyrazole derivatives
Source: BMC Biotechnol. 2025 Feb 6;25:15. doi: 10.1186/s12896-025-00948-8 (PMC11800497; doi:10.1186/s12896-025-00948-8)
Supplement: Supplementary file 1 — Supplementary Material 1 [file 12896_2025_948_MOESM1_ESM.pdf]

Antifungal activity and biocompatibility assessment with molecular docking of new pyrazole derivatives

Basma T. Abd-Elhalim[1*], Ghada G. El-Bana[2,3], Ahmed F. El-Sayed[4,5], Ghada E. Abdel-Ghani[2]
[1] Department of Agricultural Microbiology, Faculty of Agriculture, Ain Shams University, PO Box 68-Hadayek Shoubra, Cairo, 11241, Egypt.
[2]Department of Chemistry, Faculty of Science, Mansoura University, El-Gomhoria Street, Mansoura ET- 35516, Egypt.
[3]Mansoura University Student's Hospital, Mansoura University, El-Gomhoria Street, Mansoura ET-35516, Egypt.
[4]Microbial Genetics Department, Biotechnology Research Institute, National Research Centre, Giza, Dokki, 12622, Egypt.
[5]Egypt Center for Research and Regenerative Medicine (ECRRM), Cairo, Egypt.

Experimental

General Remarks:

The melting point of the synthesized compounds was measured in degrees Celsius (°C) using the Gallenkamp apparatus. The IR spectra were obtained using FTIR spectrometers, Thermo Scientific Nicolet iS10, Mattson 5000 at Mansoura University. The NMR analysis for the synthesized compounds was performed on a Bruker 400 MHz and JEOL 500 MHz, Mansoura University in DMSO-d6 and D2O as the solvent with tetramethylsilane used as an internal reference. The EI-Mass spectra were analyzed in a Thermo scientific GCMS model ISQ LT at the Regional Center for Mycology and Biotechnology (RCMB) at Al-Azhar University, Nasr City, Cairo. Elemental analysis (C, H, and N) was performed at Cairo University. The insecticidal activity was performed at the insect lab of the Plant Protection Research Institute, Mansoura. TLC Silica gel 60 F254, Merck, Germany.

3.2 Chemical Reactions:

General procedure of preparation of 2-cyano-3-(1,3-diphenyl-1H-pyrazol-4-yl)-N-Substituted-acrylamide (3a-h).

A solution containing 1,3-diphenyl-1*H*-pyrazole-4-carbaldehyde (**1**) (0.01 mol) or 2-((1,3-diphenyl-1*H*-pyrazol-4-yl)methylene)malononitrile (**2**) (0.01 mol) and *N*-aryl cyanoacetamide (0.01 mol) in ethanol (20 ml) was refluxed with a catalytic amount of piperidine (2 drops) for 2 h. The resulting precipitate was filtered and washed with ethanol, then recrystallized from DMF: EtOH (1:2) to obtain the pure compounds **3a-h** in good yield.

2-cyano-3-(1,3-diphenyl-1*H*-pyrazol-4-yl)-*N*-(4-methylphenyl)acrylamide (3a).

Pale yellow sheet; yield 90%; m.p. >300 °C. IR (KBr): (ν cm^{-1}): 3363 (NH), 2205 (CN), 1682 (C=O), 1591 (C=N). ^{1}H NMR (DMSO-d_6) δ/ppm: 2.28(s, 3H, CH_3), 7.15-7.99 (m, 14 H, Ar-H), 8.14 (s, 1H, =CH), 9.21 (s, 1H, pyrazole-H), 10.24 (s, 1H, NH). ^{13}C NMR (DMSO-d_6) δ/ppm: 20.97, 105.74, 114.98, 117.24, 120.08 (2C), 121.38 (2C), 128.44, 129.38 (2C), 129.51 (5C), 129.68, 129.72, 130.37 (2C), 133.85, 136.24, 139.12, 142.18, 155.02, 160.44. MS: (m/z, %)= 404.65 (M^+, 13.08); Anal. Calcd for $C_{26}H_{20}N_4O$ (404.46): C, 77.21; H, 4.98; N, 13.85 %. Found C, 77.19; H, 4.96; N, 13.87 %

2-cyano-3-(1,3-diphenyl-1*H*-pyrazol-4-yl)-*N*-(4-methoxyphenyl)acrylamide (3b).

Yellow sheet; yield 87%; m.p. 250-252 °C. IR (KBr): (ν cm^{-1}): 3351 (NH), 2213 (CN), 1678 (C=O), 1596 (C=N). ^{1}H NMR (DMSO-d_6) δ/ppm: 3.75 (s, 3H, OCH_3), 6.83-7.98 (m, 14 H, Ar-H), 8.15 (s, 1H, =CH), 9.23 (s, 1H, pyrazole-H), 10.19 (s, 1H, NH). ^{13}C NMR (DMSO-d_6) δ/ppm: 55.67, 105.99, 114.26, 114.98, 117.24, 120.09 (2C), 123.02 (2C), 128.46, 129.39 (2C), 129.53 (4C), 129.69, 129.74, 130.38 (2C), 131.41, 131.63, 139.12, 142.13, 155.49, 160.22. MS: (m/z, %)= 420.03 (M^+, 21.6); Anal. Calcd for $C_{26}H_{20}N_4O_2$ (420.46): C, 74.27; H, 4.79; N, 13.33%. Found C, 74.29; H, 4.77; N, 13.31%

***N*-(4-bromophenyl)-2-cyano-3-(1,3-diphenyl-1*H*-pyrazol-4-yl)acrylamide (3c).**

Off white sheet; yield 89 %; m.p. 262-264 °C. IR (KBr): (ν cm^{-1}): 3360 (NH), 2204 (CN), 1683 (C=O), 1589 (C=N). ^{1}H NMR (DMSO-d_6) δ/ppm: 7.46-7.97 (m, 14 H, Ar-H), 8.16 (s, 1H, =CH), 9.22 (s, 1H, pyrazole-H), 10.41 (s, 1H, NH). ^{13}C NMR (DMSO-d_6) δ/ppm: 105.35, 114.92, 116.60, 117.11, 120.11 (2C), 123.26 (2C), 128.49, 129.40 (2C), 129.76 (4C), 130.37 (2C), 131.35, 131.98 (2C), 138.17, 139.09, 142.69, 155.11, 160.76.MS: (m/z, %)= 469.66 (M^+, 37.29); Anal. Calcd for $C_{25}H_{17}BrN_4O$ (469.33): C, 63.98; H, 3.65; N, 11.94%. Found C, 63.96; H, 3.67; N, 11.96%

***N*-(4-chlorophenyl)-2-cyano-3-(1,3-diphenyl-1*H*-pyrazol-4-yl)acrylamide (3d).**

Pale yellow sheet; yield 88%; m.p. 275-277 °C. IR (KBr): (ν cm^{-1}): 3358 (NH), 2204 (CN), 1684 (C=O), 1589 (C=N). ^{1}H NMR (DMSO-d_6) δ/ppm: 7.41-7.98 (m, 14 H, Ar-H), 8.17 (s, 1H, =CH), 9.23 (s, 1H, pyrazole-H), 10.44 (s, 1H, NH). ^{13}C NMR (DMSO-d_6) δ/ppm: 105.29, 114.91, 117.12, 120.12 (2C), 122.89 (2C), 128.49, 129.09 (2C), 129.42 (4C), 129.54, 129.78, 129.82, 130.39, 131.34 (2C), 137.70, 139.09, 142.71, 155.12, 160.77. MS: (m/z, %)= 424.66/ 426.68 (M^+/ M^++2, 38.59/ 59.55); Anal. Calcd for $C_{25}H_{17}ClN_4O$ (424.88): C, 70.67; H, 4.03; N, 13.19% Found C, 70.69; H, 4.05; N, 13.21%

4-(2-cyano-3-(1,3-diphenyl-1*H*-pyrazol-4-yl)acrylamido) benzoic acid (3e).

Pale yellow sheet; yield 89 %; m.p. > 300 °C. IR (KBr): (ν cm^{-1}): 3451 (OH), 3234 (NH), 2217 (CN), 1685 (C=O), 1565 (C=N). ^{1}H NMR (DMSO-d_6) δ/ppm: 7.46-7.96 (M, 14 H, Ar-H), 7.98 (s, 1H, =CH), 8.20 (s, 1H, pyrazole-H), 9.25 (s, 1H, NH), 10.61 (s, 1H, OH). ^{13}C NMR (DMSO-d_6) δ/ppm: 105.37, 114.89, 117.11, 120.11 (2C), 120.43 (2C), 127.07, 128.51, 129.42 (2C), 129.54 (2C), 129.78, 129.86, 130.38 (2C), 130.66 (2C), 131.33, 139.08, 142.67, 142.87, 155.14, 161.07, 167.46. MS: (m/z, %)= 434.85 (M^+, 10.76); Anal. Calcd for $C_{26}H_{18}N_4O_3$ (434.45): C, 71.88; H, 4.18; N, 12.90%. Found C, 71.87; H, 4.20; N, 12.92%

2-cyano-3-(1,3-diphenyl-1*H*-pyrazol-4-yl)-*N*-(4-nitrophenyl)acrylamide (3f).

Yellow sheet; yield 87%; m.p. 268-270 °C. IR (KBr): (ν cm^{-1}): 3330 (NH), 2207 (CN), 1687 (C=O), 1585 (C=N). ^{1}H NMR (DMSO-d_6) δ/ppm: 7.47-8.23 (m, 14H, Ar-H), 8.27 (s, 1H, =CH), 9.25 (s, 1H, pyrazole-H), 10.85 (s, 1H, NH). ^{13}C NMR (DMSO-d_6) δ/ppm: 114.90, 117.07, 120.15 (2C), 120.97 (2C), 125.22 (2C), 128.55, 129.44 (2C), 129.54 (4C), 129.81, 129.95, 130.39 (2C), 131.31, 131.31, 139.08, 143.27, 155.22, 161.58. MS: (m/z, %) = 435.40 (M^+, 16.64); Anal. Calcd for $C_{25}H_{17}N_5O_3$ (435.43): C, 68.96; H, 3.94; N, 16.08%. Found C, 68.94; H, 3.97; N, 16.10%

2-cyano-3-(1,3-diphenyl-1*H*-pyrazol-4-yl)-*N*-(thiazol-2-yl)acrylamide (3g).

Yellow sheet; yield 86%; m.p. 280-282 °C. IR (KBr): (ν cm^{-1}): 3326 (NH), 2216 (CN), 1614 (C=O), 1566 (C=N). ^{1}H NMR (DMSO-d_6) δ/ppm: 7.20-7.96 (M, 12 H, Ar-H), 8.28 (s, 1H, =CH), 9.22 (s, 1H, pyrazole-H), 13.24 (s, 1H, NH). ^{13}C NMR (DMSO-d_6) δ/ppm: 113.23, 115.25 (2C), 117.82, 120.09 (2C), 128.44, 129.47 (3C), 129.50 (3C), 129.66, 129.71, 130.37 (2C), 131.45, 142.45, 155.26 (2C). MS: (m/z, %)= 397.68 (M^+, 23.02); Anal. Calcd for $C_{22}H_{15}N_5OS$ (397.45): C, 66.48; H, 3.80; N, 17.62%. Found C, 66.46; H, 3.82; N, 17.60%

***N*-(benzo[d]thiazol-2-yl)-2-cyano-3-(1,3-diphenyl-1*H*-pyrazol-4-yl)acrylamide (3h).**

Pale yellow sheet; yield 87 %; m.p. > 300 °C. IR (KBr): (ν cm^{-1}): 3340 (NH), 2205 (CN), 1689 (C=O), 1585 (C=N) cm^{-1}. Anal. Calcd for $C_{26}H_{17}N_5OS$ (447.52): C, 69.78; H, 3.83; N, 15.65%. Found C, 69.77; H, 3.81; N, 15.67%

4,5,6,7-tetrabromo-2-((1,3-diphenyl-1*H*-pyrazol-4-yl)methylene)amino)-isoindoline-1,3-dione (6).

A solution containing 4-(hydrazineylidenemethyl)-1,3-diphenyl-1*H*-pyrazole (**4**) (0.26 g, 0.001 mol) and 4,5,6,7-tetrabromoisobenzofuran-1,3-dione (**5**) (0.45 g, 0.001 mol) in AcOH (30 ml) was refluxed and allowed to form a precipitate after 3 hours. The solid was then collected by filtration and purified by recrystallization from DMF: EtOH (1:2) to yield pure compound **6**.

Pale yellow sheet; yield 75 %; m.p. > 300 °C.IR (KBr): (ν cm−1): 1754 (C=O), 1723 (C=O), 1589 (C=N) cm-1. MS: (m/z, %)= (708.11/ 710.89, (M+, 38.06/12.58); Anal. Calcd for C24H12Br4N4O2 (707.99): C, 40.72; H, 1.71; N, 7.91; %. Found C, 40.70; H, 1.73; N, 7.93%

5-(1,3-diphenyl-1*H*-pyrazol-4-yl)-3-oxo-2,3-dihydro-1*H*-pyrazole-4-carbonitrile (7).

Method a

Ethanolic solution of 1,3-diphenyl-1*H*-pyrazole-4-carbaldehyde (**1**) (2.5 g, 0.01mol) and 2-cyanoacetohydrazide (1 g, 0.01 mol) in the presence of the catalytic amount of base and or acid in EtOH was refluxed for 2 h. The solid was then collected through filtration and purified by recrystallization from DMF: EtOH (1:2) to give compound **7**.

Method b

Ethanolic solution of acetohydrazide **8** (3 g, 0.01mol) in the presence of the catalytic amount of Et_3N or AcOH was refluxed for 15 min. The solid was then collected through filtration and purified by recrystallization from DMF: EtOH (1:2) to give compound **7**.

Yellow sheet; yield 65 %; m.p. 250-252 °C. IR (KBr): (ν cm^{-1}): 3422 (NH), 2205 (CN), 1687 (C=O amide), 1586 (C=N). ^{1}H NMR (DMSO-d6) δ/ppm: 7.34–8.23 (m, 10H, Ar-H), 8.58 (s, 1H, NHNHCO), 9.22 (s, 1H, OH), 9.28, 9.29 (two, s, 1H, pyrazole-H), 11.86 (br, 1H, NHCO). Anal. Calcd for $C_{19}H_{13}N_5O$ (327.35): C, 69.71; H, 4.00; N, 21.39 %. Found C, 69.69; H, 3.98; N, 21.37 %.

2-cyano-*N'*-((1,3-diphenyl-1*H*-pyrazol-4-yl)methylene)acetohydrazide (8).

A solution of 1,3-diphenyl-1*H*-pyrazole-4-carbaldehyde (**1**) (2.5 g, 0.01 mol) and 2-cyanoacetohydrazide (1 g, 0.01 mol) in EtOH (20 ml) was heated on a water bath, which formed a crude precipitate after 0.5 hr. The solid that was produced was gathered by filtering it, and it was purified by recrystallization from AcOEt, resulting in the isolation of pure compound **8**.

White sheet; yield 92 %; m.p. 210-211 °C. IR (KBr): (ν cm^{-1}): 3184 (NH), 2258 (CN), 1678 (C=O amide), 1599 (C=N). ^{1}H NMR (DMSO-d_6) δ/ppm: 7.40–8.04 (m, 10H, Ar-H), 9.03 (s, 1H, pyrazole-H), 11.64 (s, 1H, NNHCO exchangeable); for anti- isomer: 4.13 (s, 2H, CH2), 8.13 (s, 1H, CH=N); for syn isomer: δ 3.79 (s, 2H, CH_2), 8.25 (s, 1H, CH=N). ^{13}C NMR (DMSO-d_6) δ/ppm: 24.41, 116.59, 117.12, 119.11(2C), 127.53, 128.74, 128.89, 128.92, 129.11(2C), 130.14, 132.57, 137.84, 139.43, 151.75, 158.84, 164.76, 160.44. Anal. Calcd for $C_{19}H_{15}N_5O$ (329.36): C, 69.29; H, 4.59; N, 21.26%. Found C, 69.26; H, 4.57; N, 21.22%.

General procedure of preparation of *N'*-(1,3-diphenyl-1*H*-pyrazol-4-yl)methylene)-2-oxo-6-(p-substituted)-2*H*-chromene-3-carbohydrazide (10a-e).

A solution containing 2-cyano-*N'*-((1,3-diphenyl-1*H*-pyrazol-4-yl)methylene)-acetohydrazide (**8**) (0.33 g, 0.01 mol), and *p*-substituted salicylaldehyde **9a-e** (0.01 mol) in absolute ethanol (20 ml) along with a small quantity of piperidine (2-3 drops) was stirred at 50 0C for ½ hr. The resulting precipitate was filtered, washed with EtOH, dried, and recrystallized from EtOH: DMF (2:1) to yield the desired compounds **10a-e**.

***N'*-((1,3-diphenyl-1*H*-pyrazol-4-yl)methylene)-2-oxo-2*H*-chromene-3-carbohydrazide (10a).**

Yellow powder; yield 69%; m.p. 222-224 °C. IR (KBr): (ν cm^{-1}): 3313 (NH), 1682 (COO), 1638 (CONH), 1604 (C=N). ^{1}H NMR (DMSO-*d$_6$*): δ 7.25-8.04 (m, 18H, Ar-H), 8.54 (s, 1H, =CH), 8.37 (s, 1H, CH=N), 9.05 (s 1H, pyrazole-H), 13.46 (br s, 1H, NHCO). Anal. Calcd for $C_{26}H_{18}N_4O_3$ (434.46): C, 71.88; H, 4.18; N, 12.90 %. Found C, 71.86; H, 4.15; N, 12.92 %.

***N'*-((1,3-diphenyl-1*H*-pyrazol-4-yl)methylene)-2-oxo-6-(*p*-tolydiazenyl)-2*H*-chromene-3-carbo-hydrazide (10b).**

Red powder; yield 72%; m.p. 250-252 °C.IR (KBr): (ν cm^{-1}): 3451 (NH), 1680 (COO), 1599 (CONH), 1546 (C=N). ^{1}H NMR (DMSO-*d$_6$*) δ/ppm: 2.39 (s, 3H, CH_3), 7.38-8.03 (m, 17 H, Ar-H), 8.53 (s, 1H, =CH), 8.67 (s, 1H, CH=N), 9.03 (s, 1H, pyrazole-H), 11.82 (s, 1H, NHCO). MS: (m/z, %)= 552.03 (M^+, 16.11); Anal. Calcd for $C_{33}H_{24}N_6O_3$ (552.58): C, 71.73; H, 4.38; N, 15.21 %. Found C, 71.71; H, 4.36; N, 15.19%

6-((4-chlorophenyl)diazenyl)-*N'*-(1,3-diphenyl-1*H*-pyrazol-4-yl)methylene)-2-oxo-2*H*-chromene-3-carbohydrazide (10c).

Orange powder; yield 70 %; m.p. 222-224 °C.IR (KBr): (ν cm^{-1}): 3451 (NH), 1600 (COO), 1599 (CONH), 1546 (C=N). ^{1}H NMR (DMSO-*d$_6$*) δ/ppm: 7.35-7.95 (m, 17 H, Ar-H), 8.02 (s, 1H, =CH), 9.02 (s, 1H, CH=N), 9.03 (s, 1H, pyrazole-H), 11.78 (s, 1H, NHCO). MS: (m/z, %)= 572.35/ 575.61 (M^+-1/ M^+, 38.09/ 20.86); Anal. Calcd for $C_{32}H_{21}ClN_6O_3$ (573.00/ 575.14): C, 67.08; H, 3.69; N, 14.67%. Found C, 67.06; H, 3.67; N, 14.65%

***N'*-((1,3-diphenyl-1*H*-pyrazol-4-yl)methylene)-6-((4-nitrophenyl)diazenyl)-2-oxo-2*H*-chromene-3-carbohydrazide (10d).**

Brown powder; yield 71%; m.p. 200-202 °C. IR (KBr): (ν cm^{-1}): 3451 (NH), 1599 (COO), 1699 (CONH), 1547 (C=N). ^{1}H NMR (DMSO-*d$_6$*) δ/ppm: 7.36-8.01 (m, 17 H, Ar-H), 8.54 (s, 1H, =CH), 8.75 (s, 1H, CH=N), 9.01 (s, 1H, pyrazole-H), 11.80 (s, 1H, NHCO). MS: (m/z, %)= 582.66 (M^+-1, 19.02); Anal. Calcd for $C_{32}H_{21}N_7O_5$ (583.16): C, 65.86; H, 3.63; N, 16.80%. Found C, 65.84; H, 3.66; N, 16.78 %

4-((3-(2-(1,3-diphenyl-1*H*-pyrazol-4-yl)methylene)hydrazine-1-carbonyl)-2-oxo-2*H*-chromen-6-yl)diazenyl)benzoic acid (13e).

Orange powder; yield 70 %; m.p. 290-292 °C. IR (KBr): (ν cm^{-1}): 3419 (OH), 3318 (NH), 1713 (COOH), 1675 (COO), 1601 (CONH), 1548 (C=N). ^1H NMR (DMSO-d_6) δ/ppm: 7.35-8.14 (m, 17 H, Ar-H), 8.54 (s, 1H, =CH), 8.73 (s, 1H, CH=N), 9.01 (s, 1H, pyrazole-H), 11.72 (s, 1H, NH), 12.94 (s, 1H, OH). MS: (m/z, %)= 581.59/ 582.16 (M^+-1/ M^+, 25.86/ 25.70)/ 581.17; Anal. Calcd for $C_{33}H_{22}N_6O_5$ (582.58): C, 68.04; H, 3.81; N, 14.43%. Found C, 68.06; H, 3.83; N, 14.41%

2-cyano-3-(dimethylamino)-*N'*-((1,3-diphenyl-1*H*-pyrazol-4-yl)methylene)-acrylo-hydrazide (11).

A solution of acetohydrazide **8** (0.23 g, 0.01 mol) and *N*,*N*-dimethylformamide dimethyl acetal (2.7 ml, 0.02 mol) in toluene (15 mL) was heated under reflux for 1 h., resulting in the formation of a solid precipitate. The solid product was collected by filtration, washed with hot EtOH, and recrystallized from a mixture of EtOH: DMF (2:1) to obtain pure compound **11**.

Pale yellow sheet; yield 85 %; m.p. 237-239 °C. IR (KBr): (ν cm^{-1}): 3450 (NH), 2194 (CN), 1659 (CONH), 1604 (C=N). ^1H NMR (DMSO-d_6) δ/ppm: 3.23 (s, 3H, NCH_3), 3.30 (s, 3H, NCH_3), 7.36-8.01 (m, 11H, Ar-H, =CH-N$(Me)_2$), 8.46 (s, 1H, =CH), 8.89 (s, 1H, pyrazole-H), 10.86 (s, 1H, NH). ^{13}C NMR (DMSO-*d6*) δ/ppm: 38.66, 47.57, 69.28, 117.80 (2C), 119.21, 119.60 (2C), 127.22, 127.39, 128.81, 128.97, 129.22 (2C), 130.10 (2C), 132.58, 139.55 (2C), 151.98, 157.49 (2C).MS: (m/z, %)= 384.67 (M^+, 24.68); Anal. Calcd for $C_{22}H_{20}N_6O$ (384.17): C, 68.73; H, 5.24; N, 21.86 %. Found C, 68.71; H, 5.22; N, 21.84 %

4-((dimethylamino)methylene)-6-(1,3-diphenyl-1*H*-pyrazol-4-yl)pyridazine-3,5(4*H*,6*H*)-dione (12).

Compound **11** (0.4 gm, 0.001 mol) was heated under reflux in AcOH (10 ml) for 30 minutes. The formed crude solid was filtered and washed with hot ethanol, and pure compound **12** was obtained by recrystallization from a mixture of EtOH: Dioxane (2:1).

Red Powder; yield 83%; m.p. >300 °C. IR (KBr): (ν cm−1): 1685 (C=O), 1615 (C=N). MS: (m/z, %)= 385.79 (M^+, 79.03); Anal. Calcd for $C_{22}H_{19}N_5O_2$ (385.43): C, 68.56; H, 4.97; N, 18.17%. Found C, 68.54; H, 4.95; N, 18.15%.

2-cyano-3-(1,3-diphenyl-1*H*-pyrazol-4-yl)-*N*-(4-methylphenyl)acrylamide (3a)

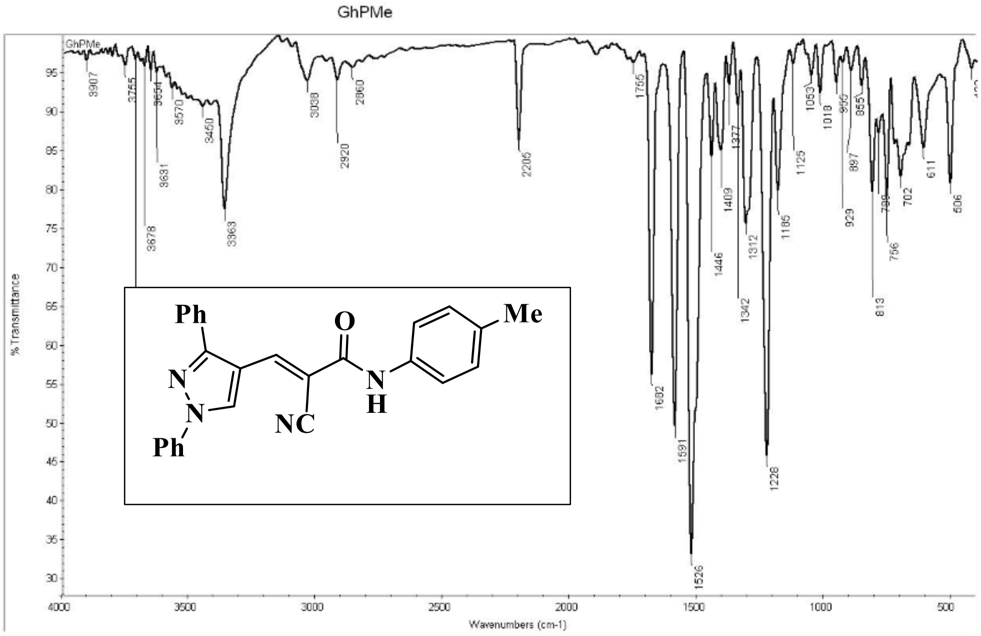

Figure S1: IR spectrum of compound 3a

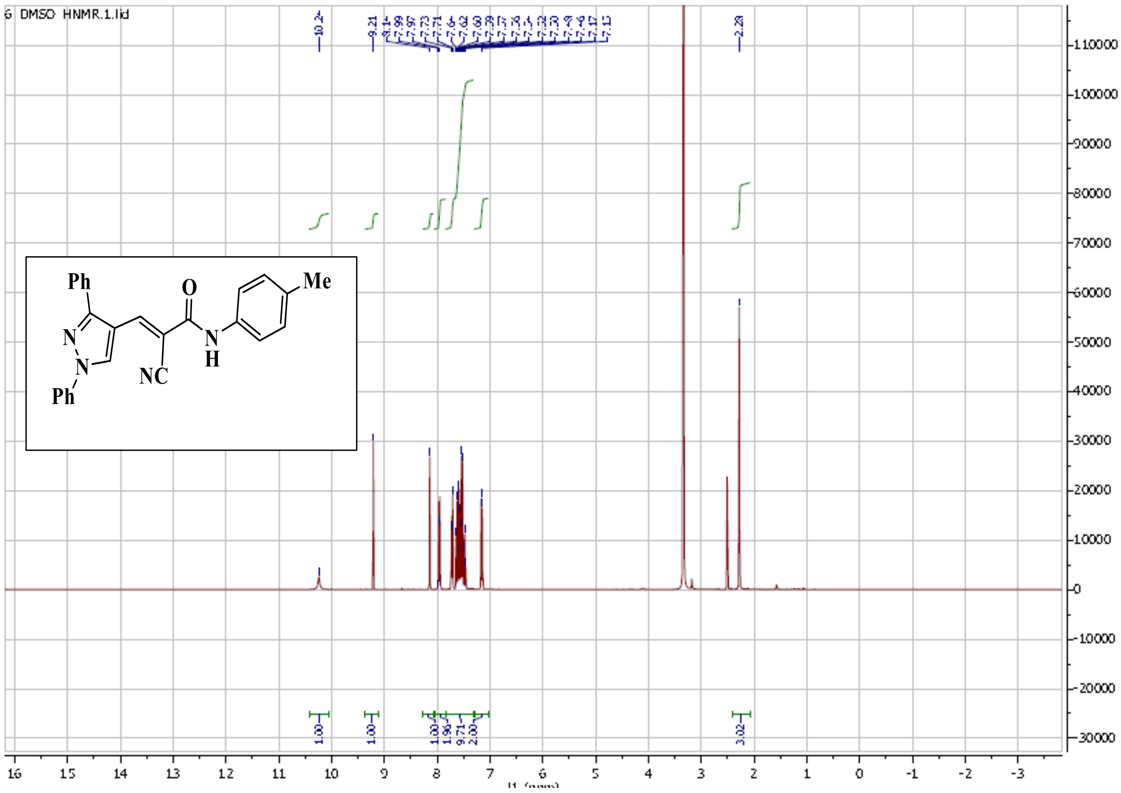

Figure S2: ^{1}H NMR spectrum of compound 3a

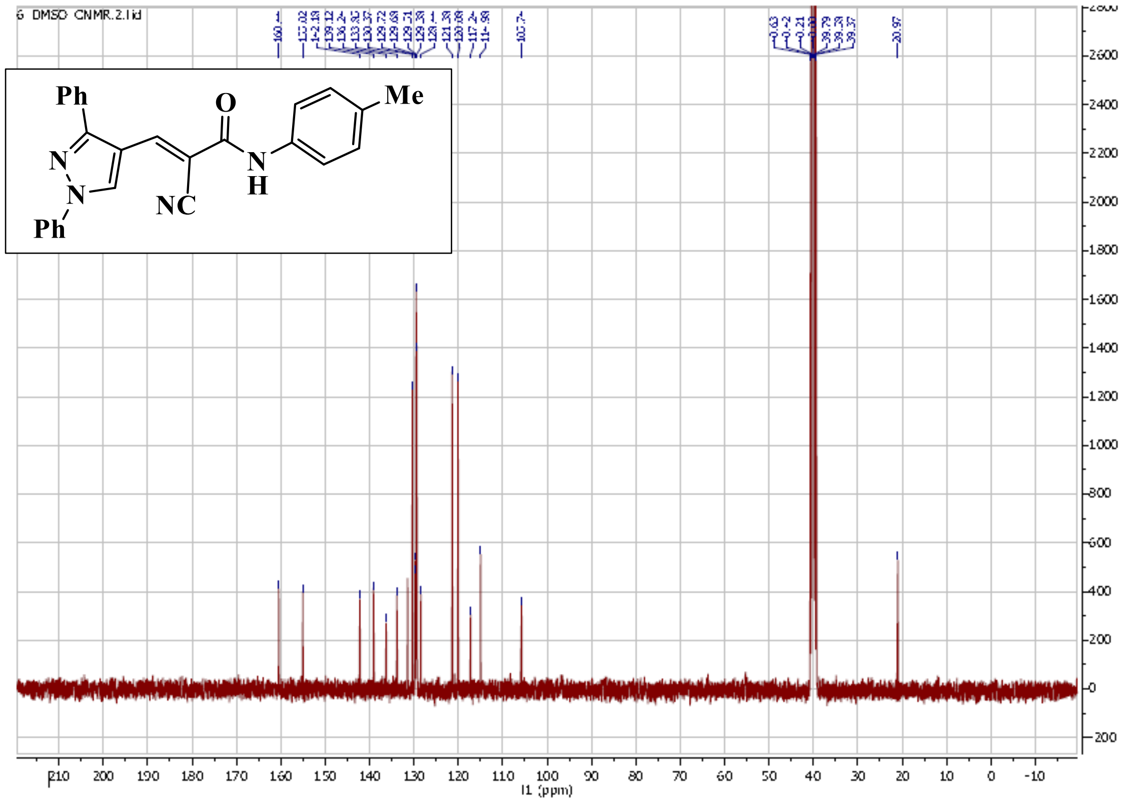

Figure S3: ^{13}C NMR spectrum of compound 3a

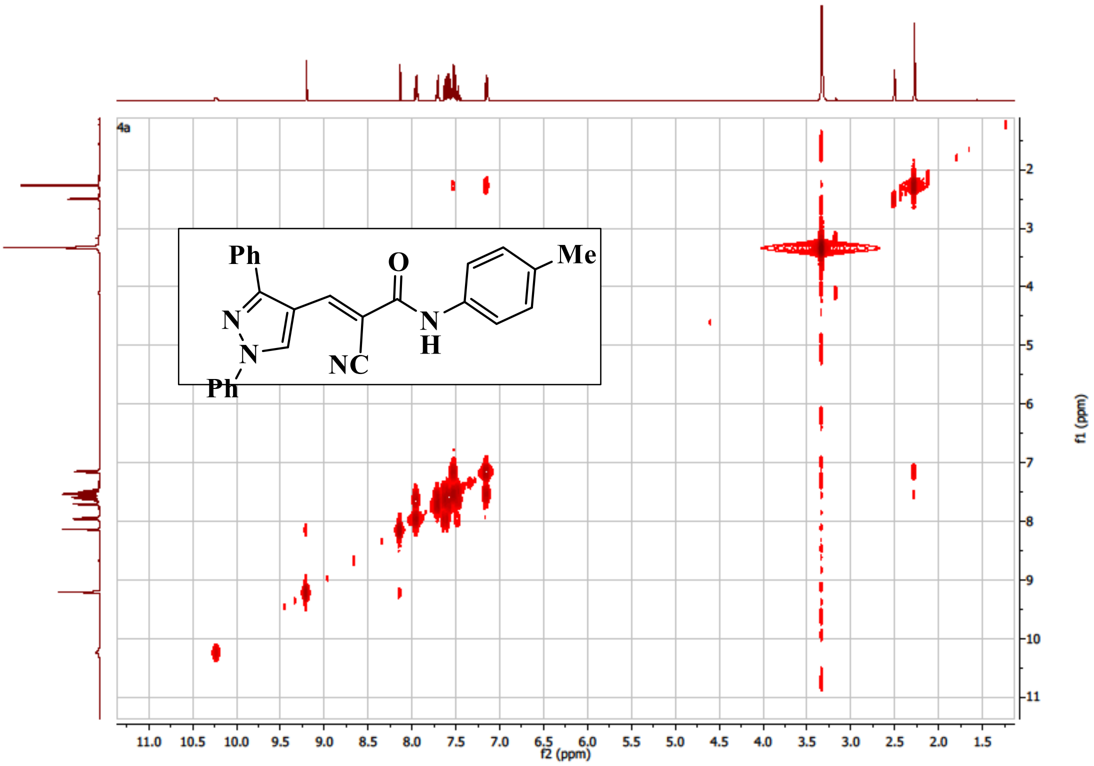

Figure S4: ^{1}H COSY spectrum of compound **3a**

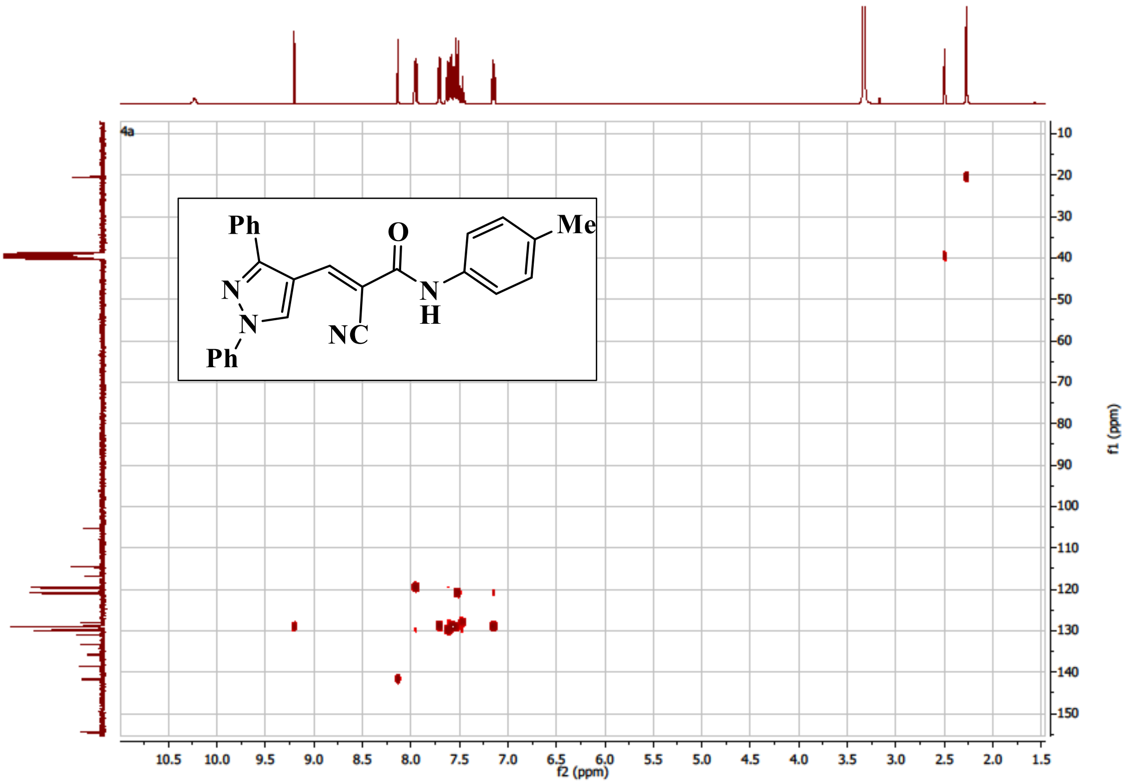

Figure S5: ^{1}H SQC spectrum of compound **3a**

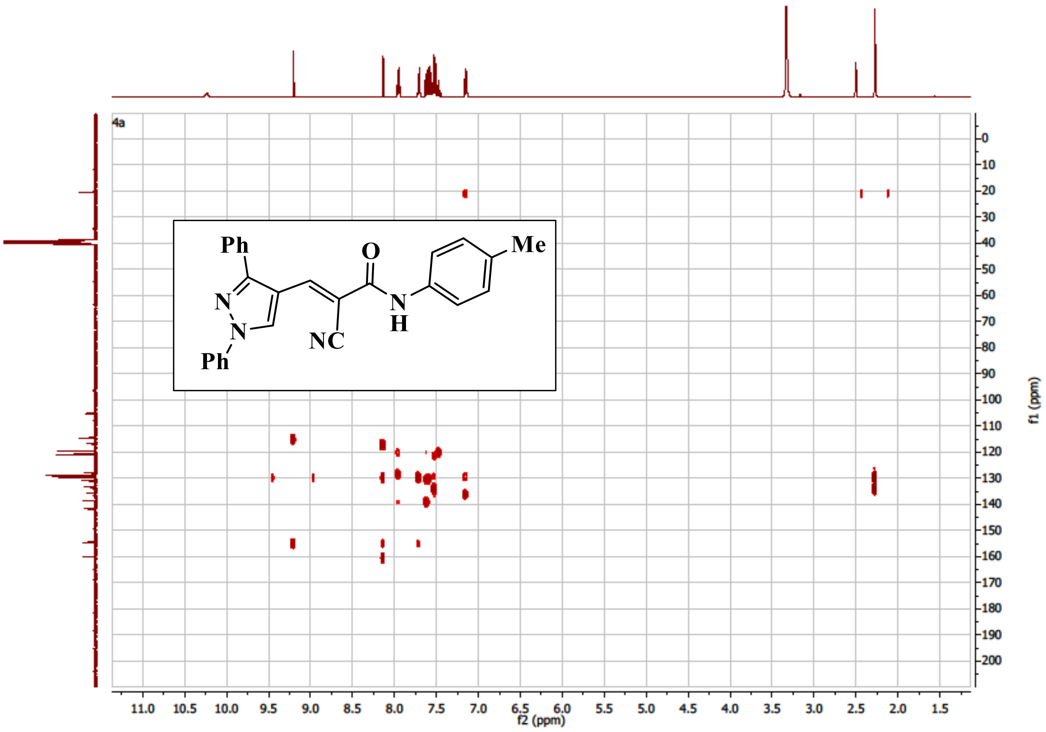

Figure S6: ^{1}H MBC spectrum of compound **3a**

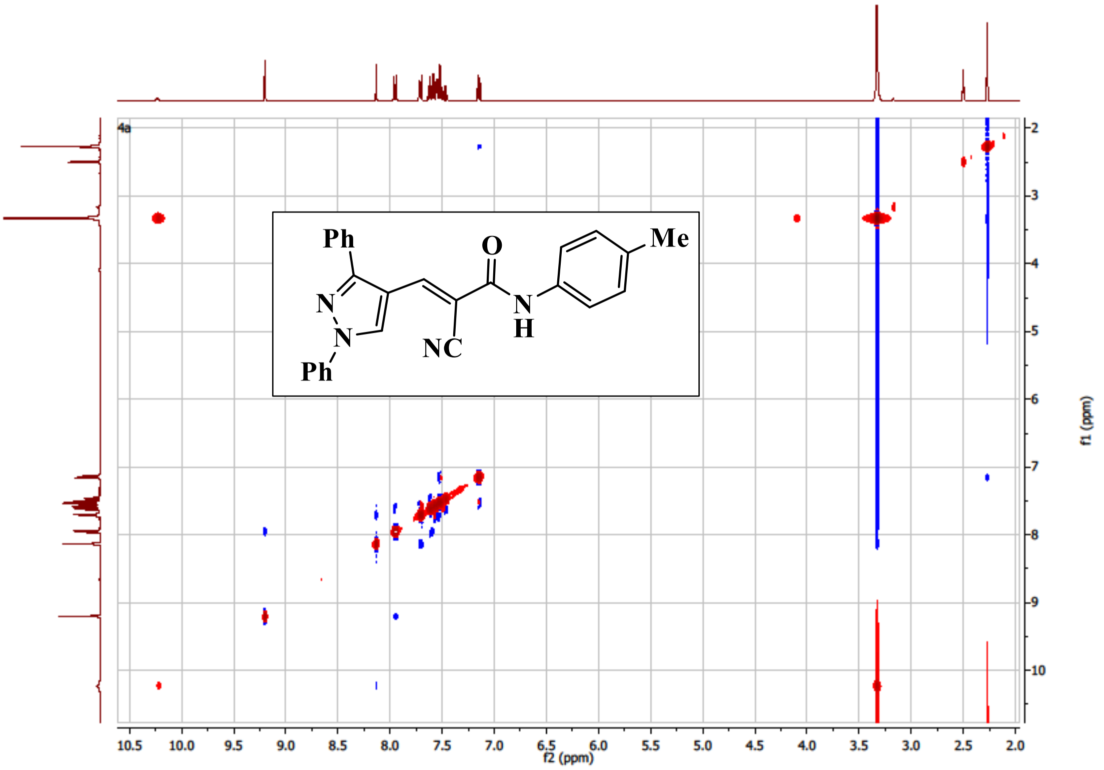

Figure S7: ^{1}H NOSY spectrum of compound **3a**

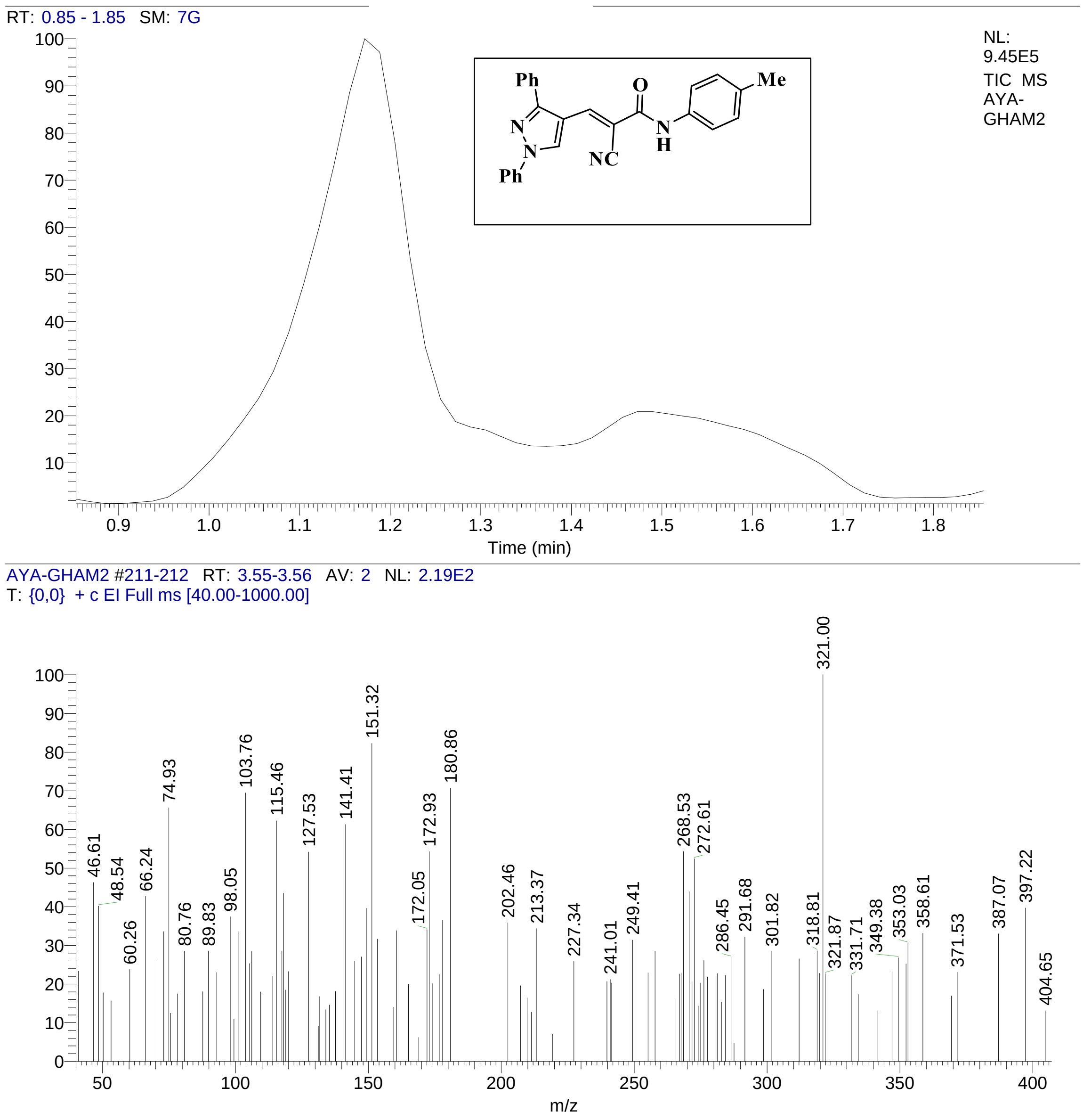

Figure S8: Mass spectrum of compound 3a

2-cyano-3-(1,3-diphenyl-1*H*-pyrazol-4-yl)-*N*-(4-methoxyphenyl)acrylamide (3b)

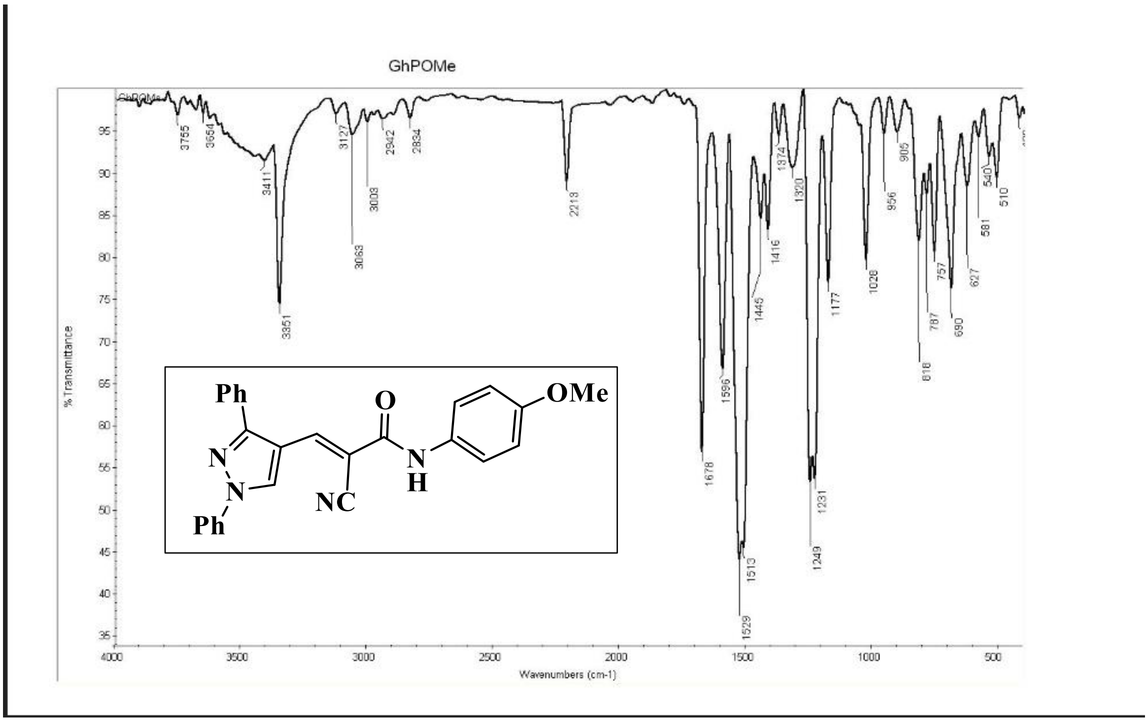

Figure S9: IR spectrum of compound 3b

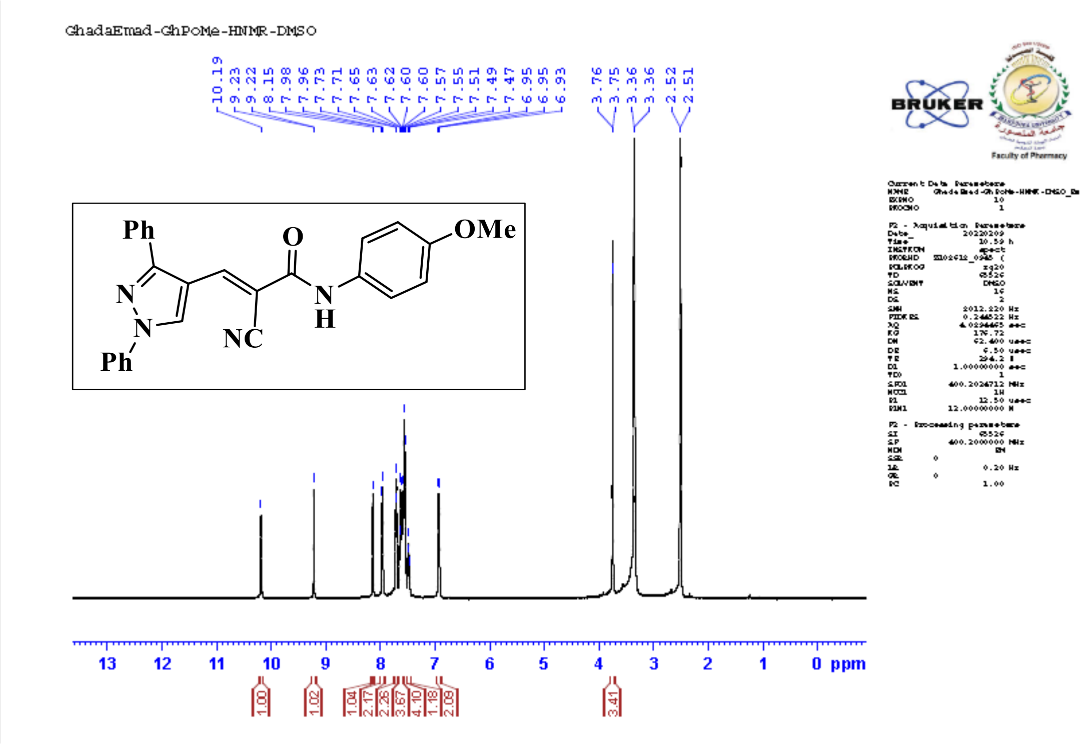

Figure S10: ^{1}H NMR spectrum of compound 3b

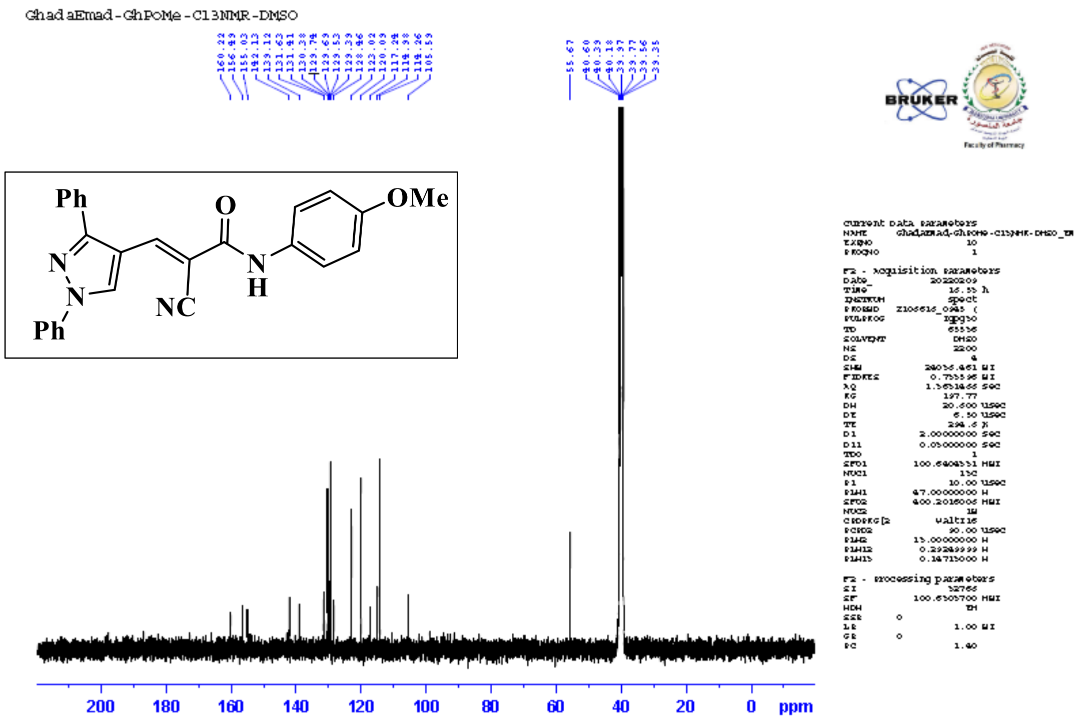

Figure S11: ^{13}C NMR spectrum of compound 3b

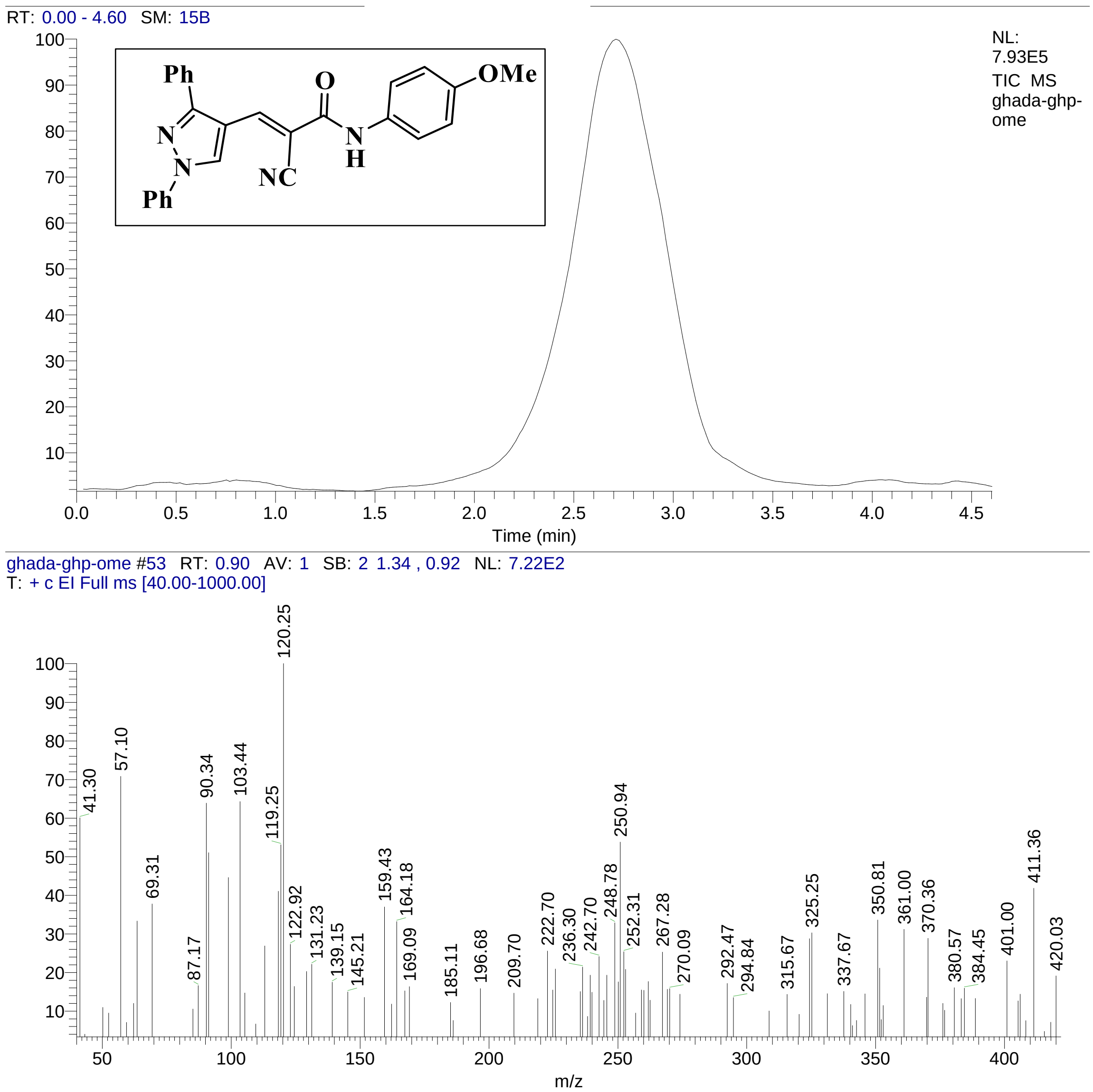

Figure S12: Mass spectrum of compound 3b

***N*-(4-bromophenyl)-2-cyano-3-(1,3-diphenyl-1*H*-pyrazol-4-yl)acrylamide (3c)**

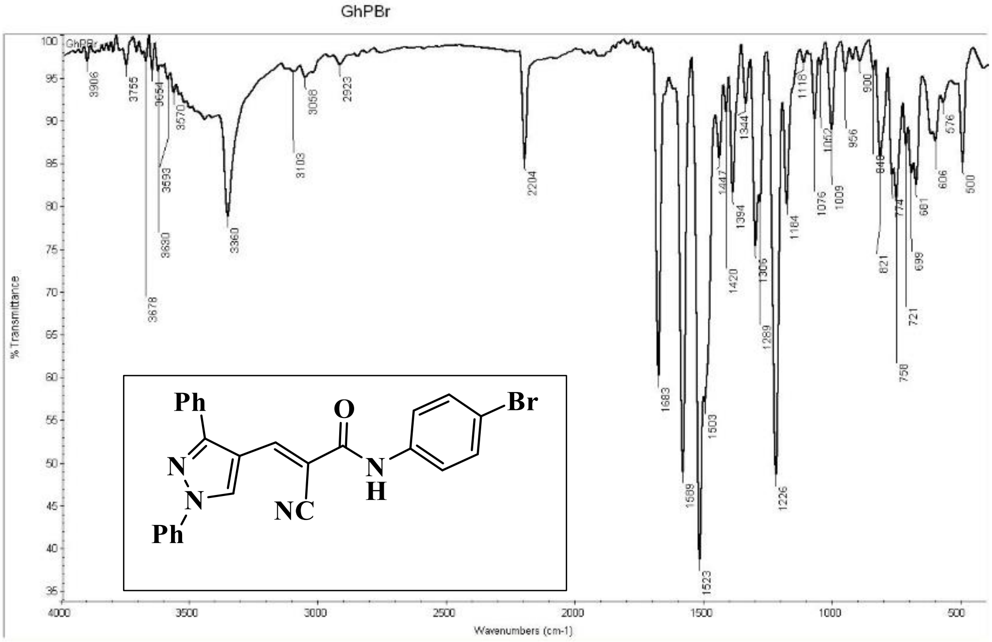

Figure S13: IR spectrum of compound 3c

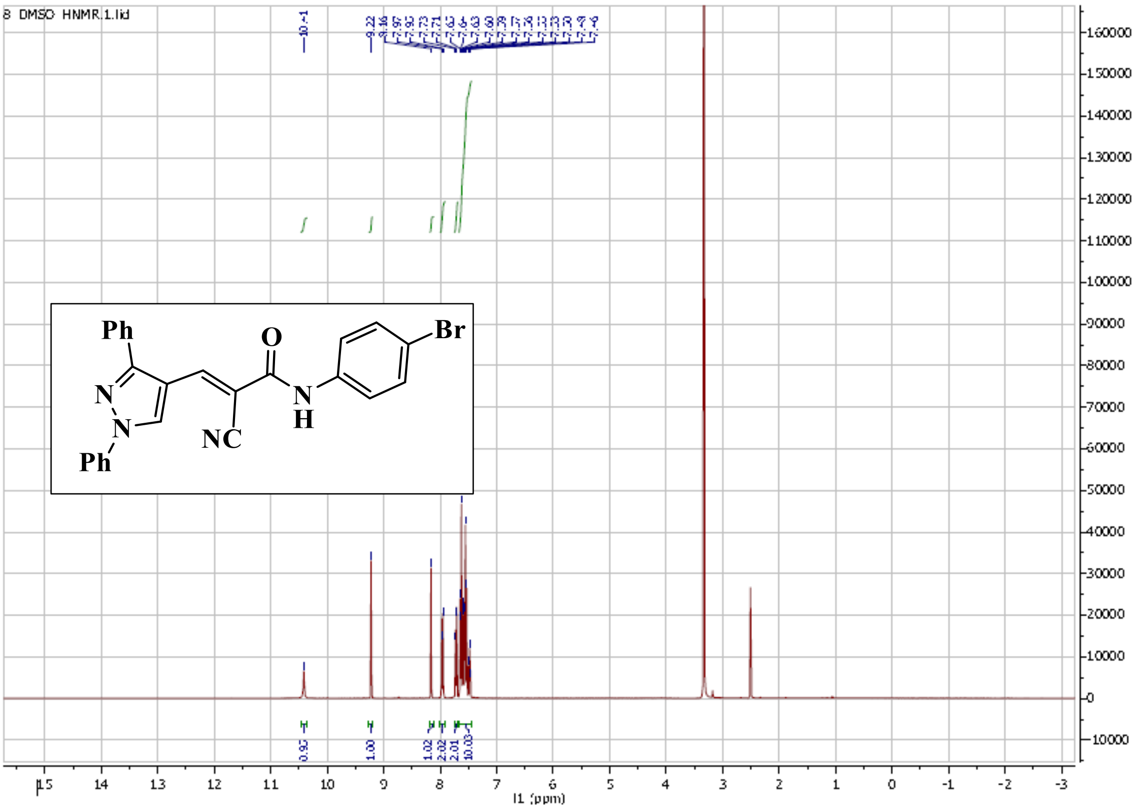

Figure S14: ^{1}H NMR spectrum of compound 3c

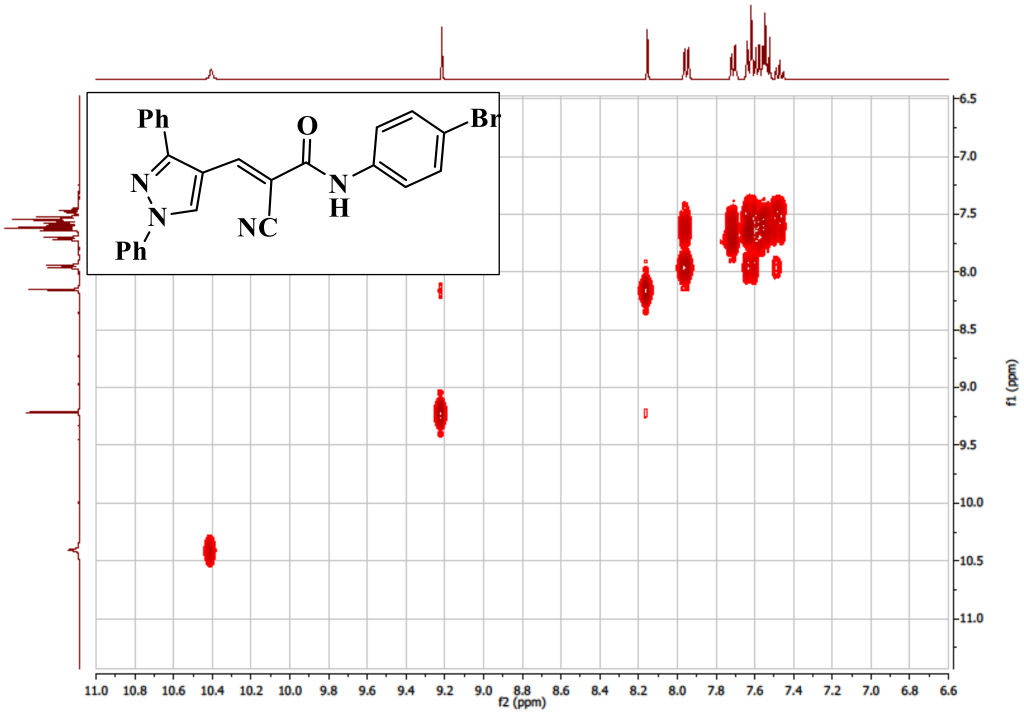

Figure S15: ^{1}H COSY spectrum of compound **3c**

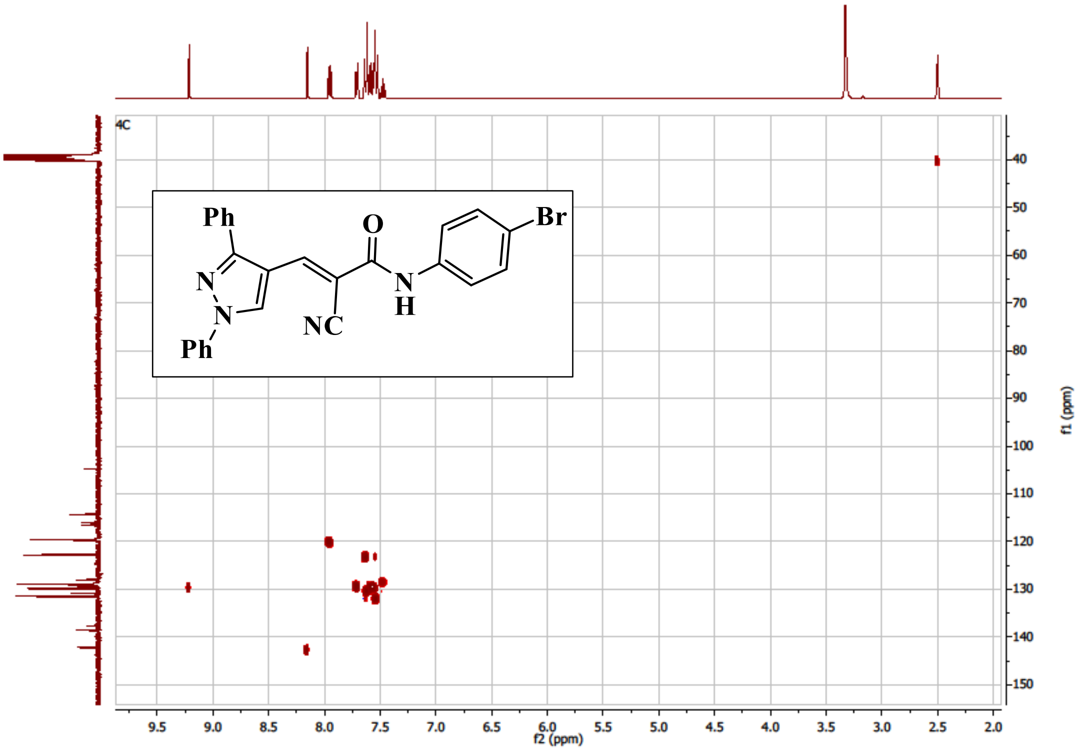

Figure S16: [1]HSQC spectrum of compound **3c**

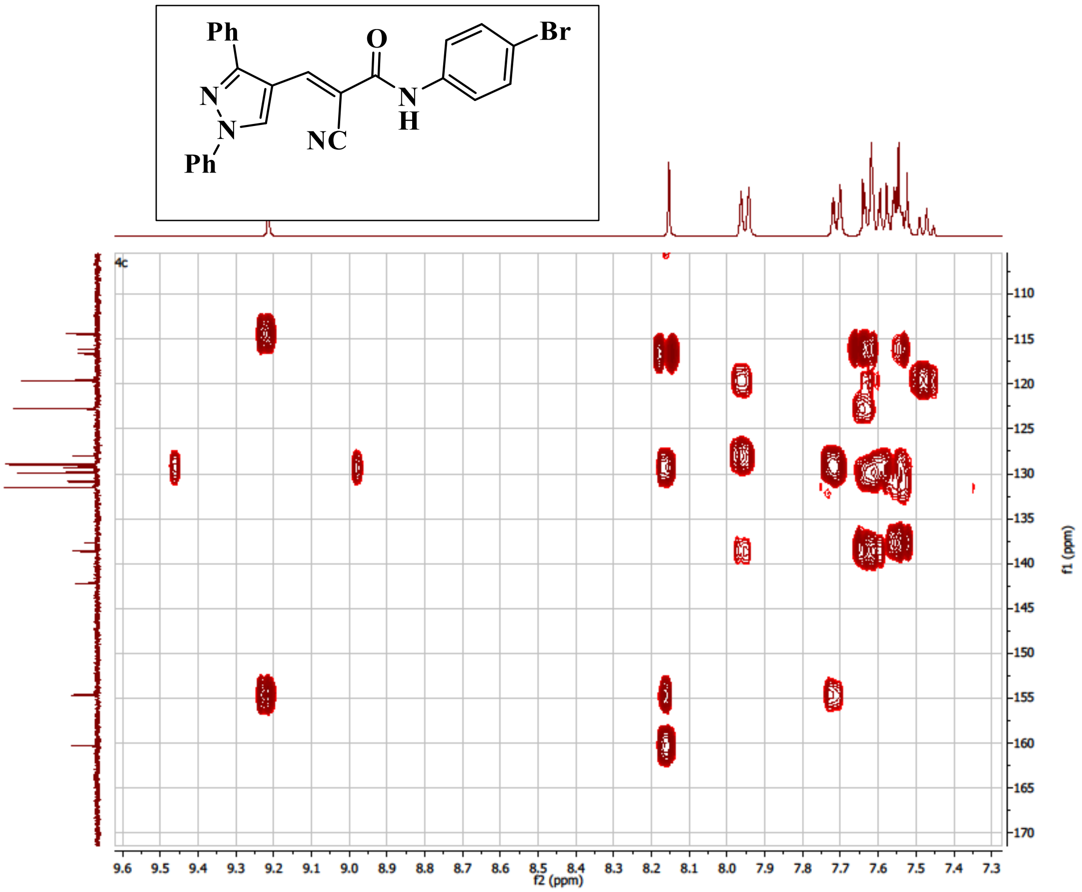

Figure S17: ^{1}H MBC spectrum of compound **3c**

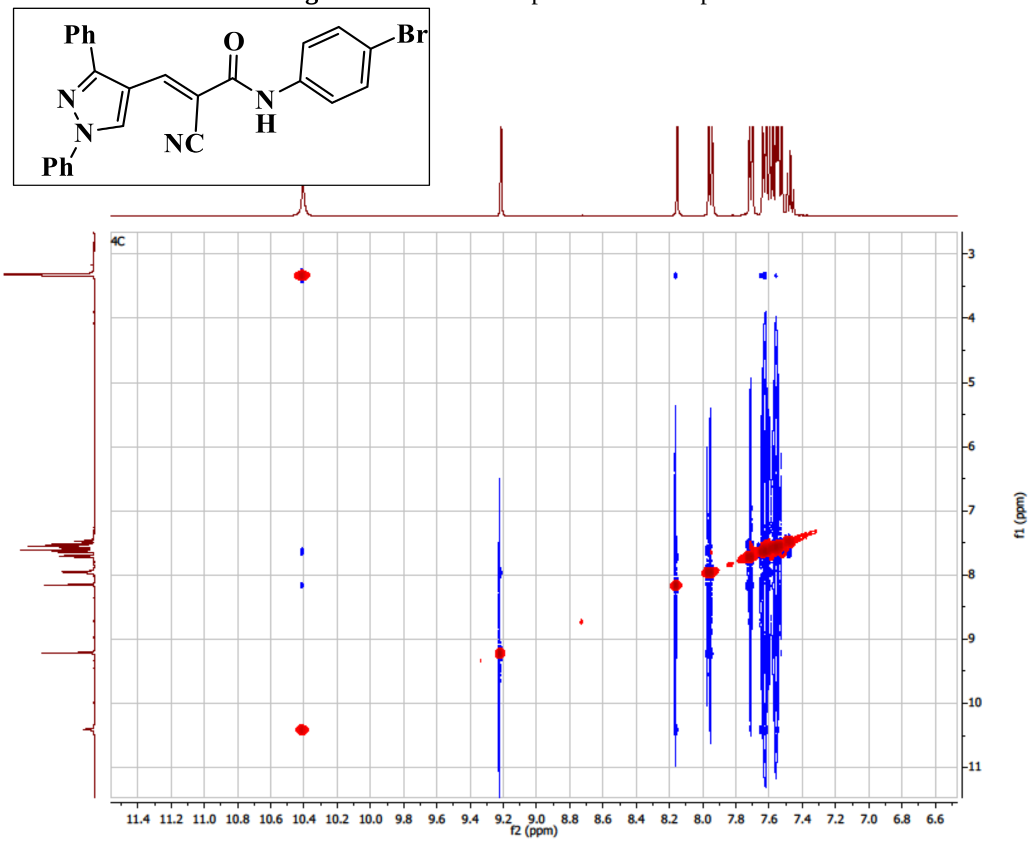

Figure S18: ^{1}H NOSEY spectrum of compound **3c**

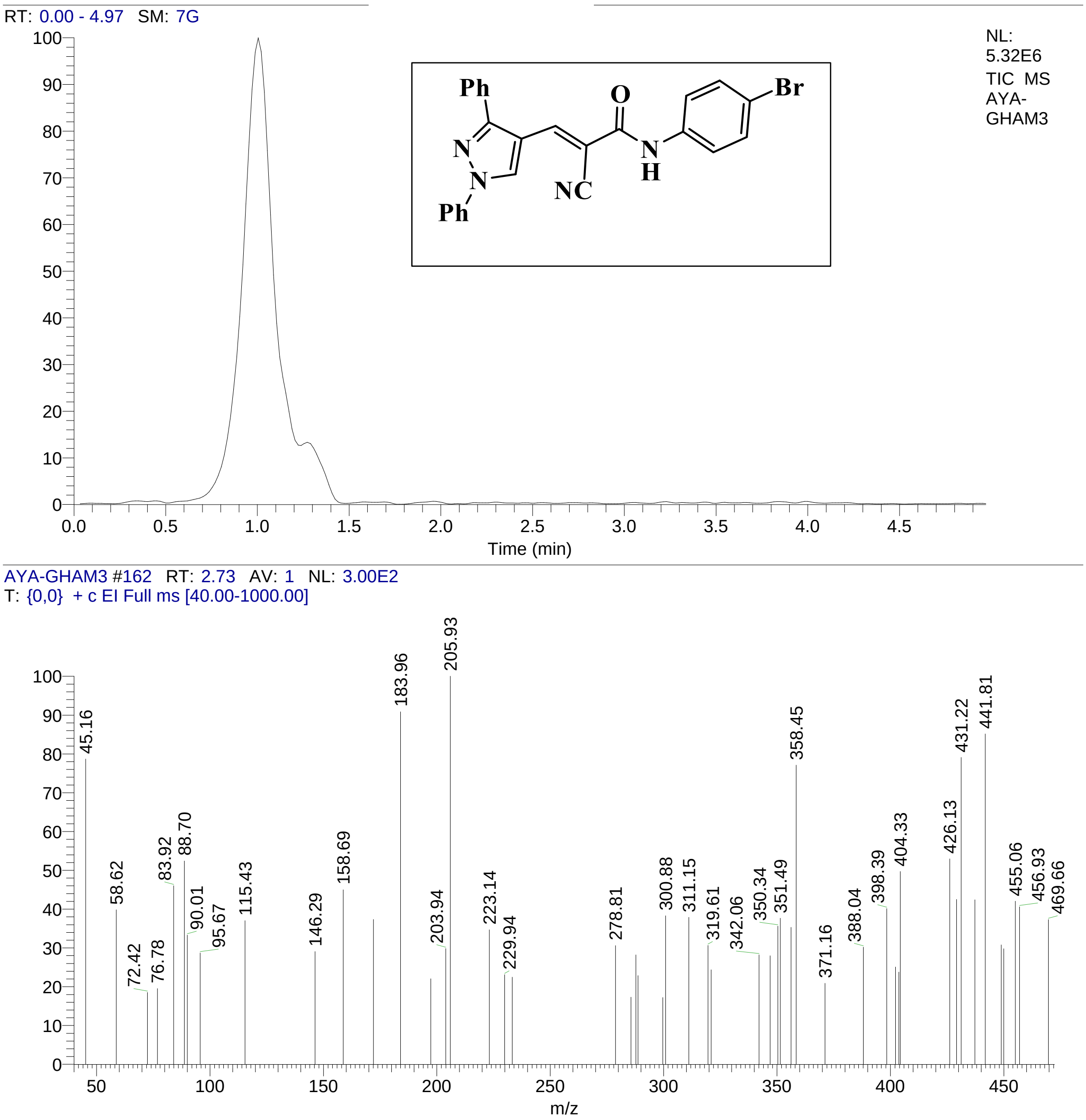

Figure S19: Mass spectrum of compound 3c

***N*-(4-chlorophenyl)-2-cyano-3-(1,3-diphenyl-1*H*-pyrazol-4-yl)acrylamide (3d):**

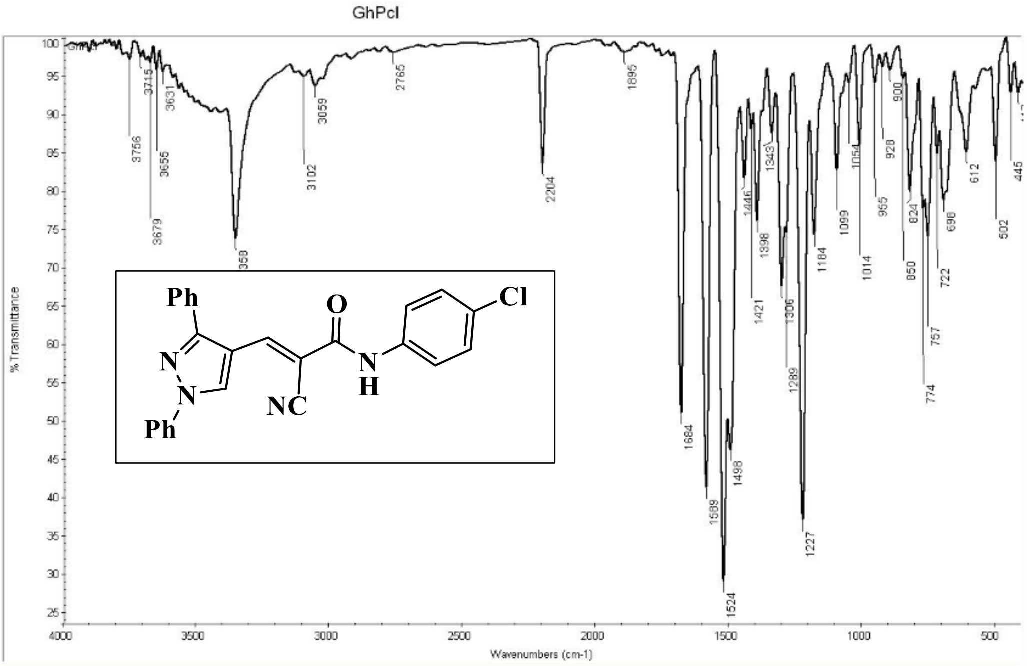

Figure S20: IR spectrum of compound 3d

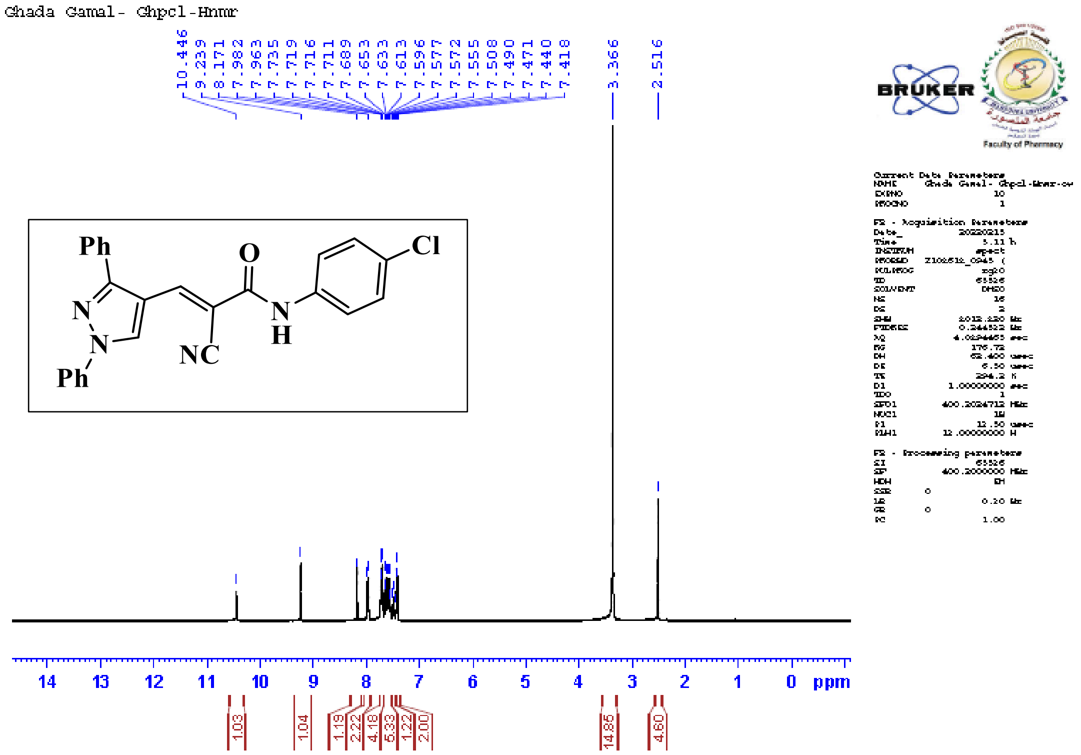

Figure S21: ^{1}H NMR spectrum of compound 3d

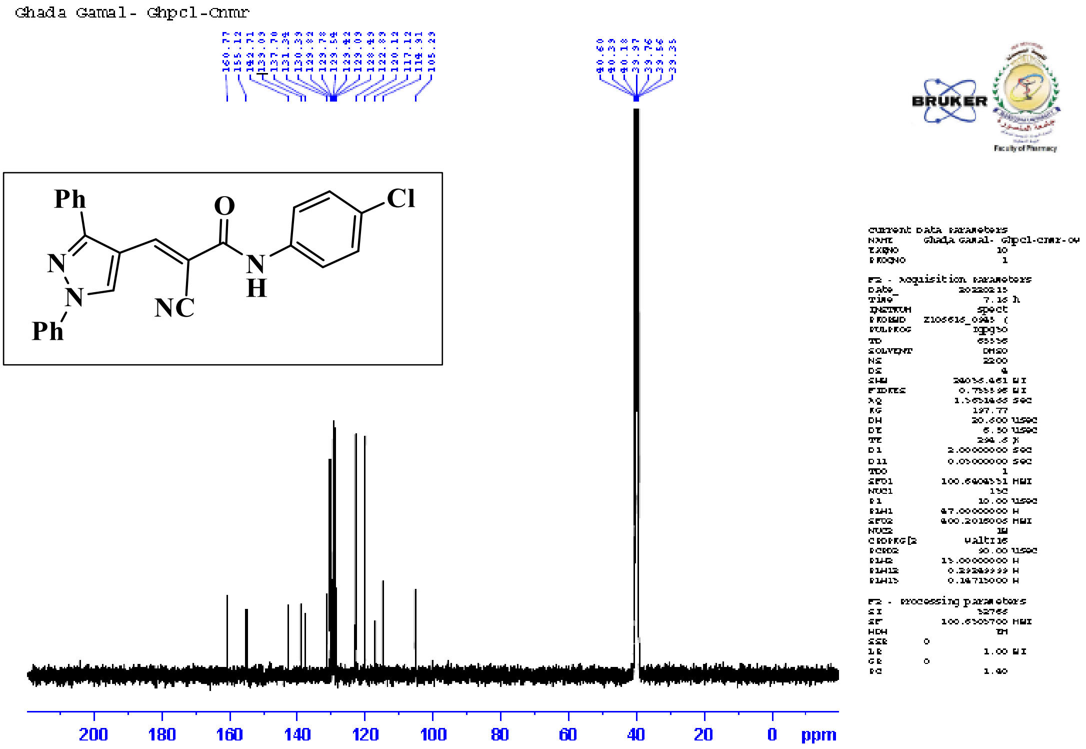

Figure S22: ^{13}C NMR spectrum of compound 3d

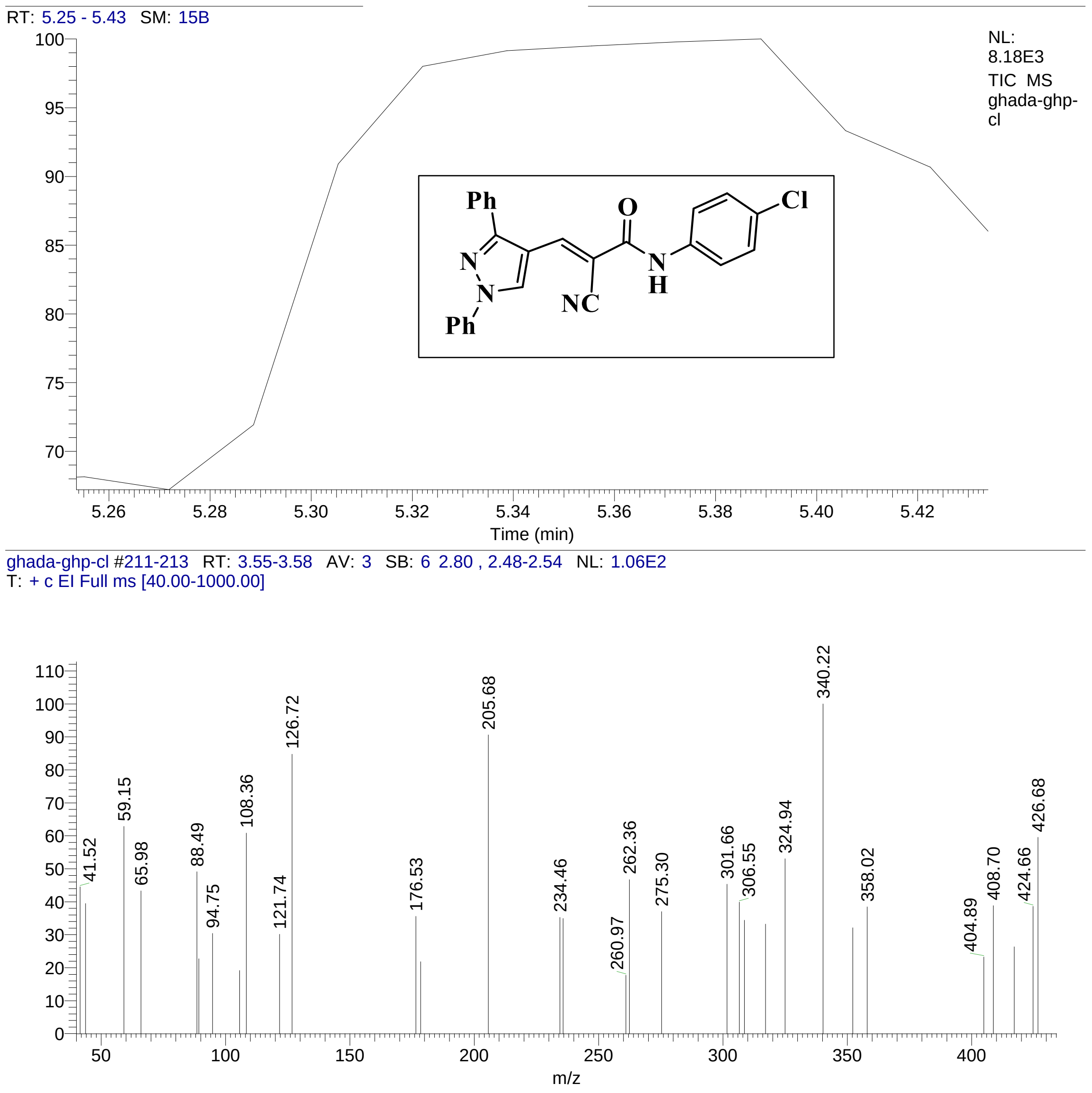

Figure S23: Mass spectrum of compound 3d

4-(2-cyano-3-(1,3-diphenyl-1*H*-pyrazol-4-yl)acrylamido)benzoic acid (3e)

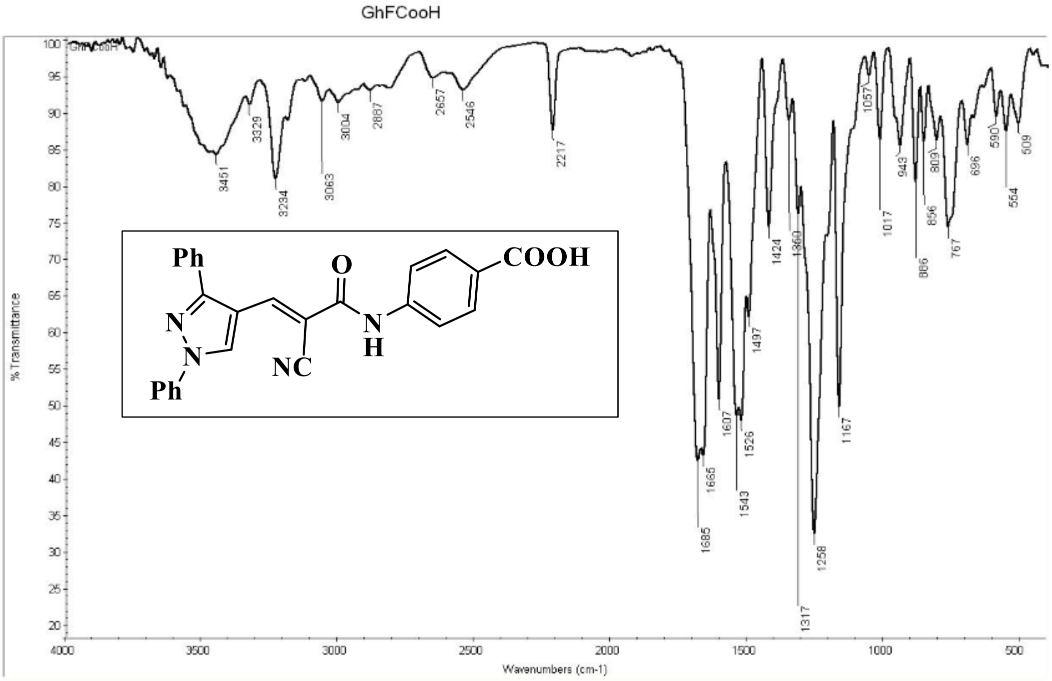

Figure S24: IR spectrum of compound 3e

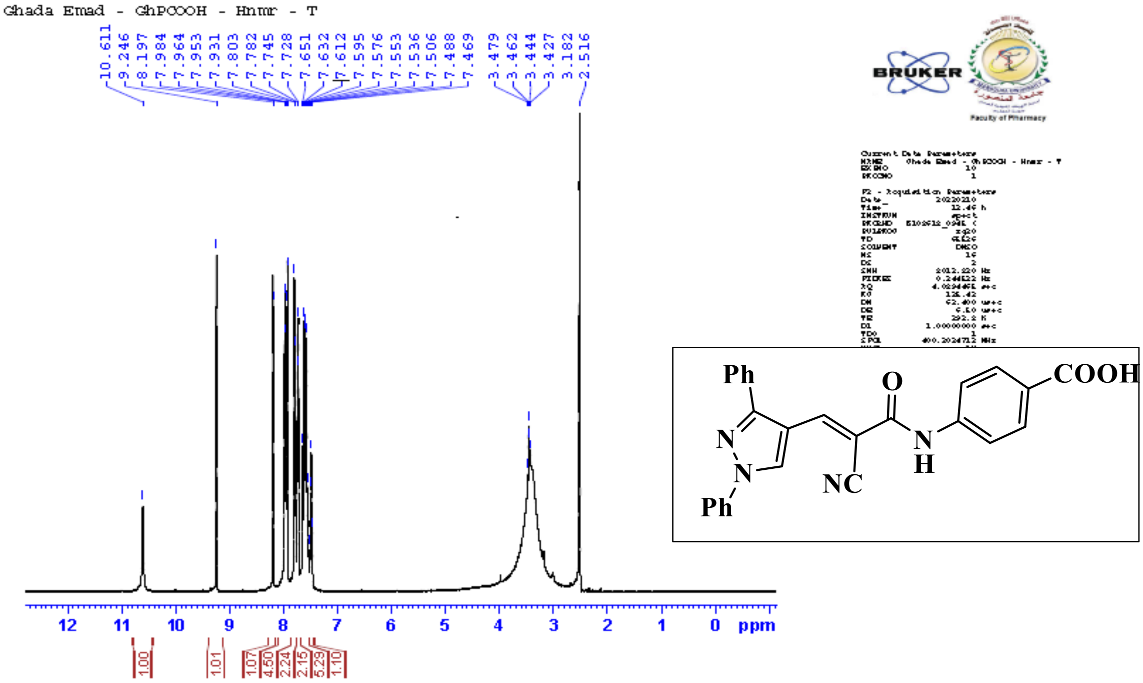

Figure S25: ^{1}H NMR spectrum of compound 3e

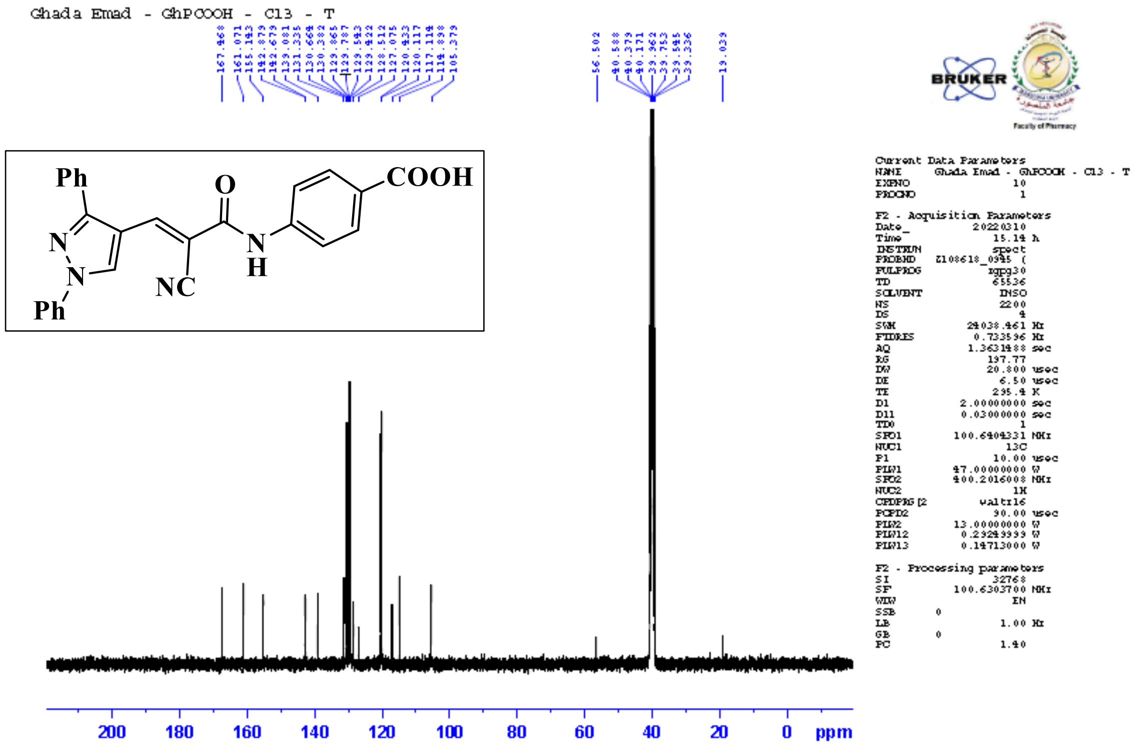

Figure S26: ^{13}C NMR spectrum of compound 3e

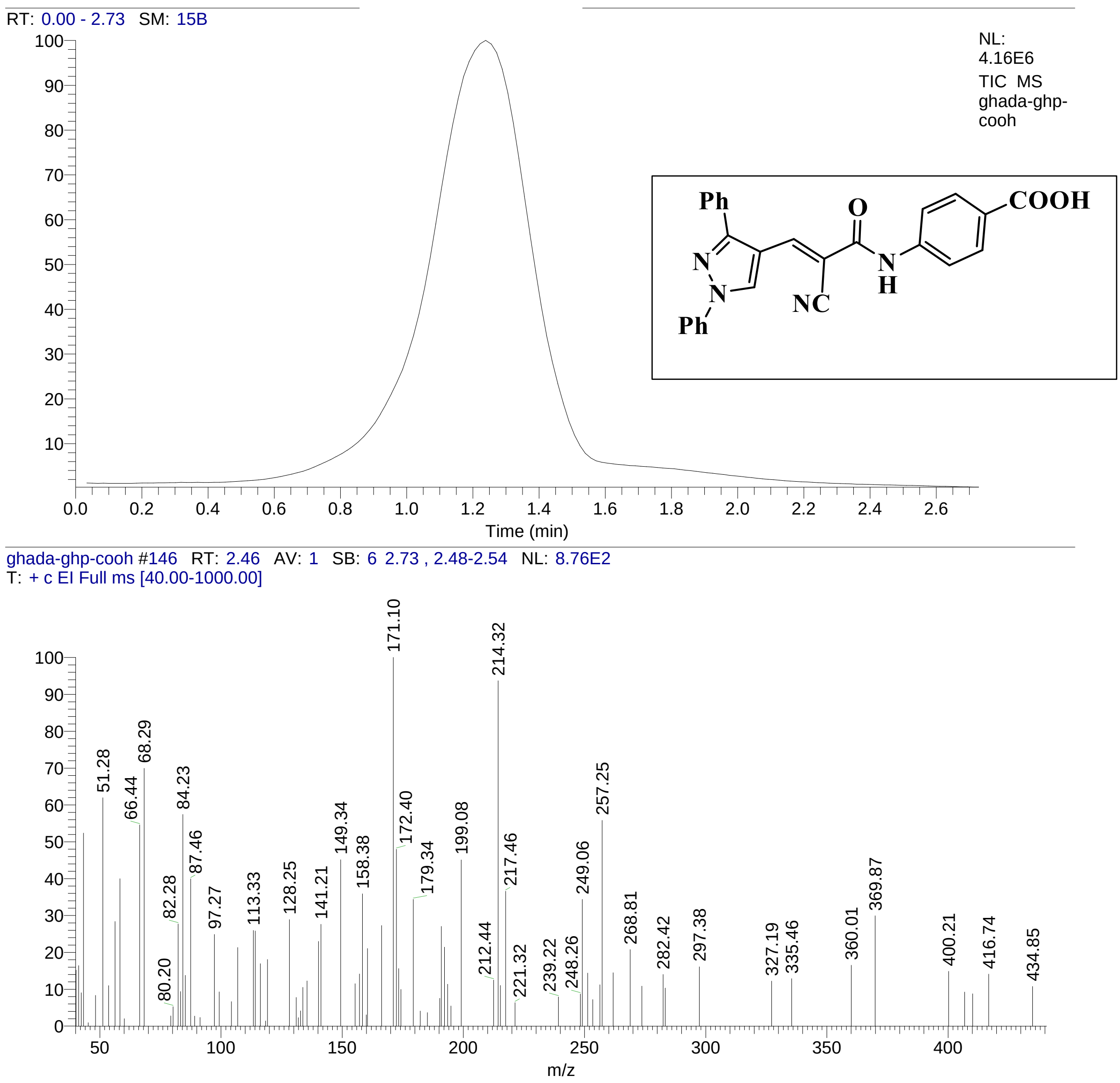

Figure S27: Mass spectrum of compound 3e

2-cyano-3-(1,3-diphenyl-1*H*-pyrazol-4-yl)-*N*-(4-nitrophenyl)acrylamide (3f)

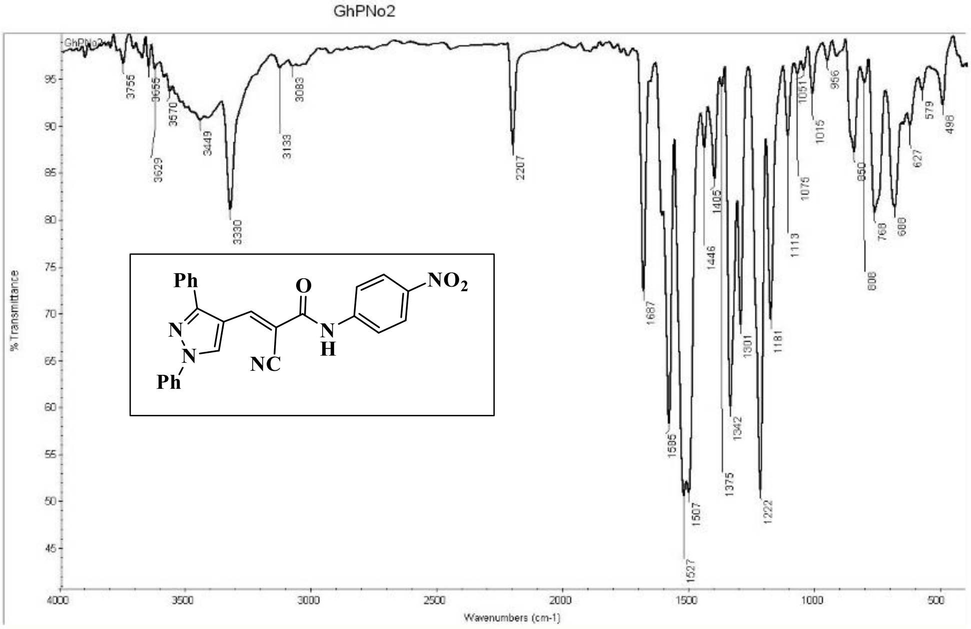

Figure S28: IR spectrum of compound 3f

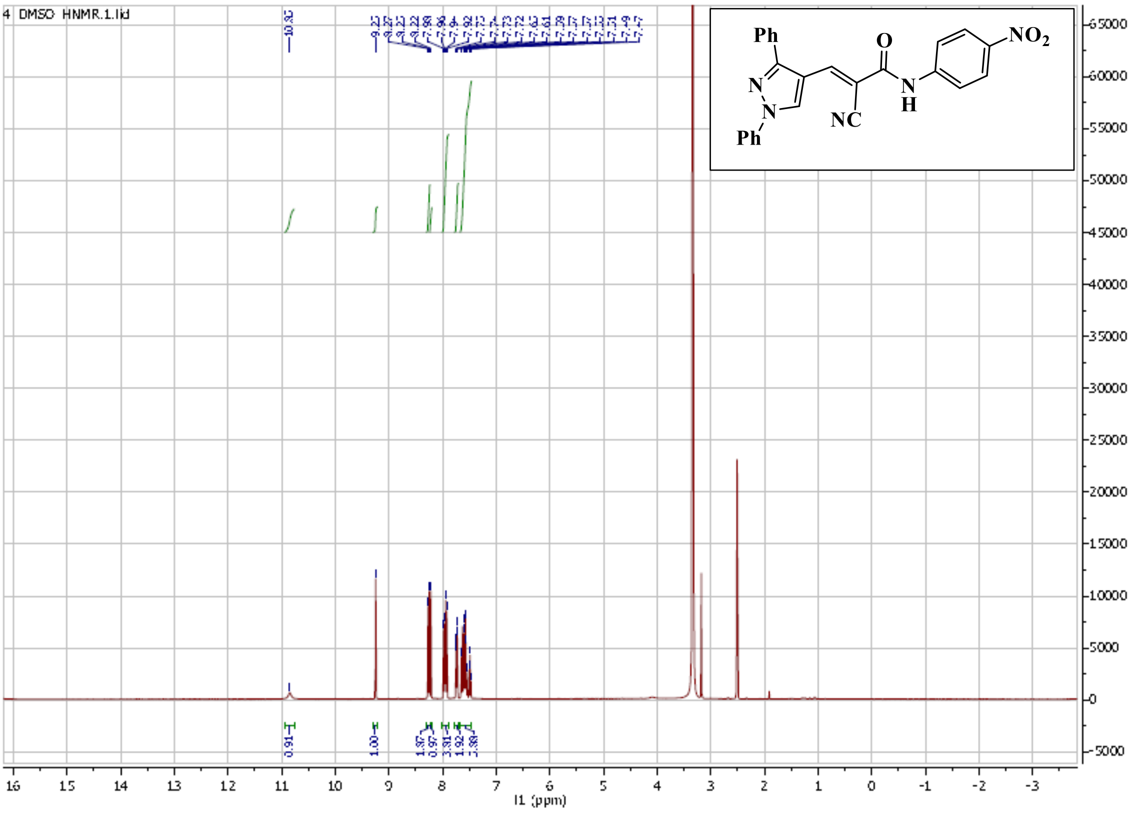

Figure S29: ^{1}H NMR spectrum of compound 3f

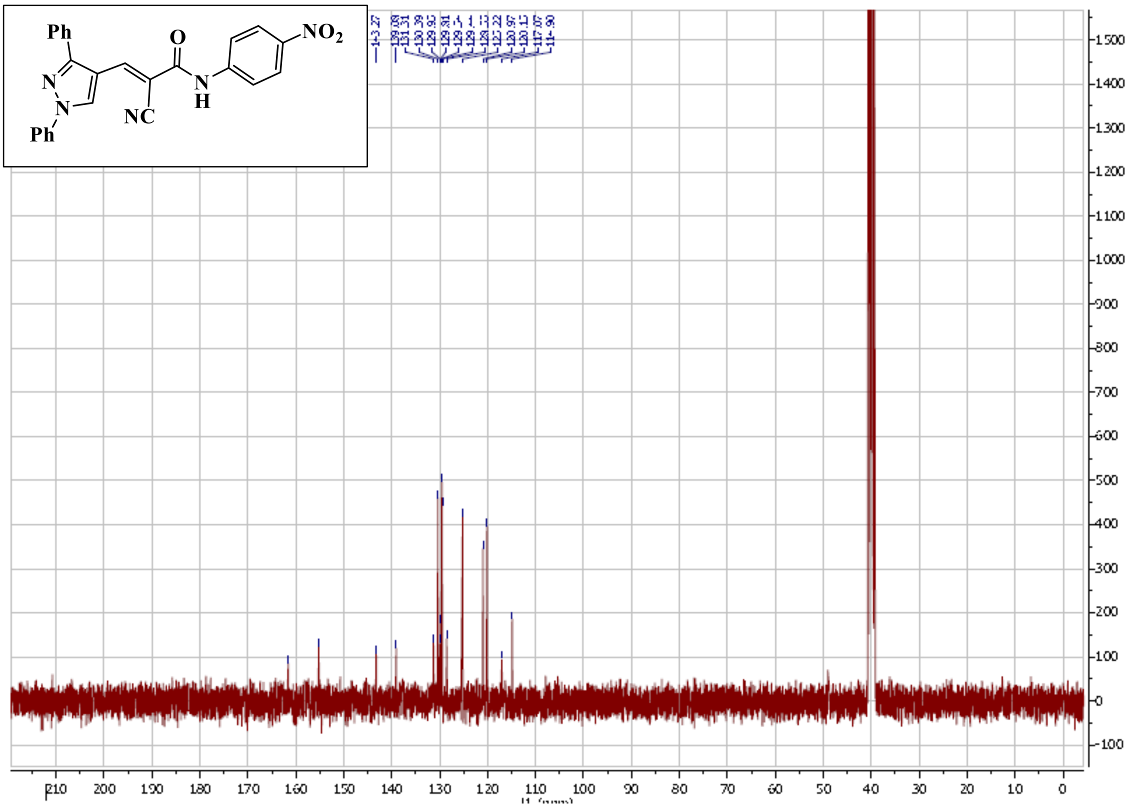

Figure S30: ^{13}C NMR spectrum of compound 3f

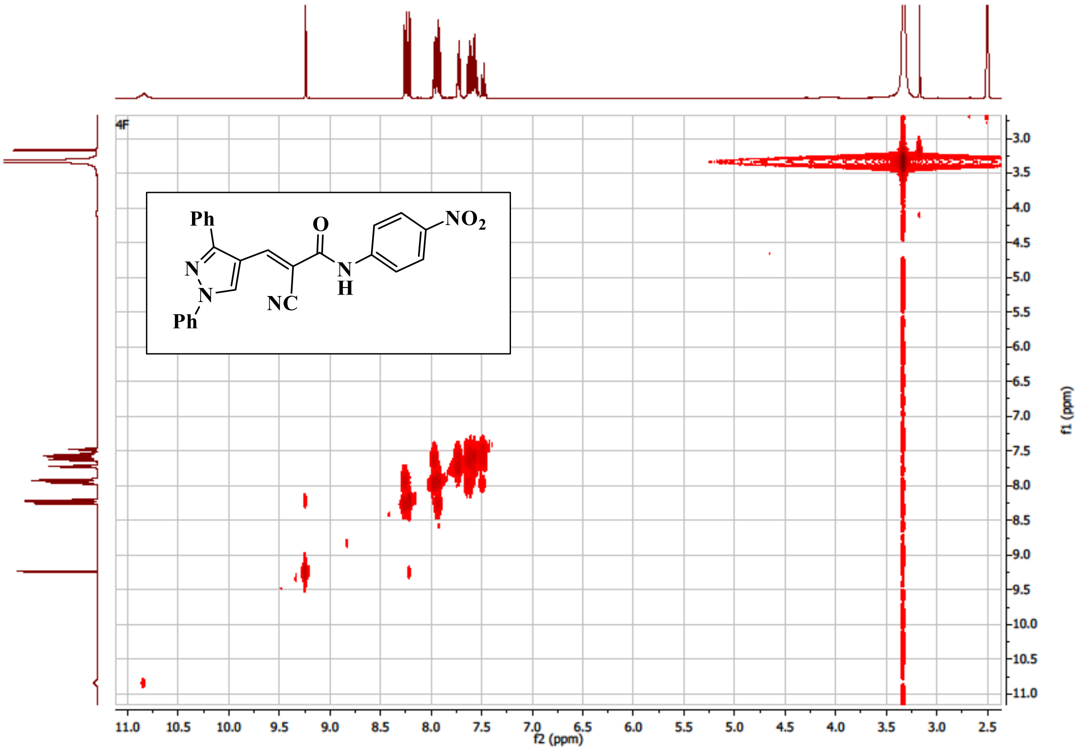

Figure S31: ^{1}H COSY spectrum of compound 3f

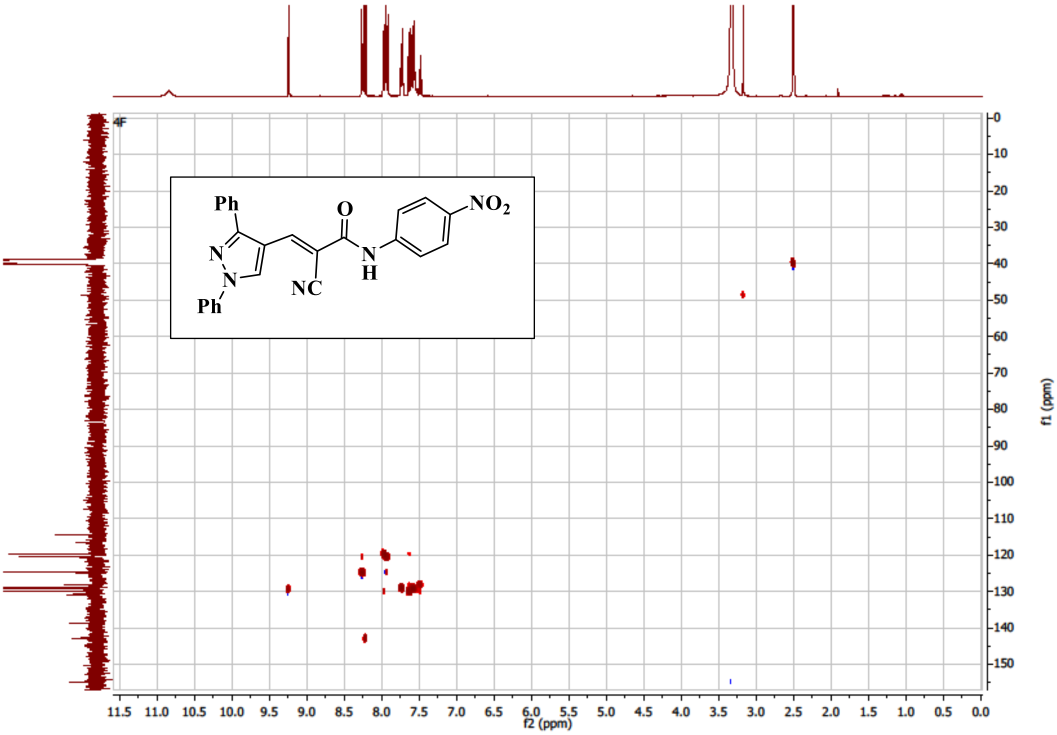

Figure S32: ¹H SQC spectrum of compound 3f

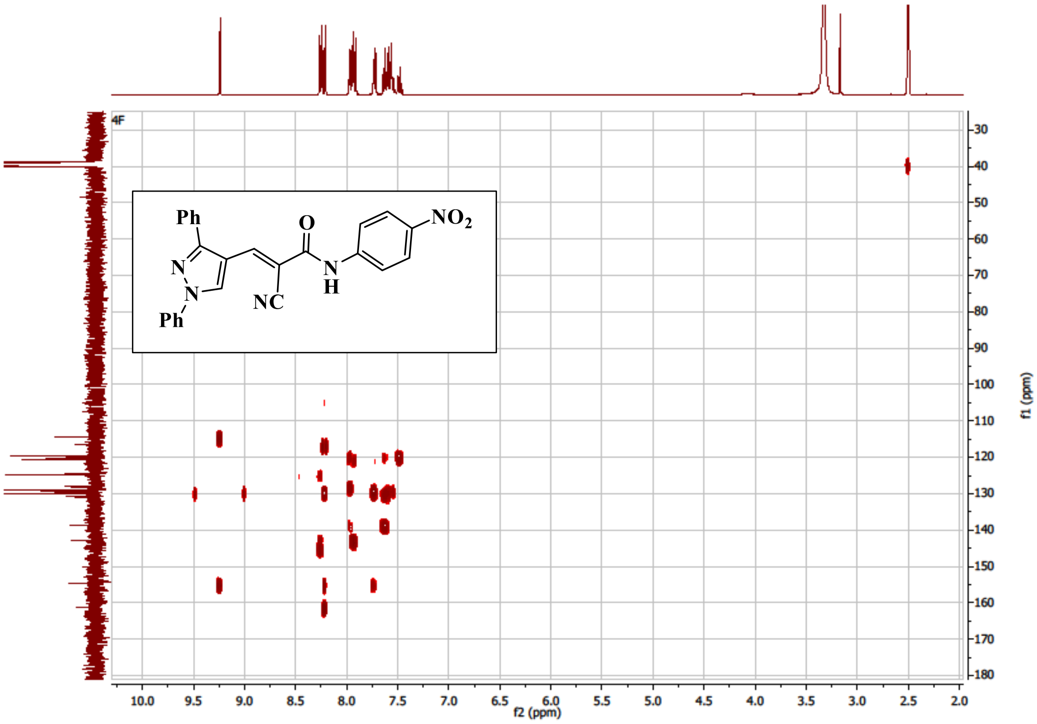

Figure S33: ¹H MBC spectrum of compound 3f

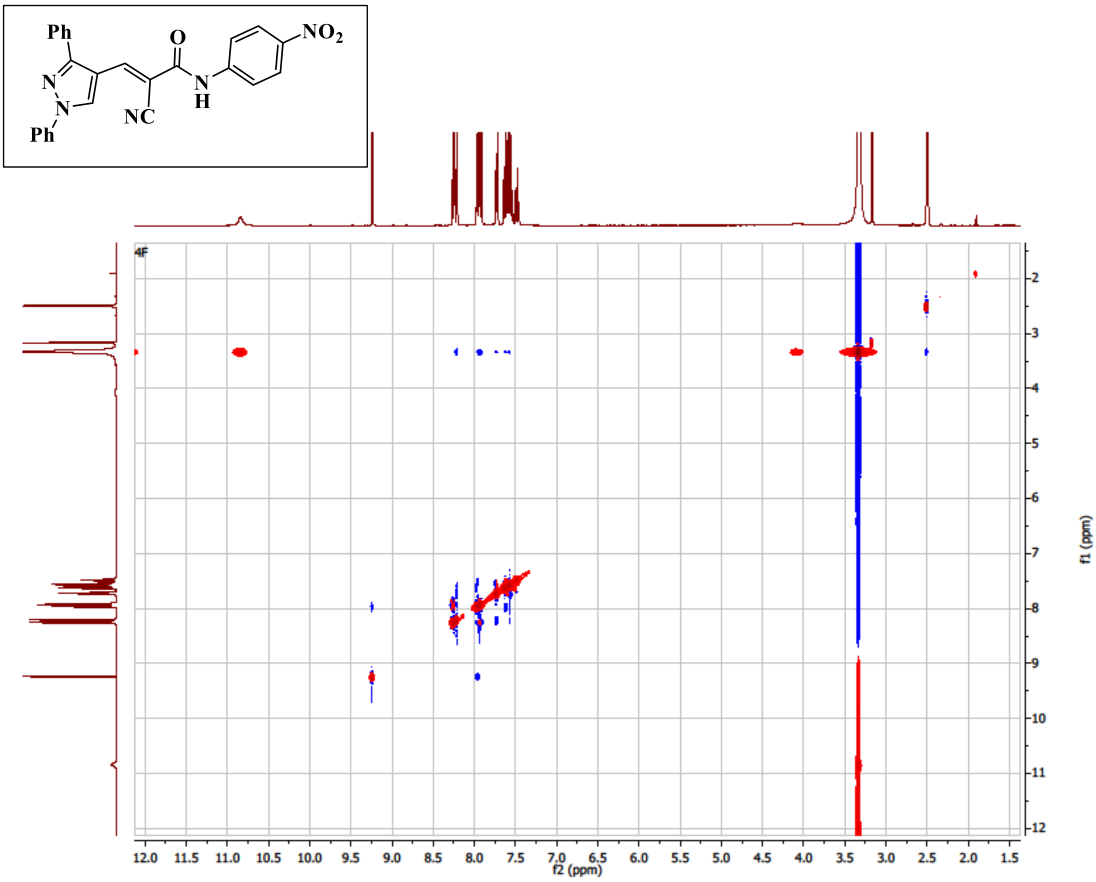

Figure S34: ^{1}H NOSEY spectrum of compound 3f

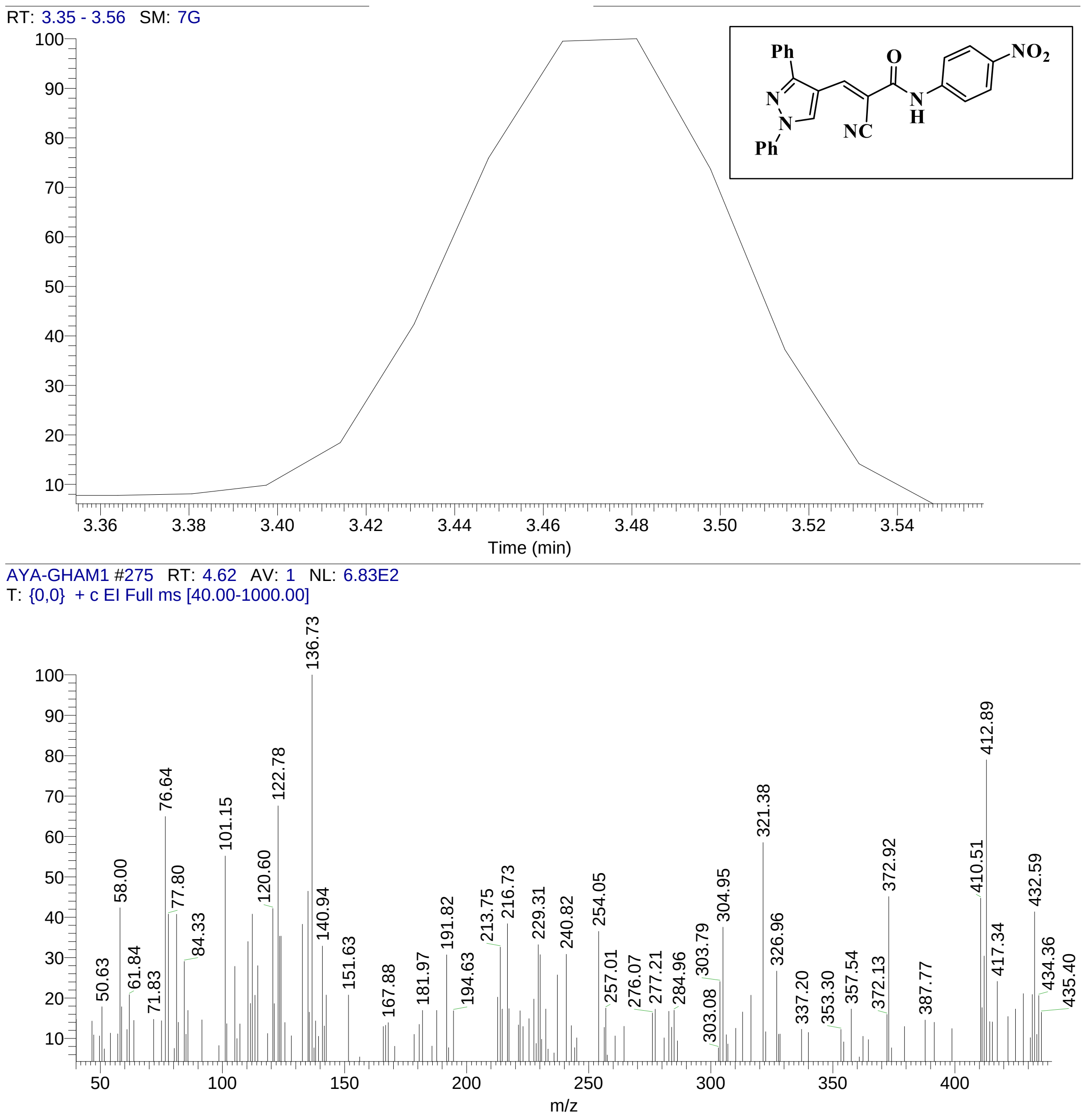

Figure S35: Mass spectrum of compound 3f

2-cyano-3-(1,3-diphenyl-1*H*-pyrazol-4-yl)-*N*-(thiazol-2-yl)acrylamide (4g)

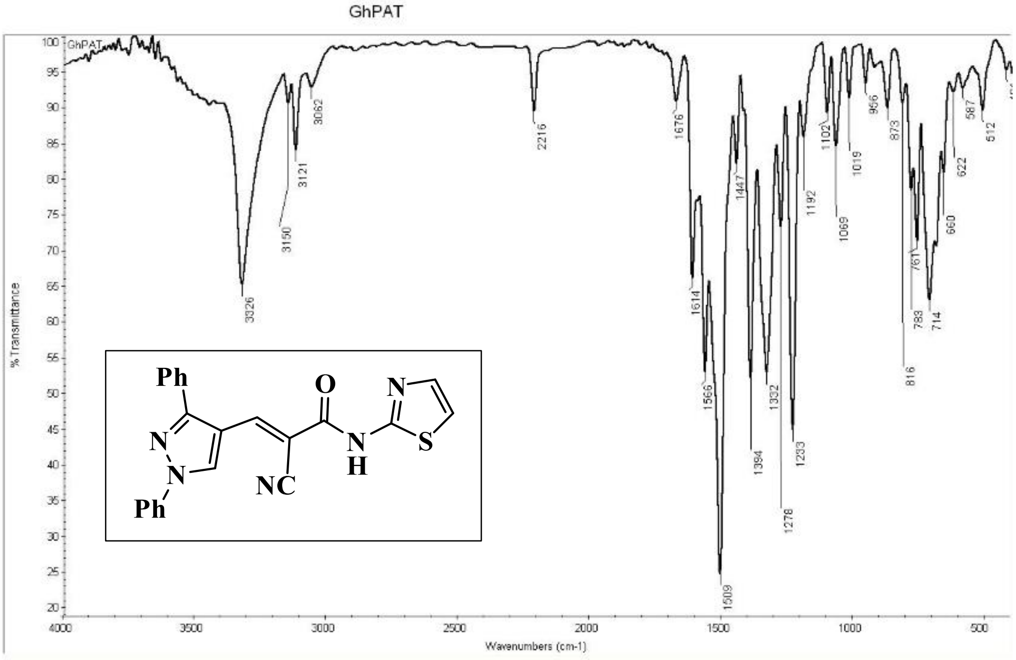

Figure S36: IR spectrum of compound 3g

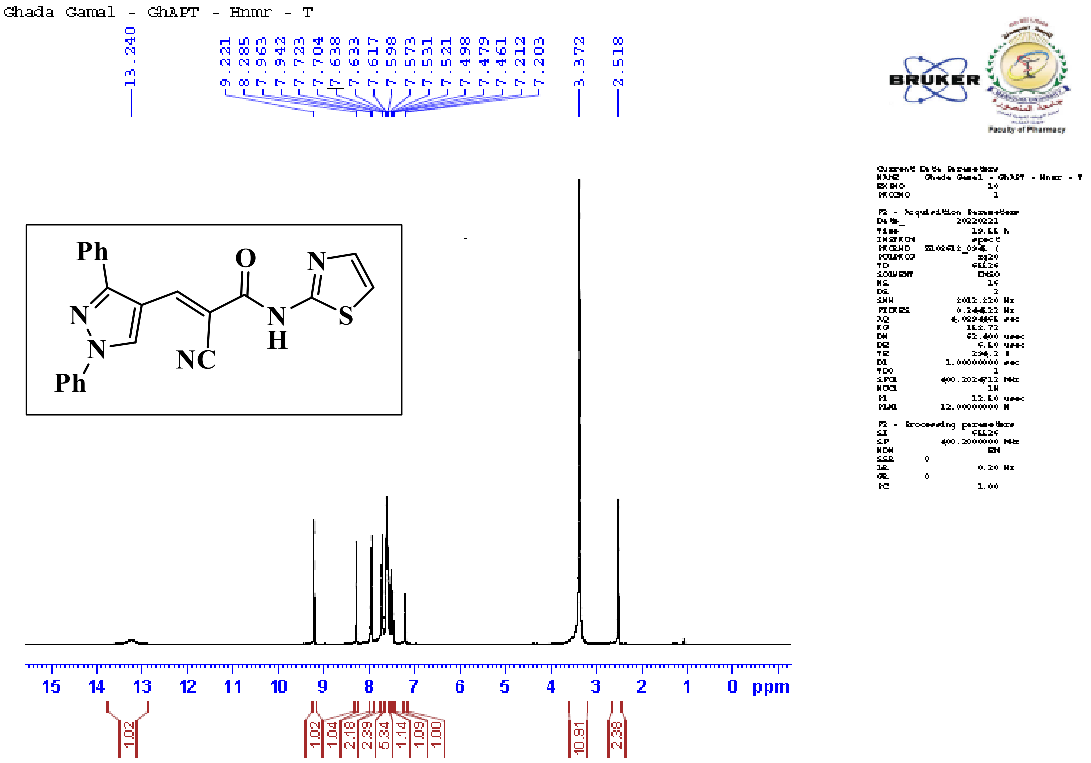

Figure S37: ^{1}H NMR spectrum of compound 3g

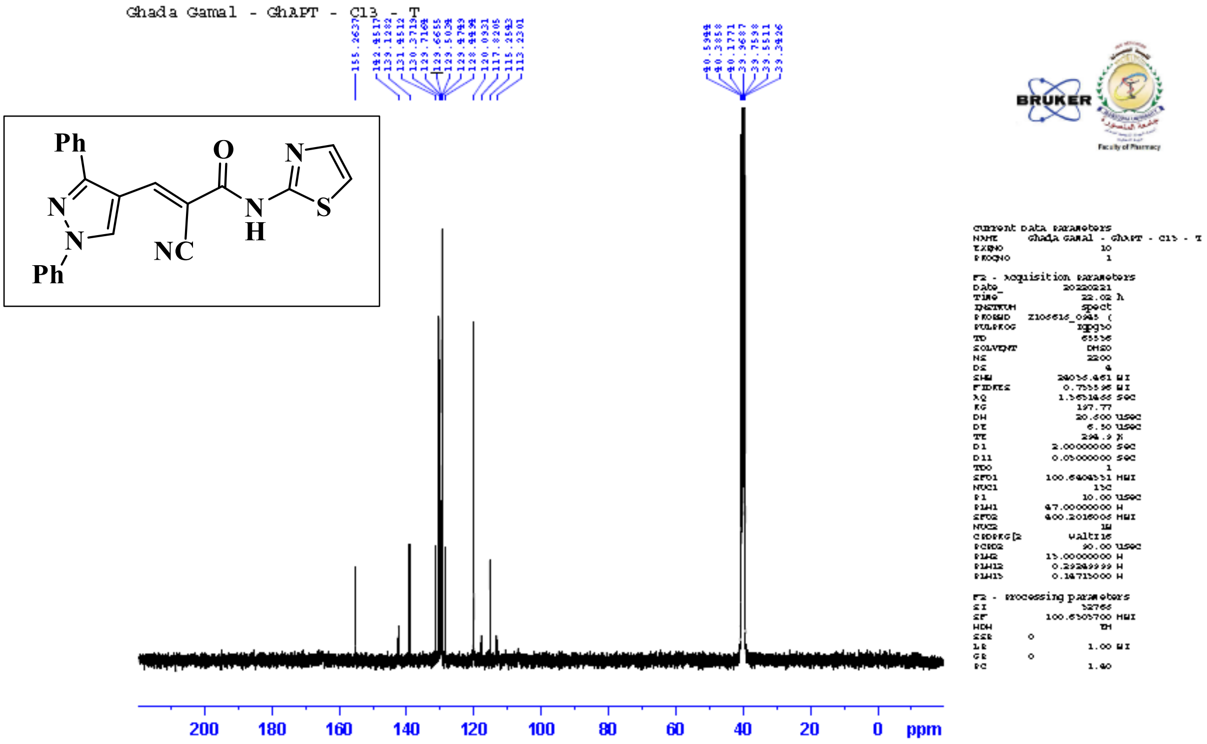

Figure S38: ^{13}C NMR spectrum of compound 3g

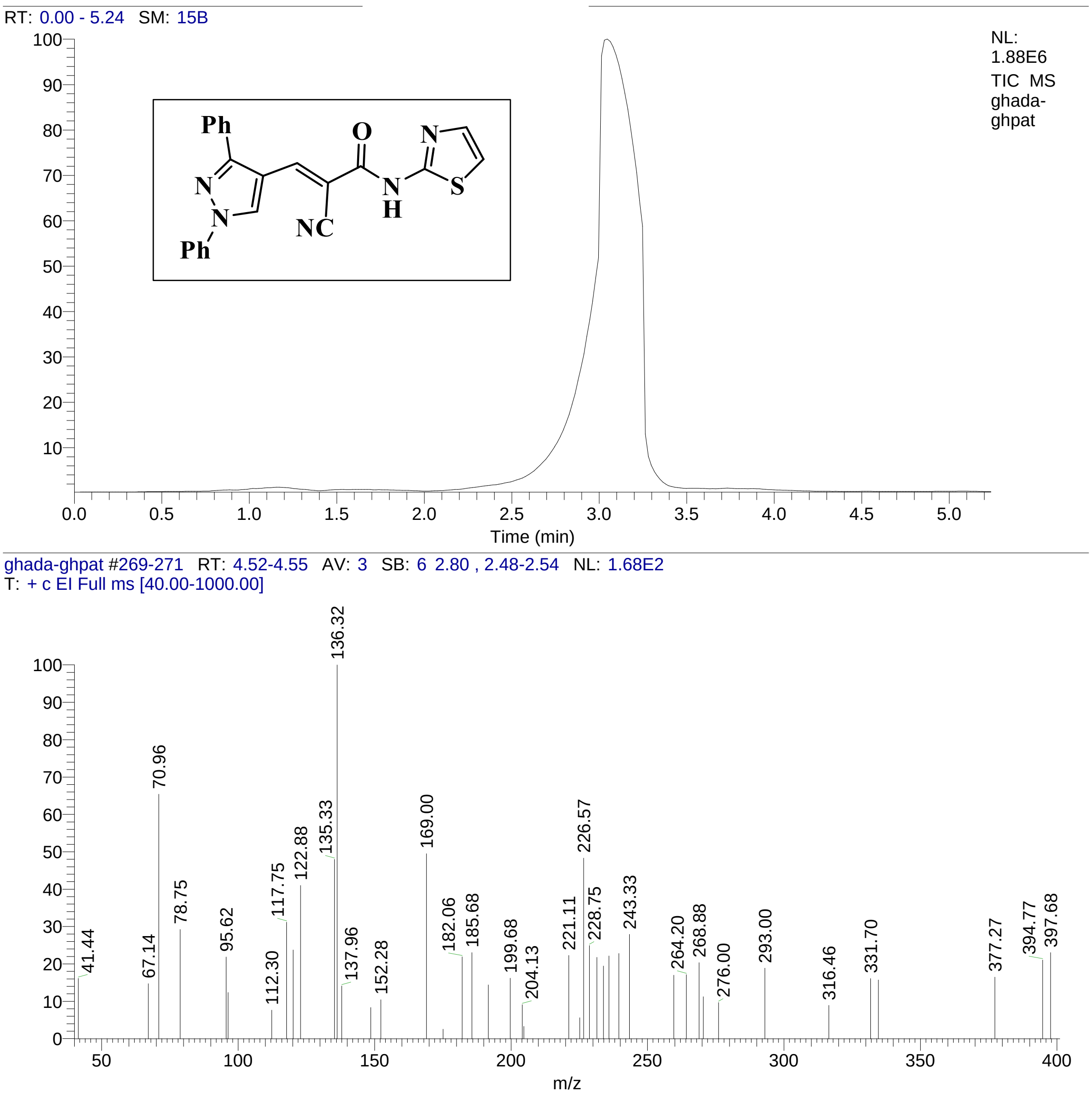

Figure S39: Mass spectrum of compound 3g

***N*-(benzo[*d*]thiazol-2-yl)-2-cyano-3-(1,3-diphenyl-1*H*-pyrazol-4-yl)acrylamide (3h):**

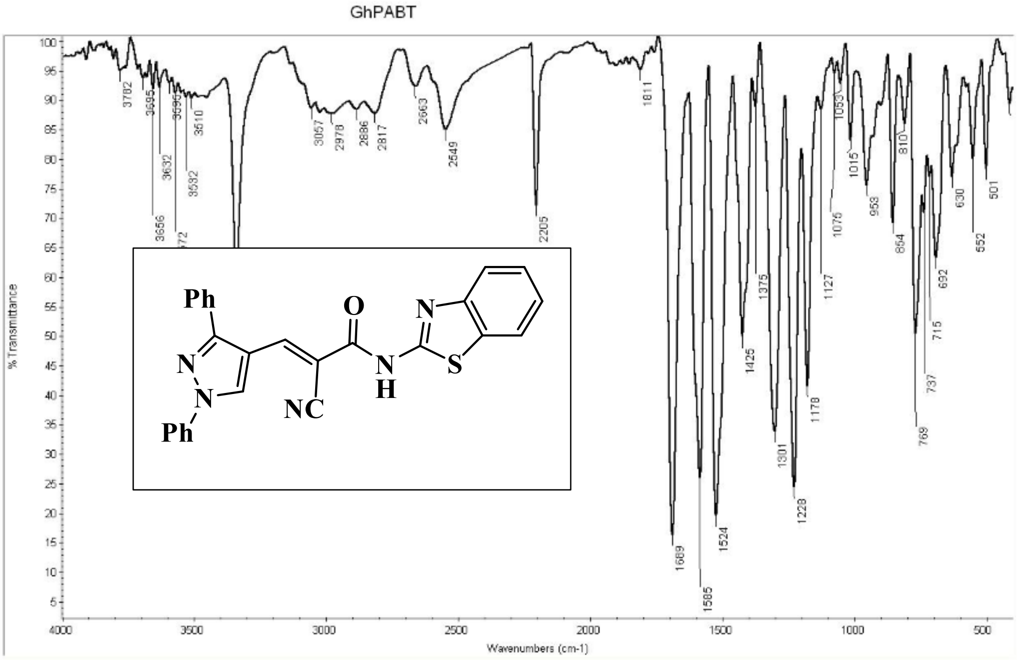

Figure S40: IR spectrum of compound 3h

4,5,6,7-tetrabromo-2-(((1,3-diphenyl-1H-pyrazol-4-yl)methylene)amino)isoindoline-1,3-dione (6)

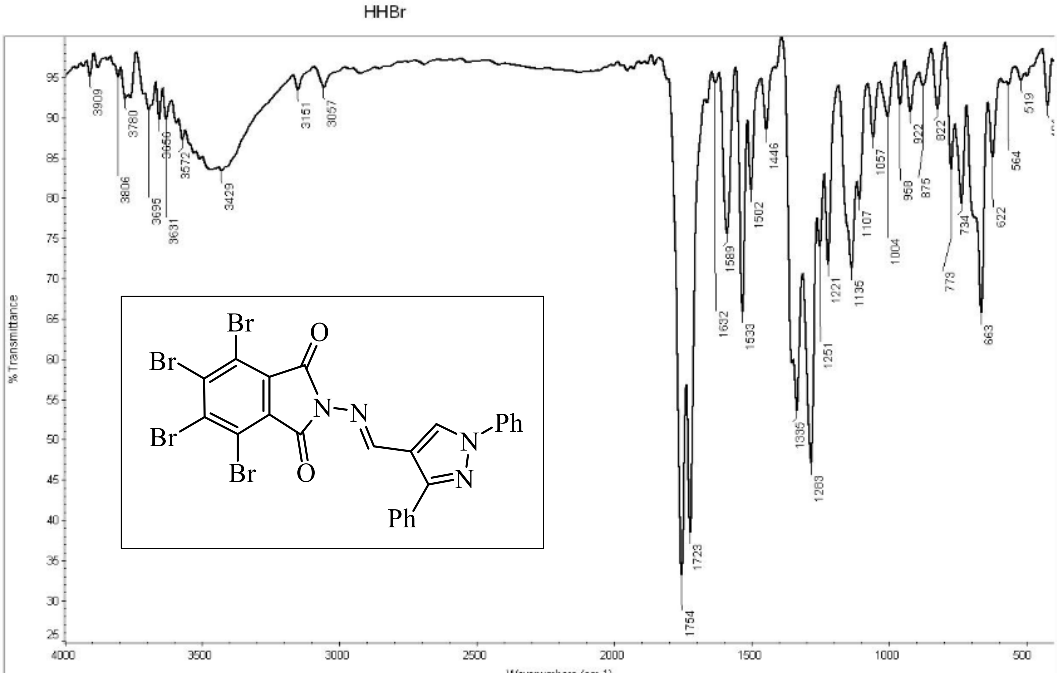

Figure S41: IR spectrum of compound 6

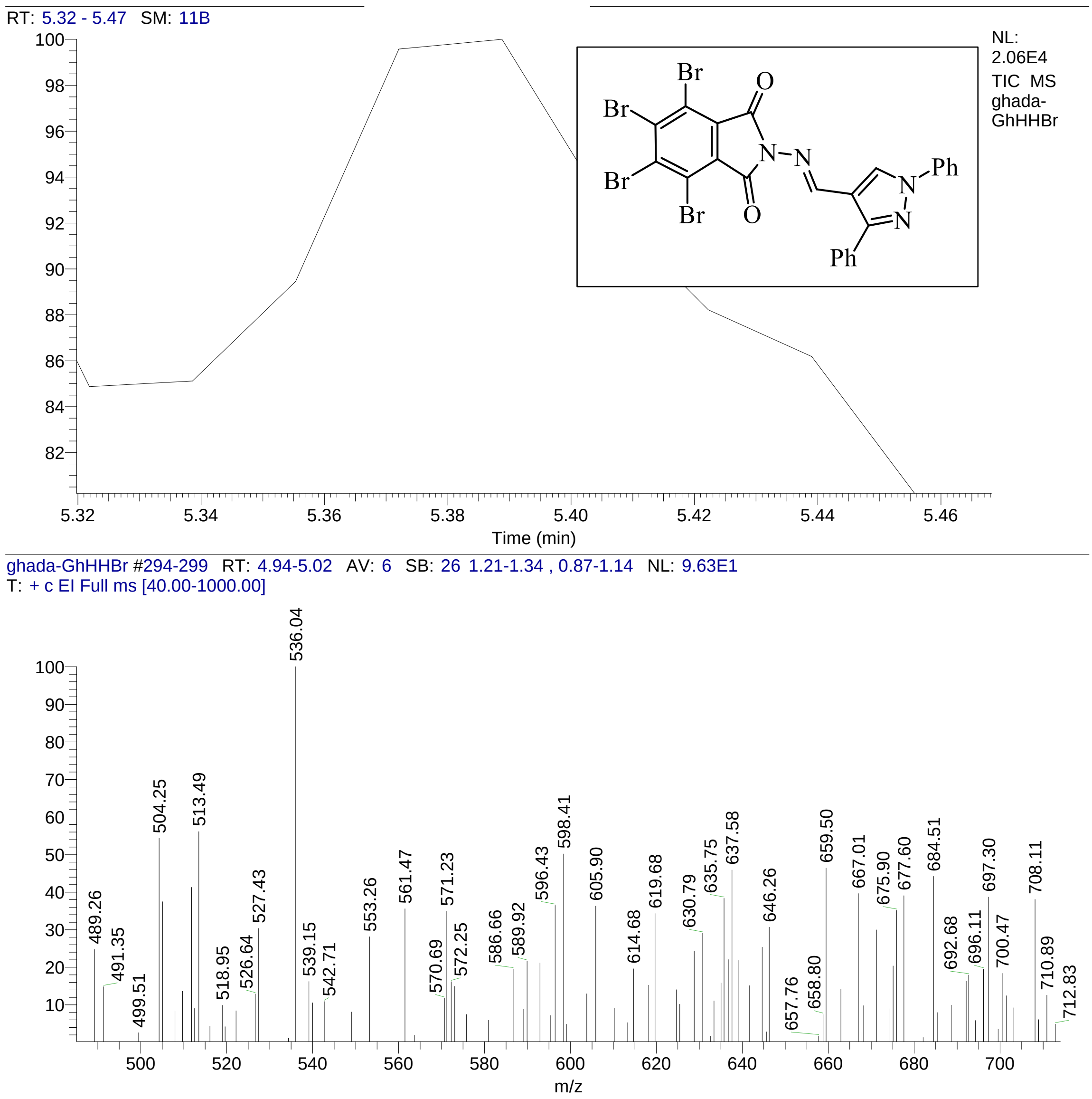

Figure S42: Mass spectrum of compound 6

5-(1,3-diphenyl-1*H*-pyrazol-4-yl)-3-oxo-2,3-dihydro-1*H*-pyrazole-4-carbonitrile (7):

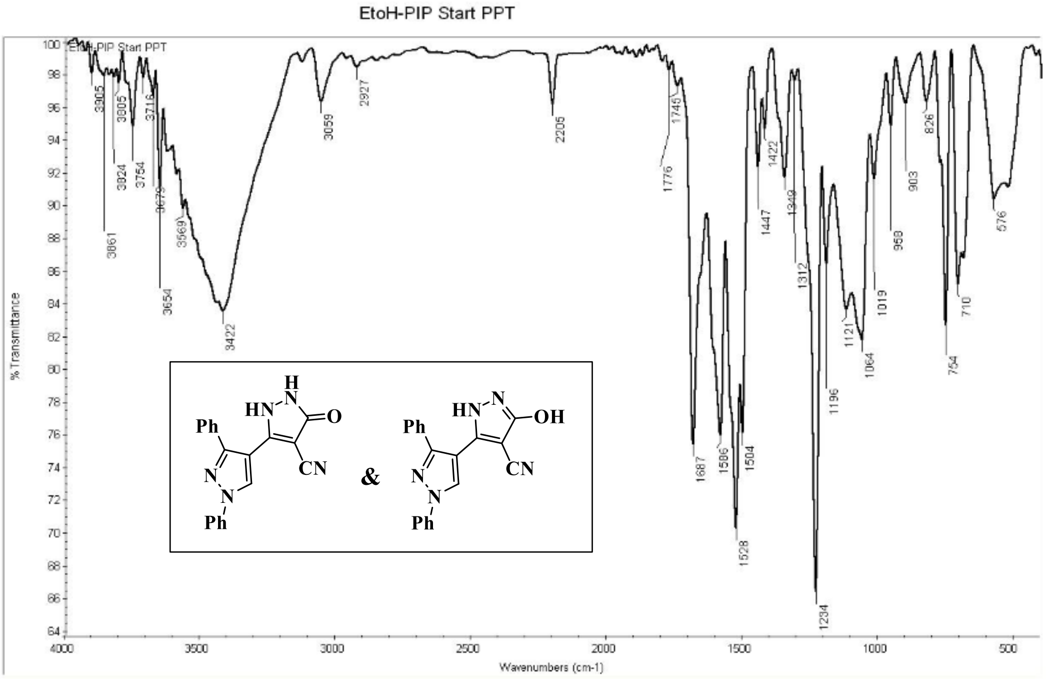

Figure S43: IR spectrum of compound 7

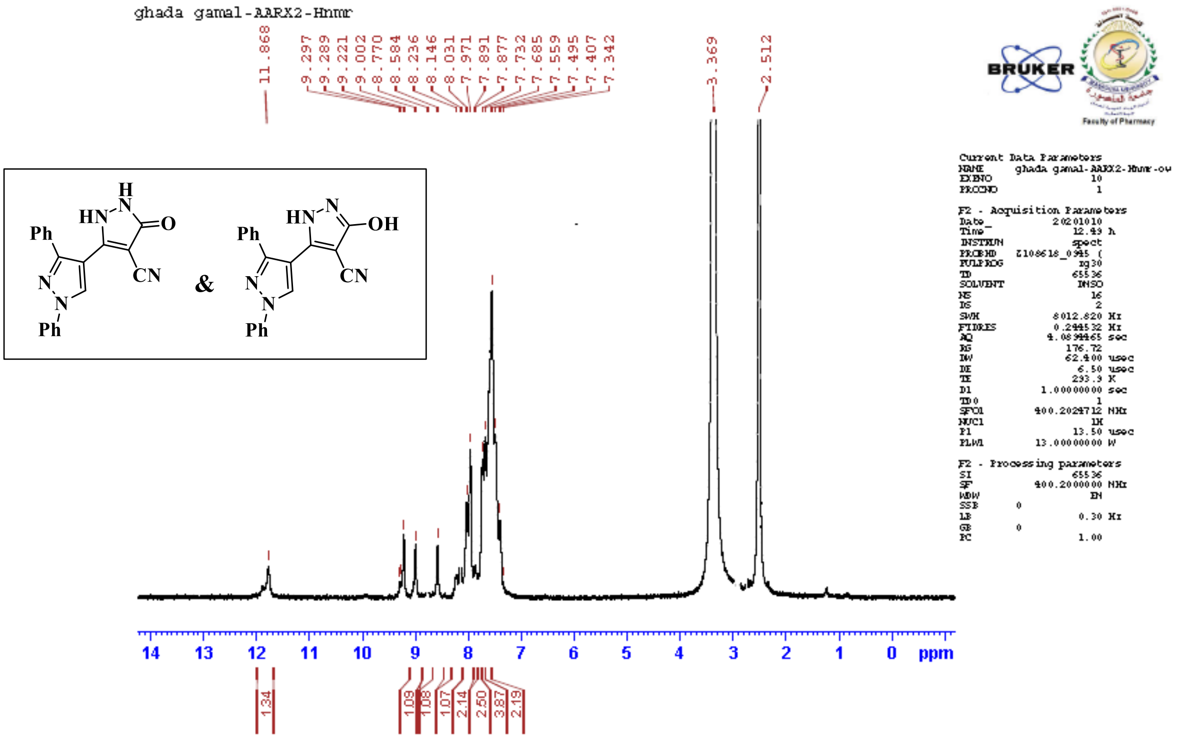

Figure S44: ^{1}H NMR spectrum of compound 7

2-cyano-*N*'-((1,3-diphenyl-1*H*-pyrazol-4-yl)methylene)acetohydrazide (8)

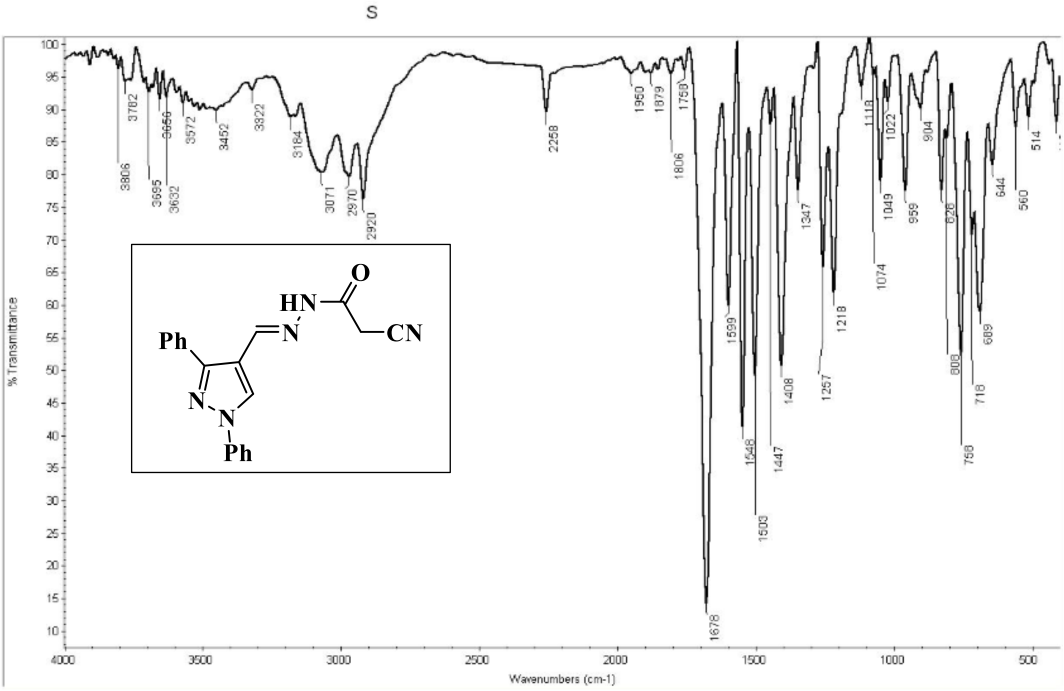

Figure S45: IR spectrum of compound 8

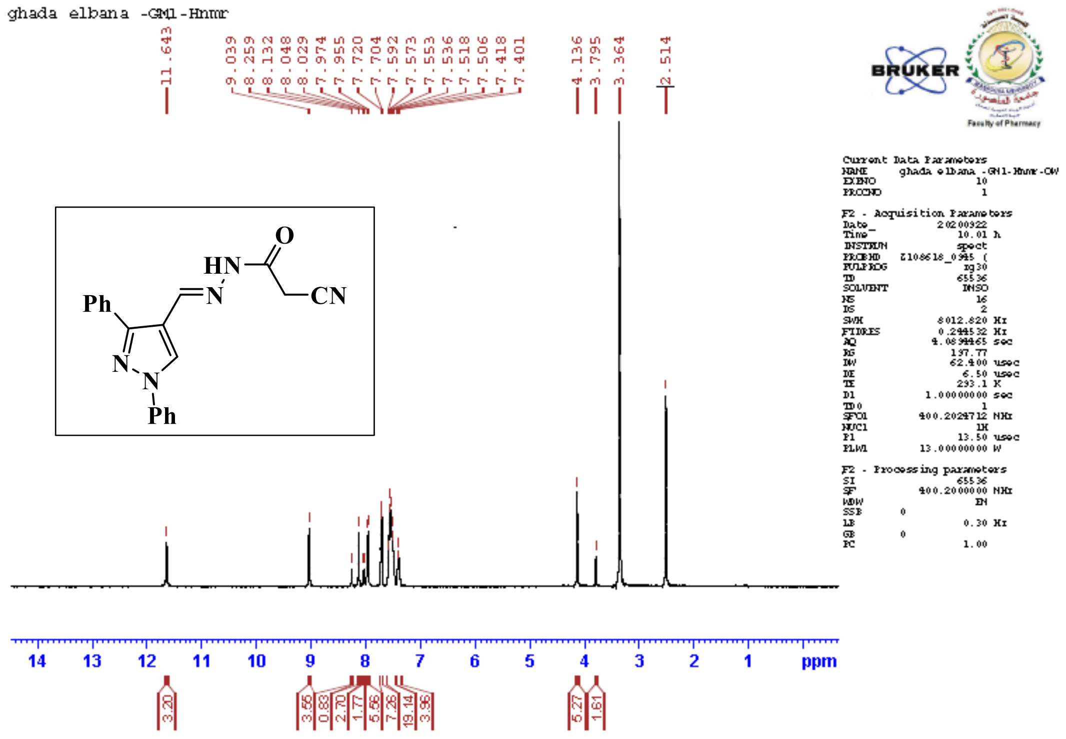

Figure S46: ^{1}H NMR spectrum of compound 8

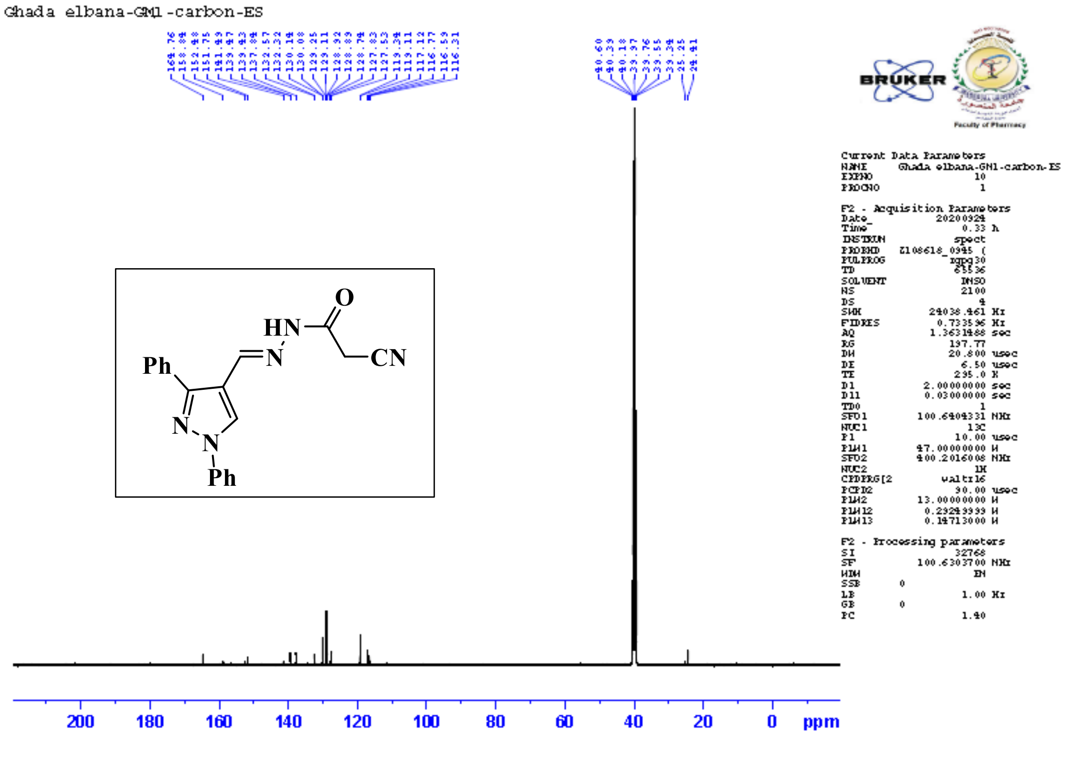

Figure S47: ^{13}C NMR spectrum of compound 8

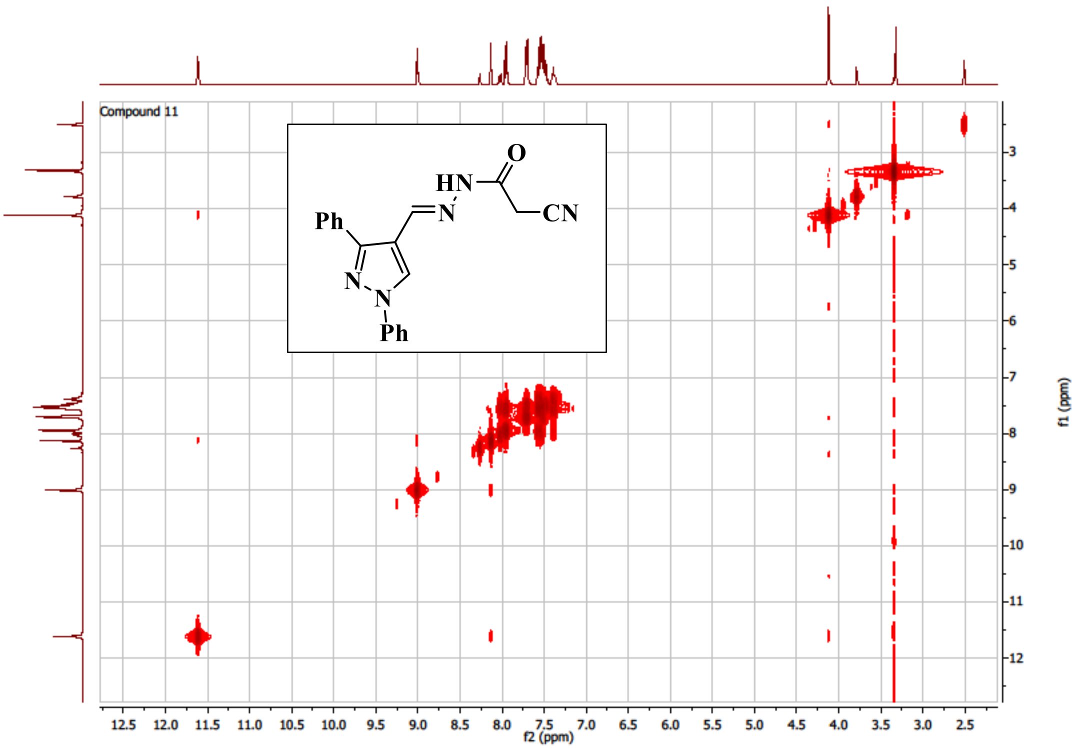

Figure S48: ^{1}H COSY spectrum of compound 8

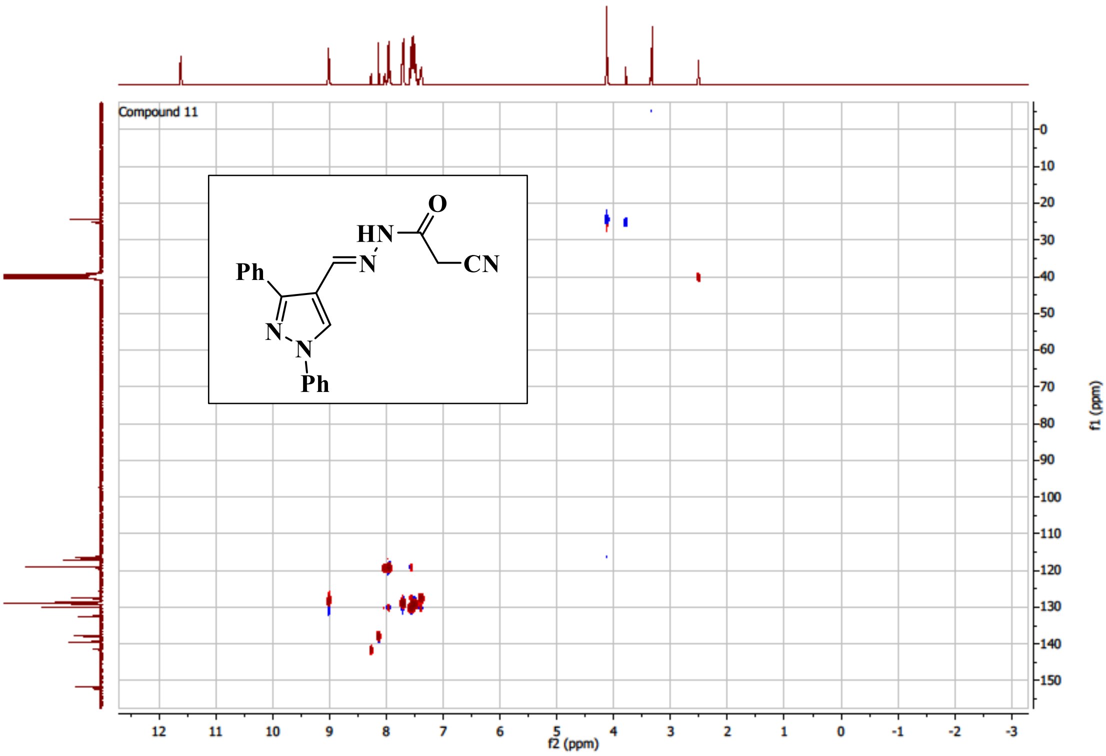

Figure S49: ^{1}H SQC spectrum of compound 8

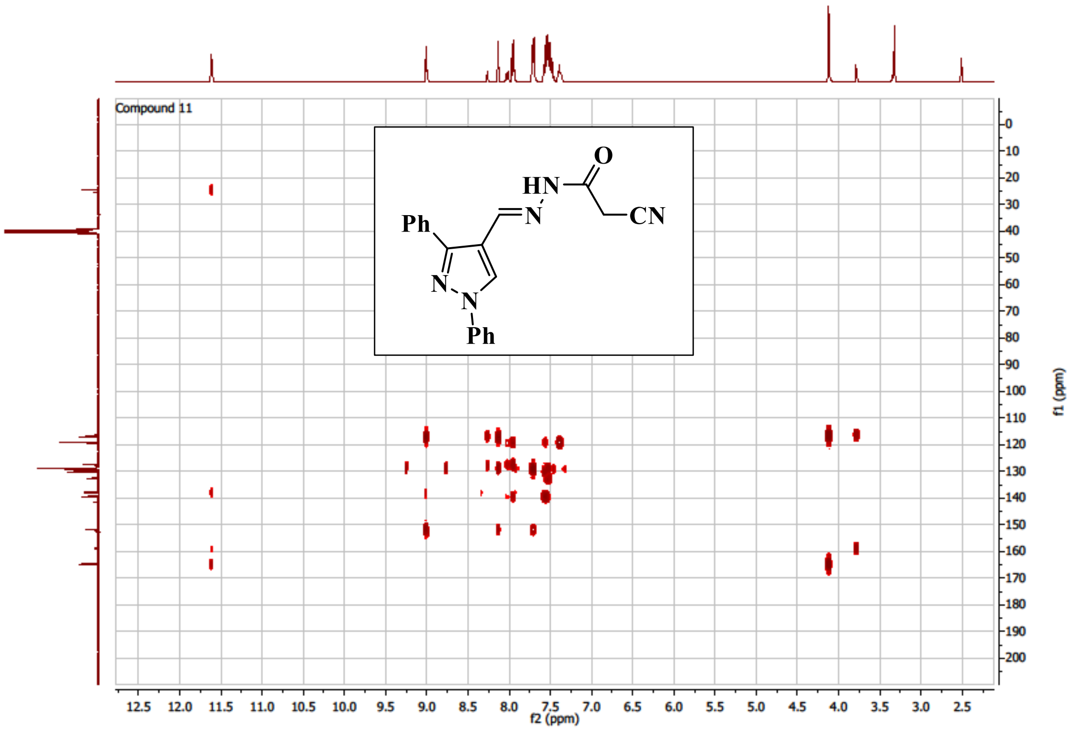

Figure S50: ^{1}H MBC spectrum of compound 8

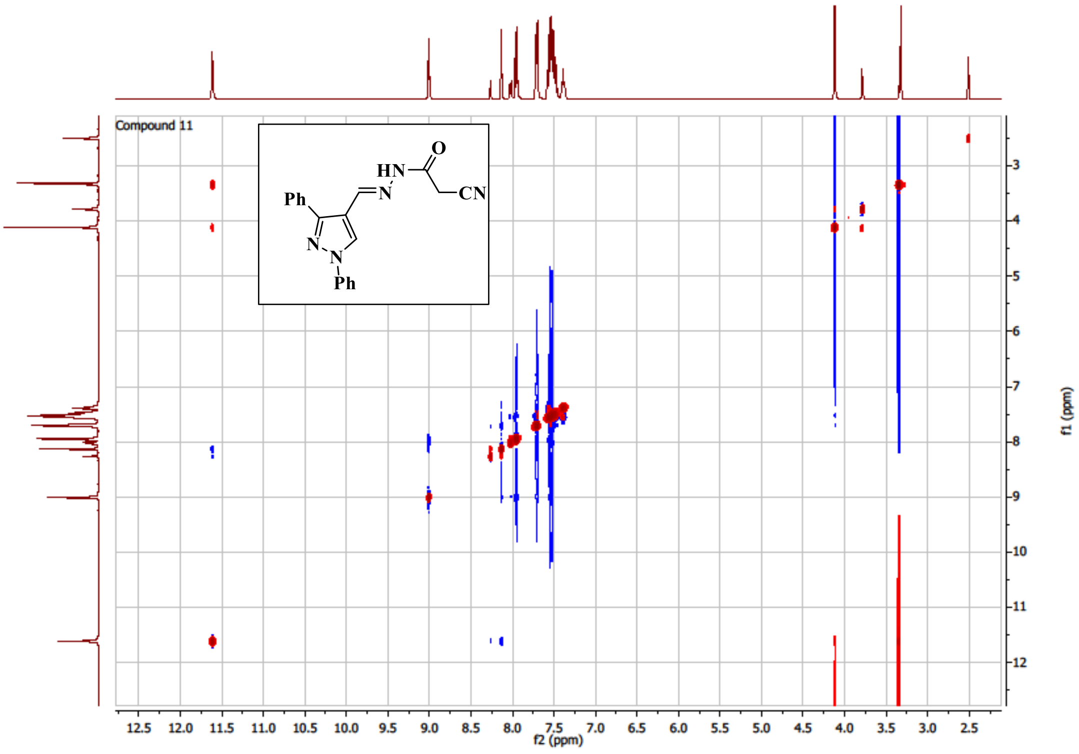

Figure S51: ^{1}H NOSY spectrum of compound 8

***N'*-((1,3-diphenyl-1*H*-pyrazol-4-yl)methylene)-2-oxo-2*H*-chromene-3-carbohydrazide (10a)**

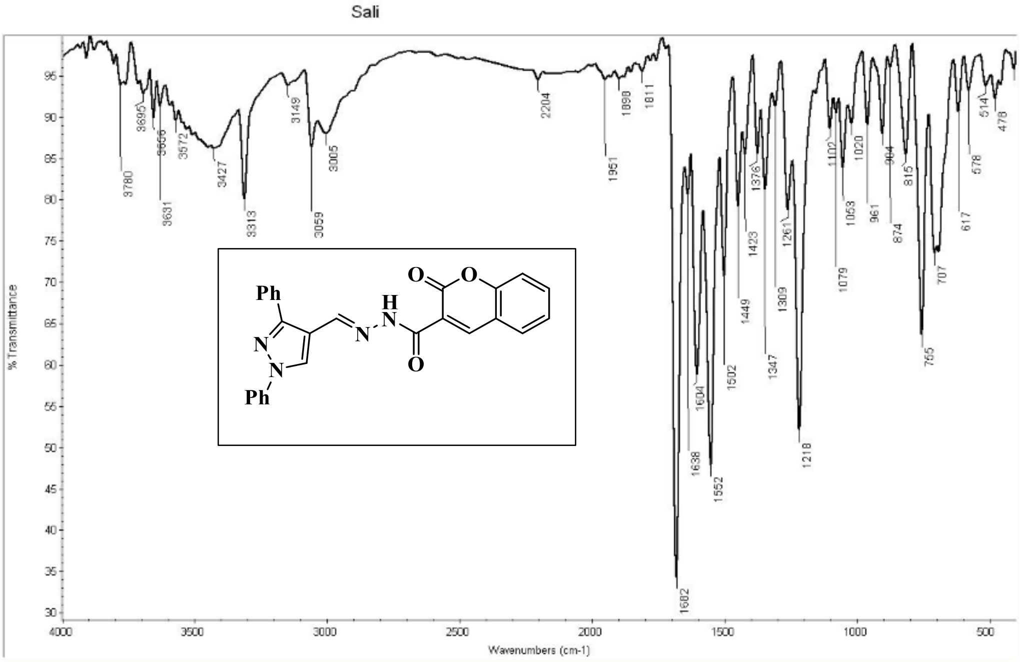

Figure S52: IR spectrum of compound 10a

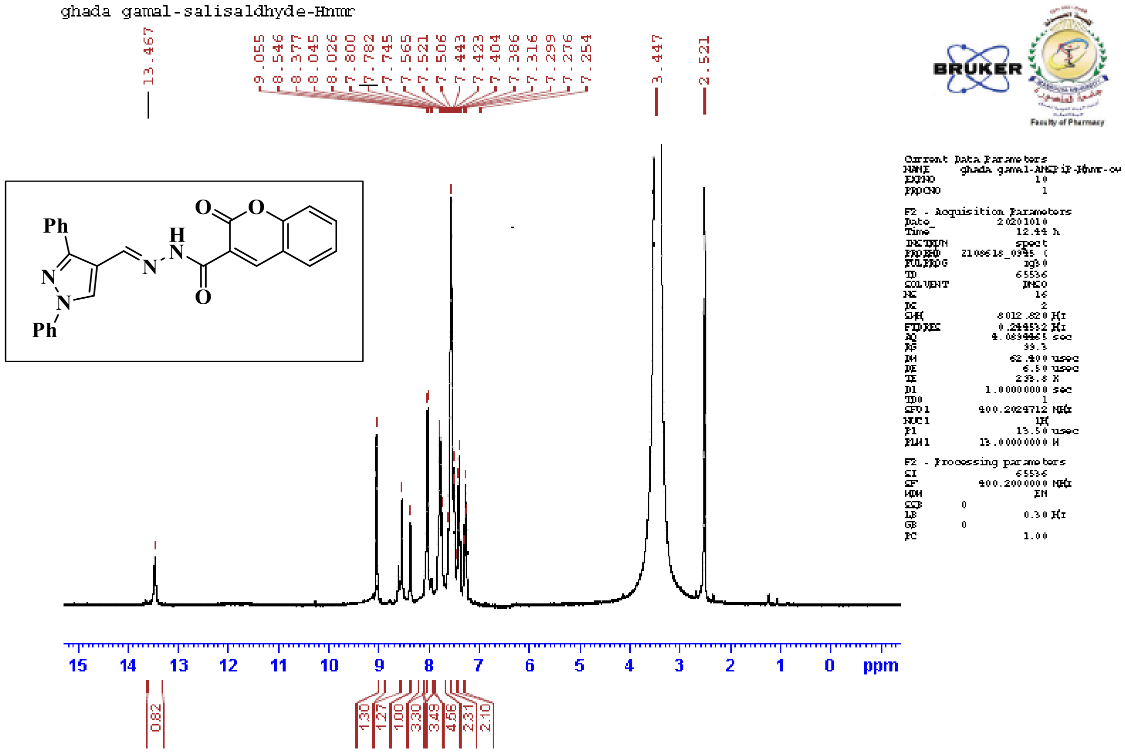

Figure S53: ^{1}H NMR spectrum of compound 10a

***N*'-((1,3-diphenyl-1*H*-pyrazol-4-yl)methylene)-2-oxo-6-(*p*-tolydiazenyl)-2*H*-chromene-3-carbo-hydrazide (10b)**

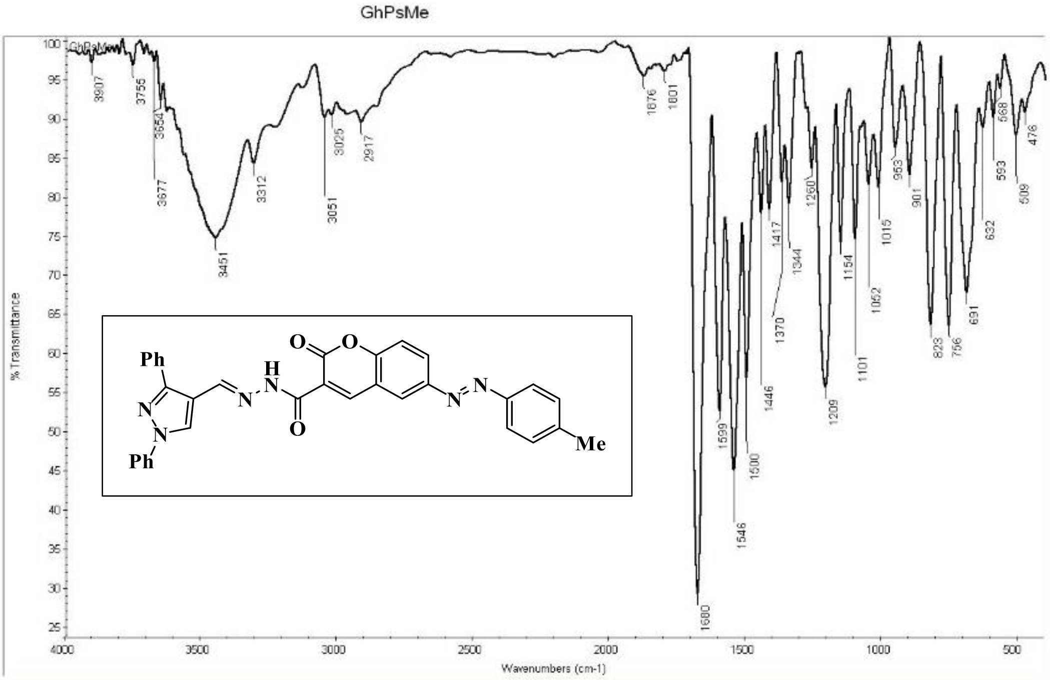

Figure S54: IR spectrum of compound 10b

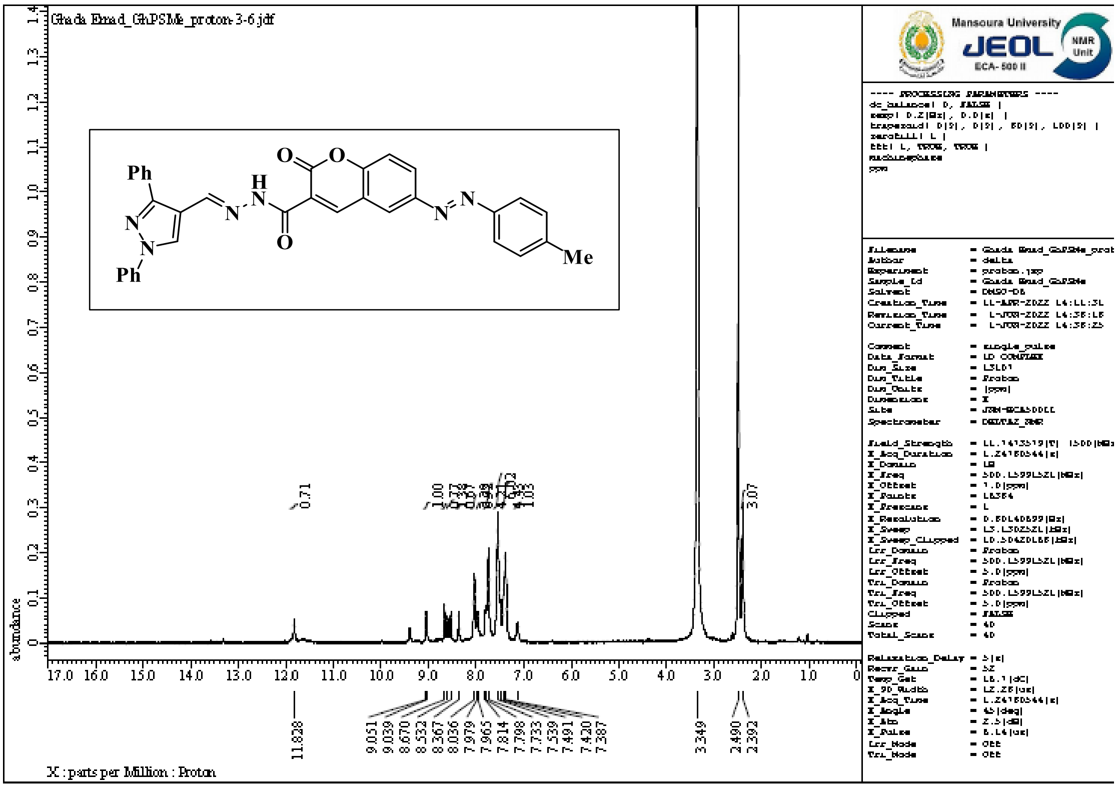

Figure S55: ^{1}HNMR spectrum of compound 10b

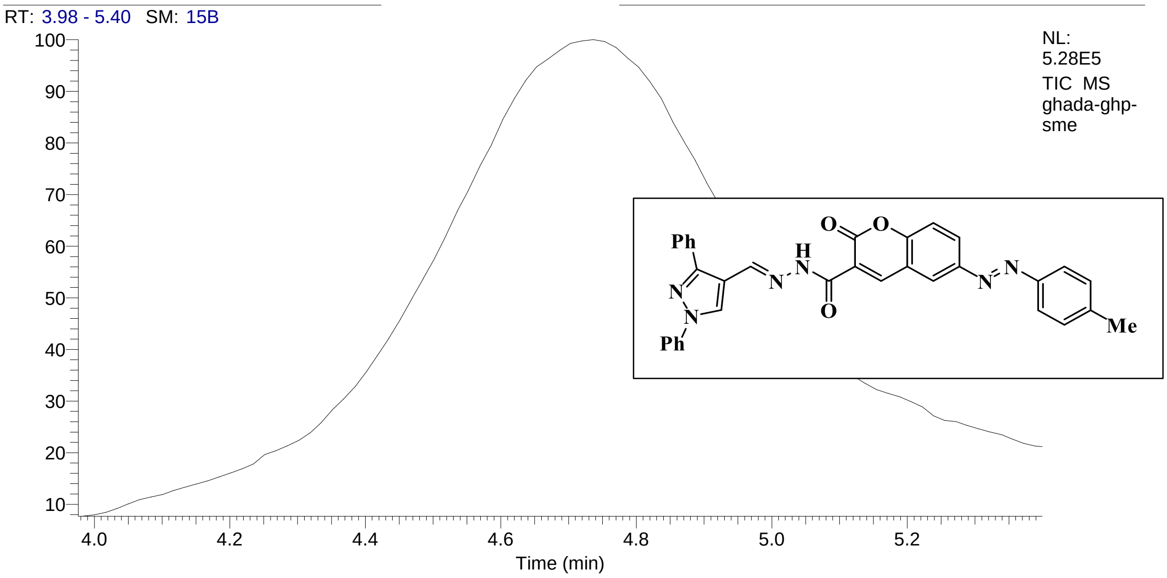

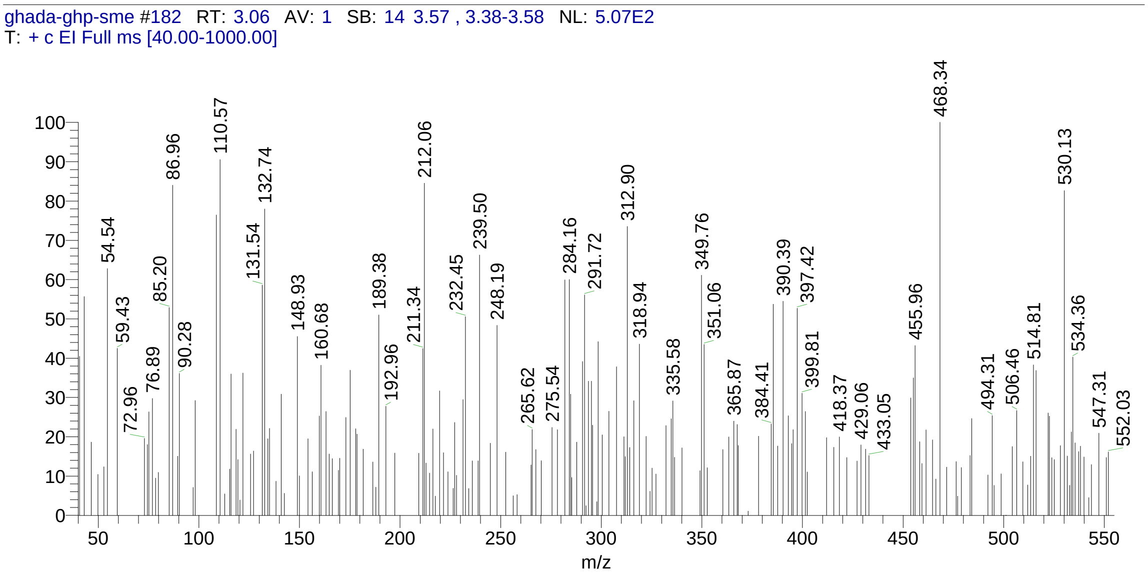

Figure S56: Mass spectrum of compound 10b

6-((4-chlorophenyl)diazenyl)-*N*'-(1,3-diphenyl-1*H*-pyrazol-4-yl)methylene)-2-oxo-2*H*-chromene-3-carbohydrazide (10c)

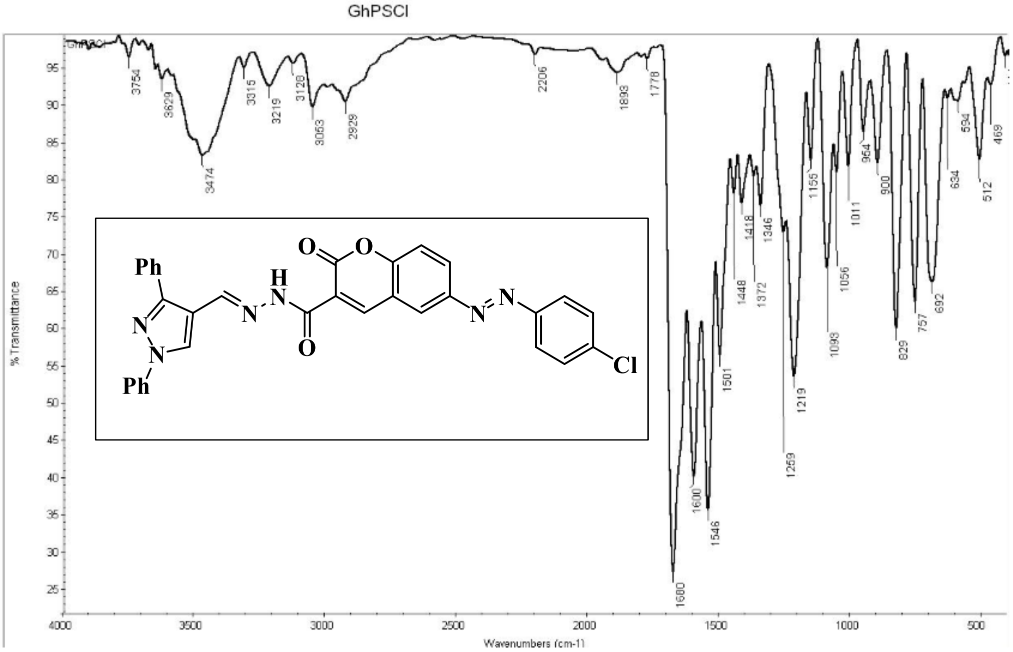

Figure S57: IR spectrum of compound 10c

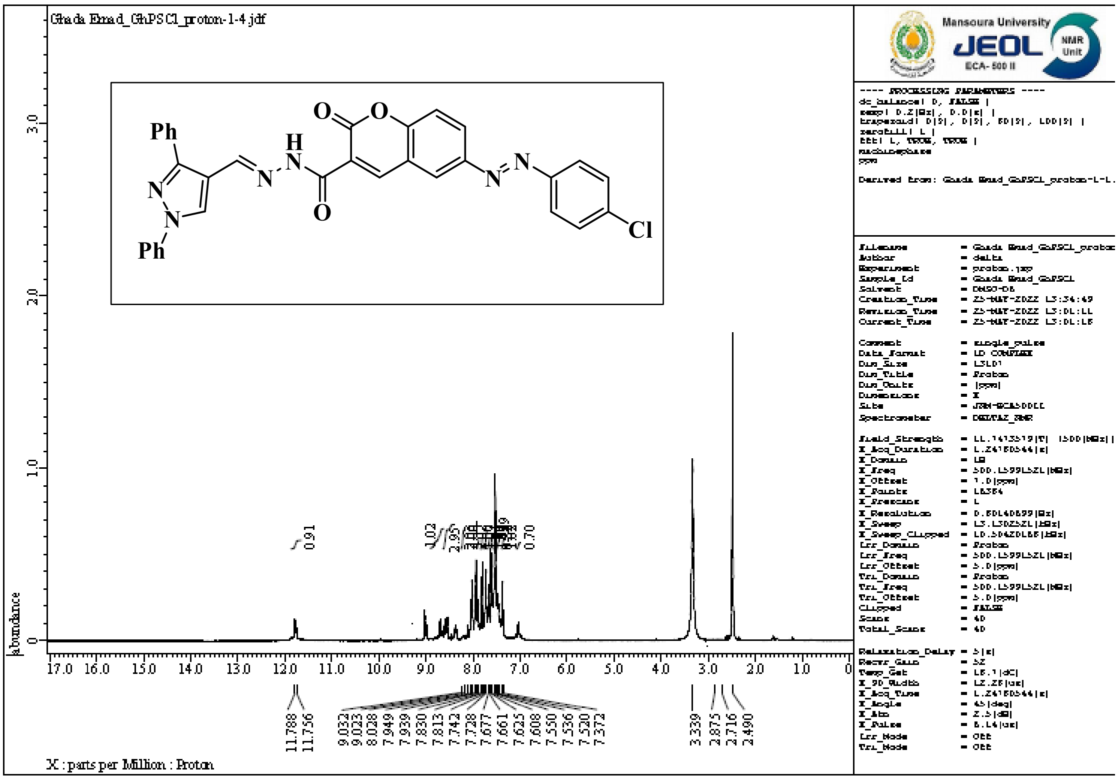

Figure S57: ^{1}H NMR spectrum of compound 10c

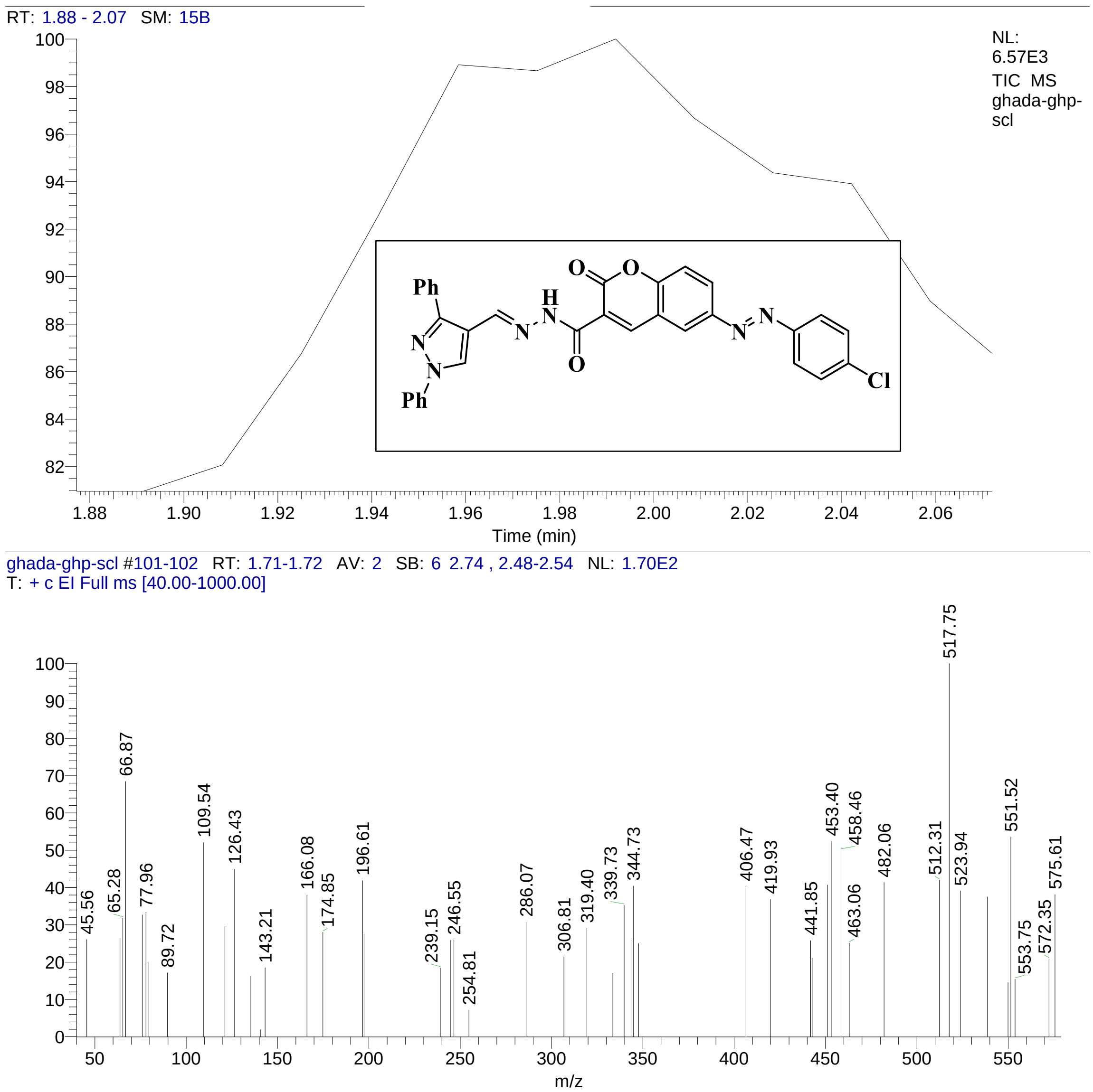

Figure S58: Mass spectrum of compound 10c

***N*'-((1,3-diphenyl-1*H*-pyrazol-4-yl)methylene)-6-((4-nitrophenyl)diazenyl)-2-oxo-2*H*-chromene-3-carbohydrazide (10d)**

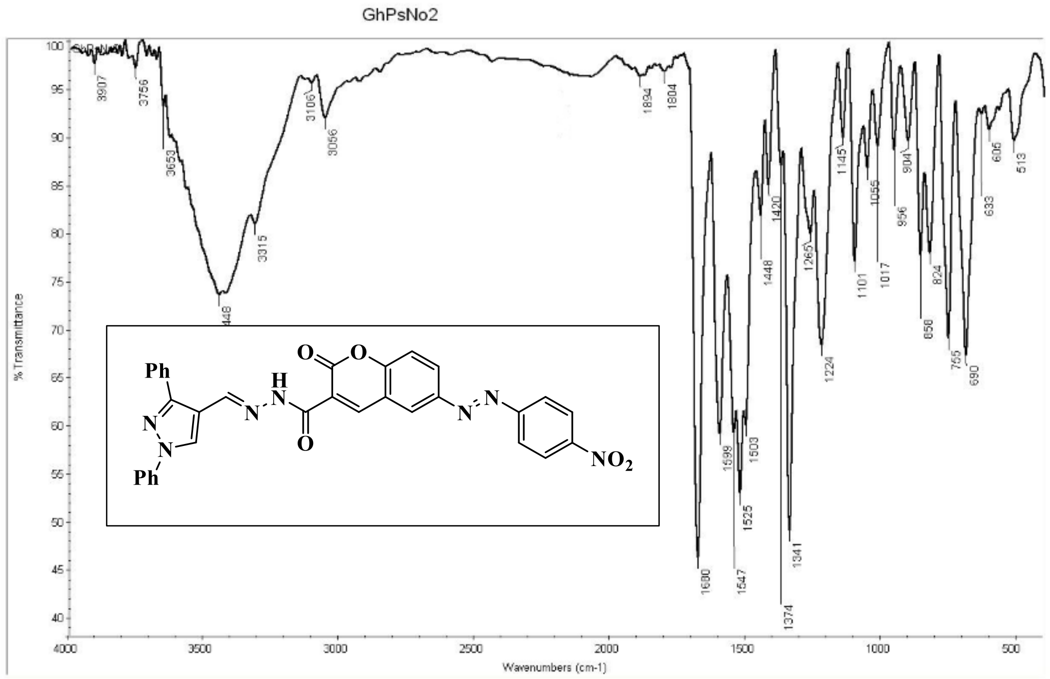

Figure S59: IR spectrum of compound 10d

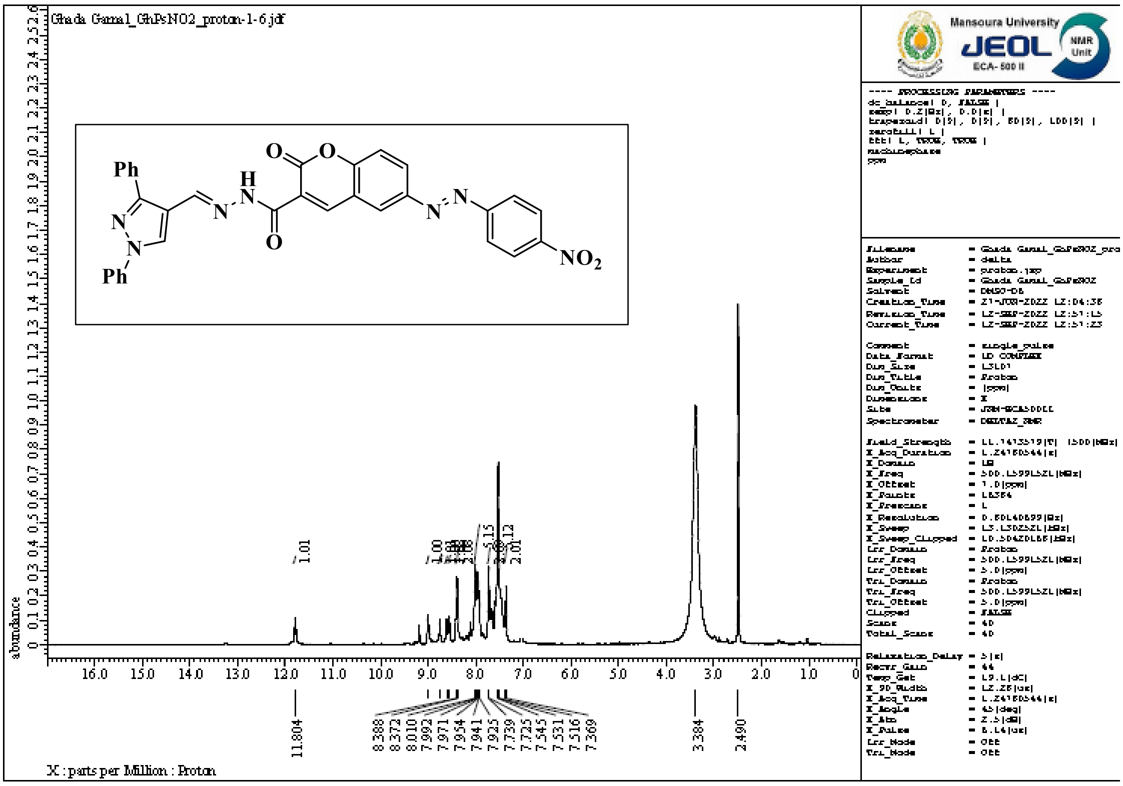

Figure S60: ^{1}H NMR spectrum of compound 10d

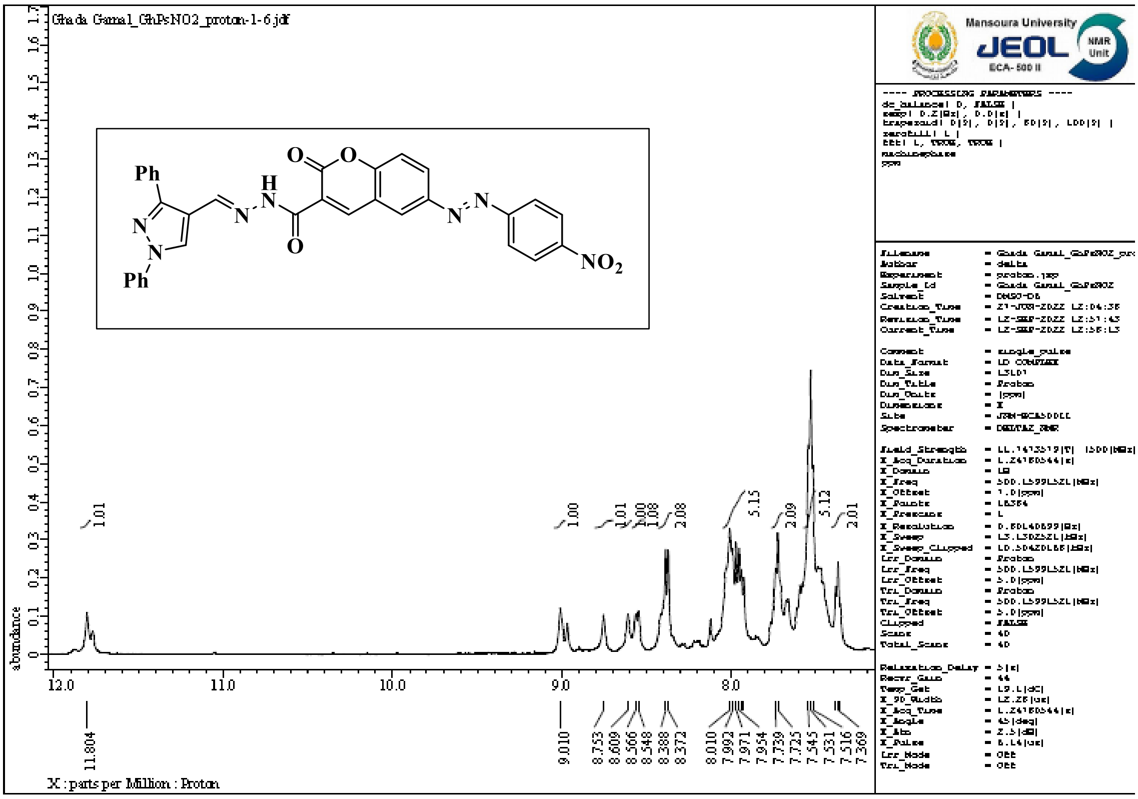

Figure S61: ^{1}H NMR spectrum of compound 10d

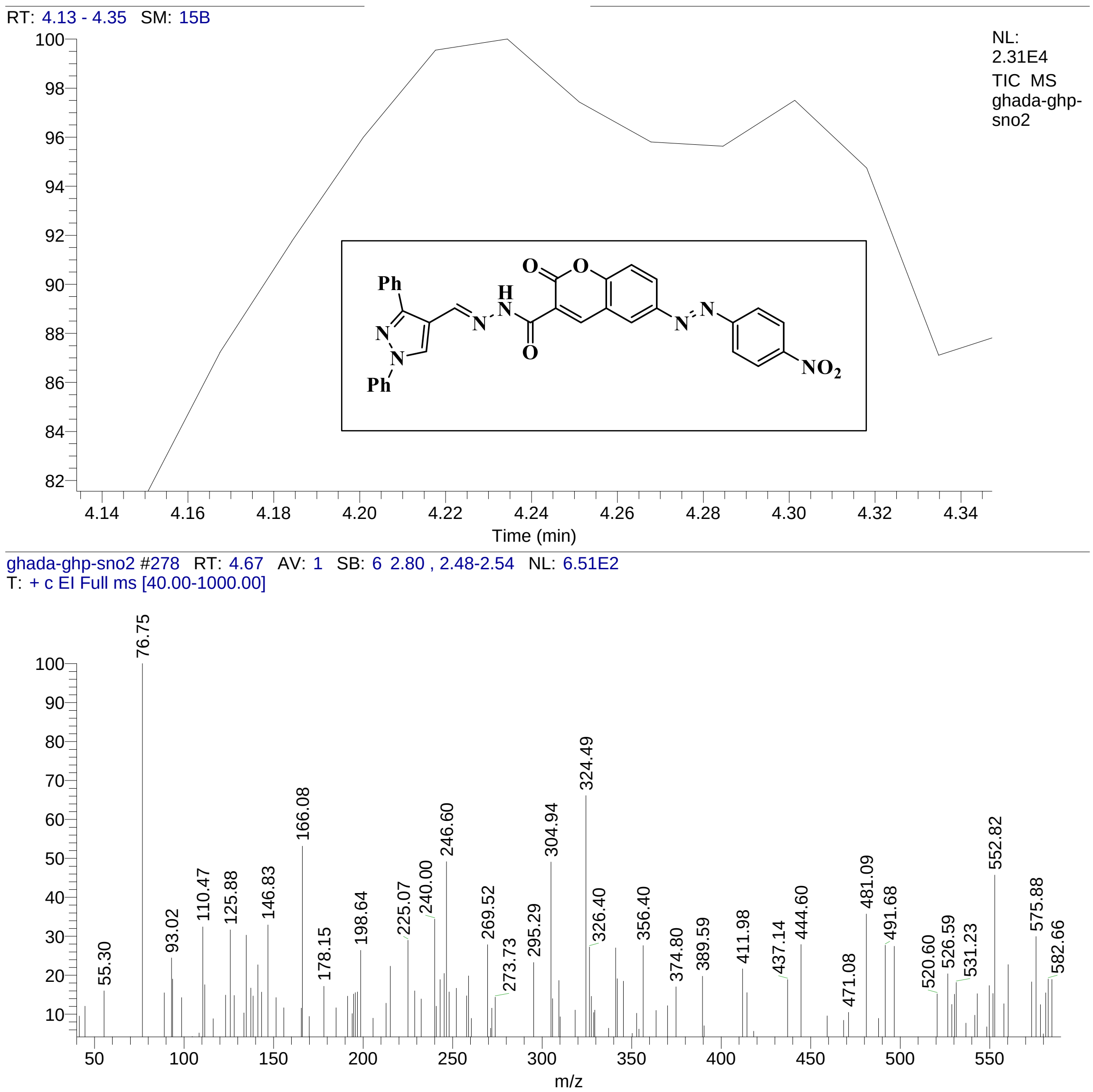

Figure S62: Mass spectrum of compound 10d

4-((3-(2-(1,3-diphenyl-1*H*-pyrazol-4-yl)methylene)hydrazine-1-carbonyl)-2-oxo-2*H*-chromen-6-yl)diazenyl)benzoic acid (10e)

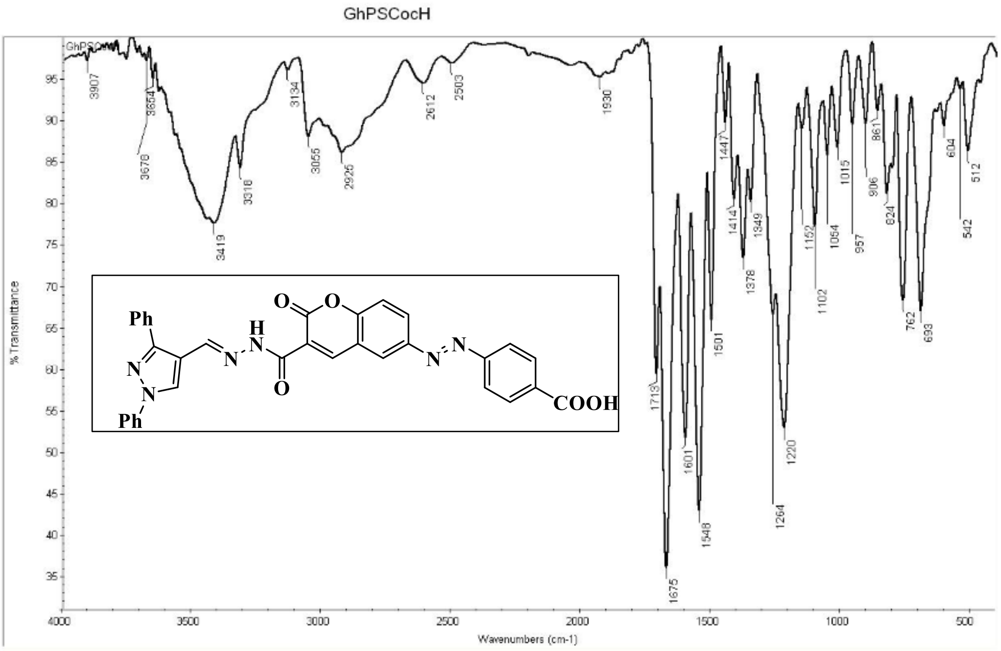

Figure S63: IR spectrum of compound 10e

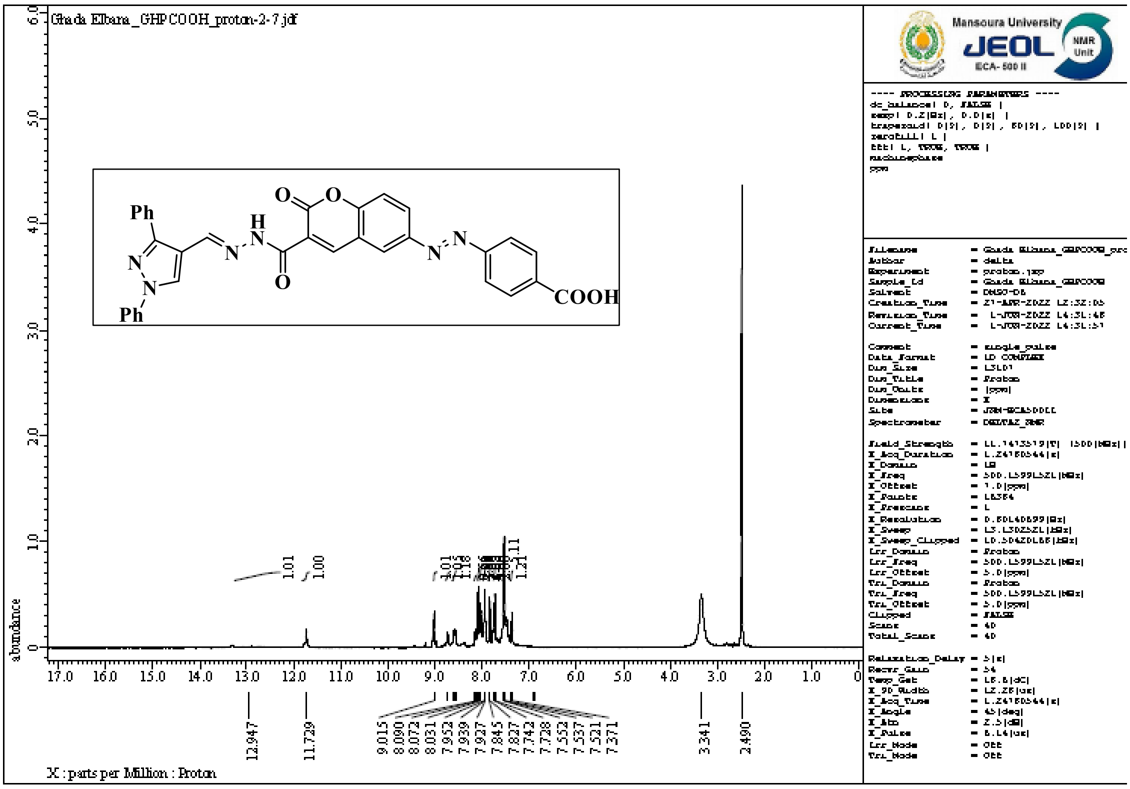

Figure S64: ^{1}H NMR spectrum of compound 10e

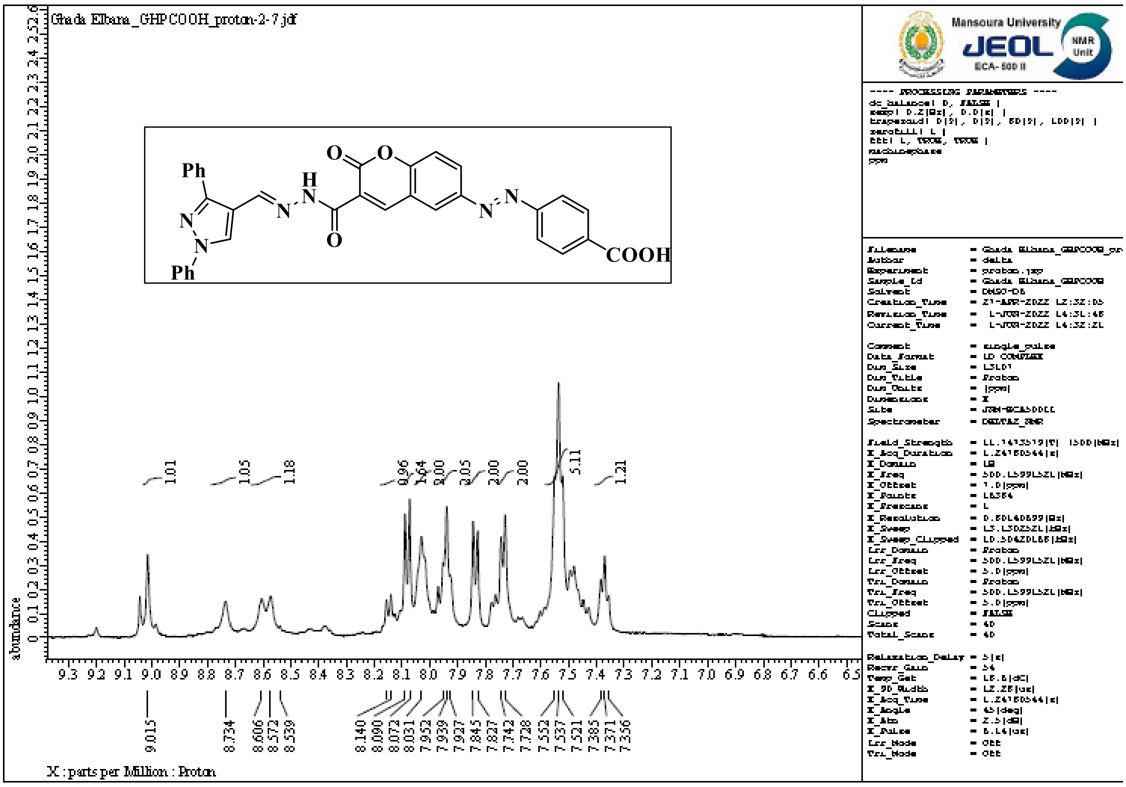

Figure S65: ^{1}H NMR spectrum of compound 10e

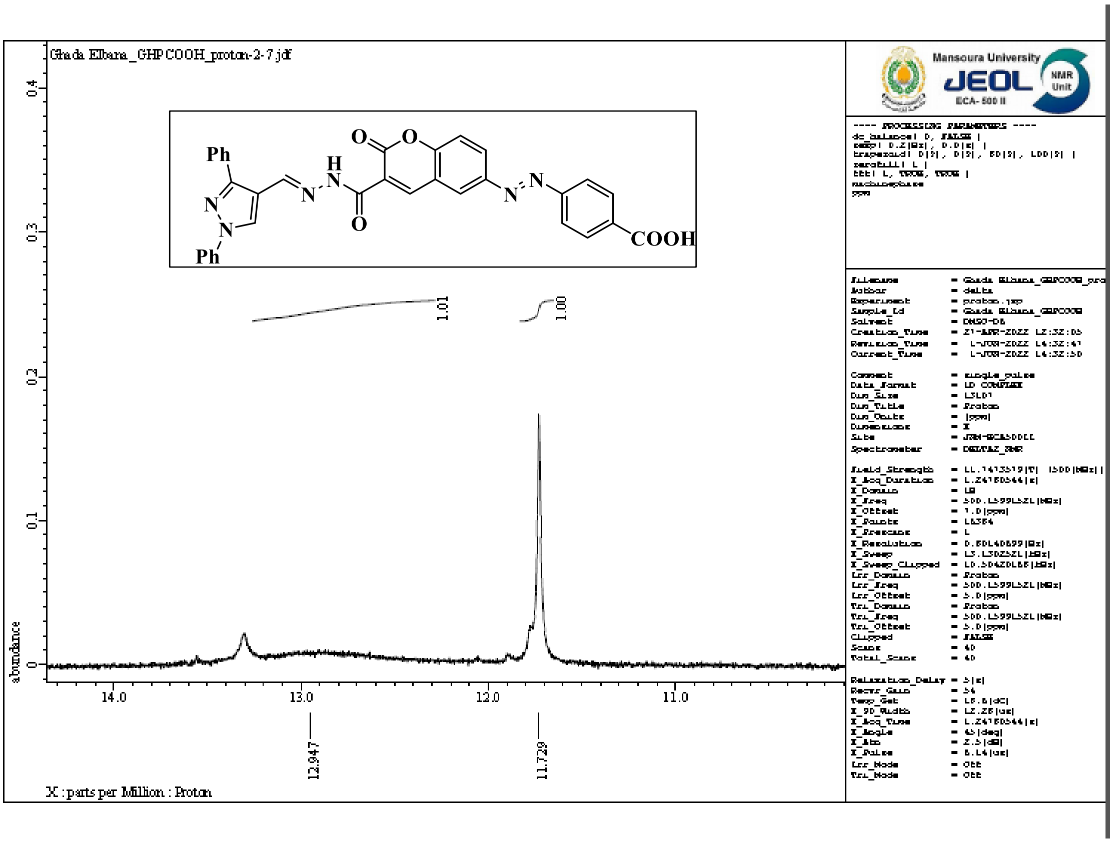

Figure S66: ^{1}H NMR spectrum of compound 10e

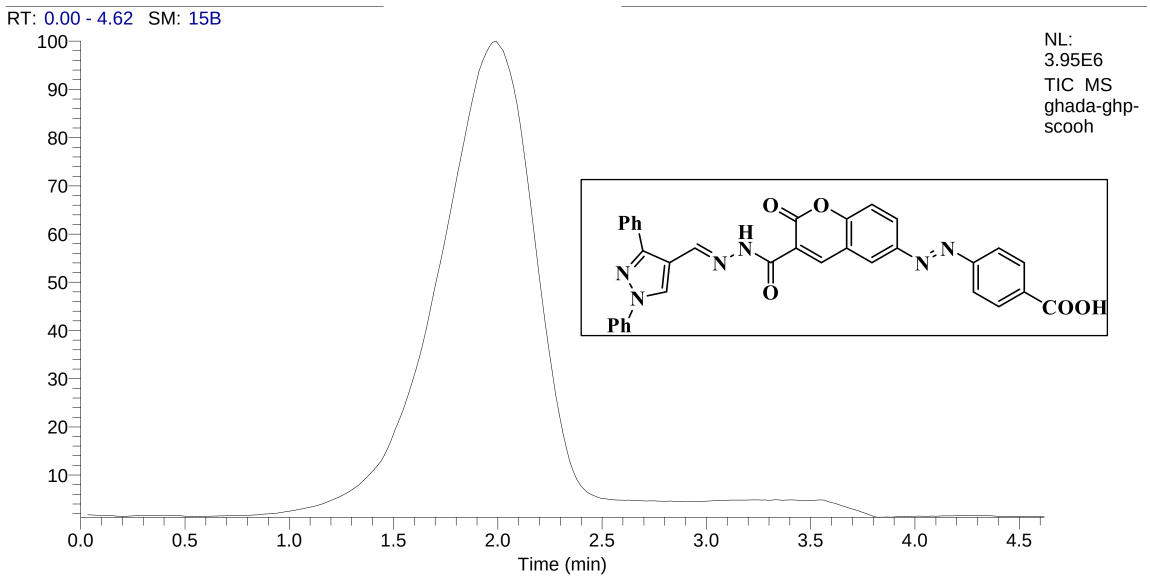

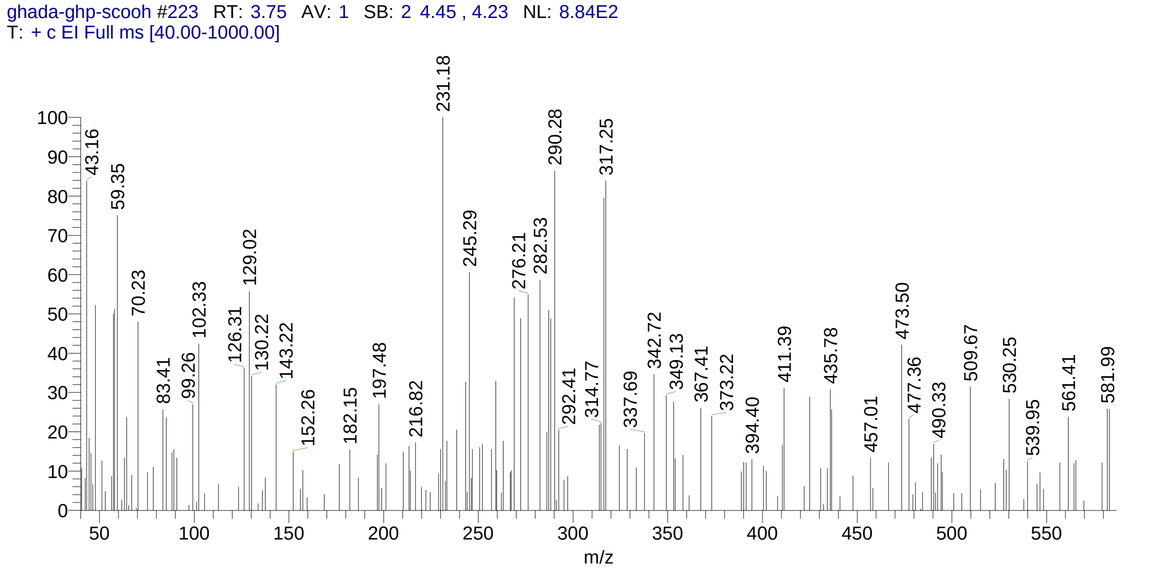

Figure S67: Mass spectrum of compound 10e

2-cyano-3-(dimethylamino)-*N'*-((1,3-diphenyl-1*H*-pyrazol-4-yl)methylene)-acrylo-hydrazide (11)

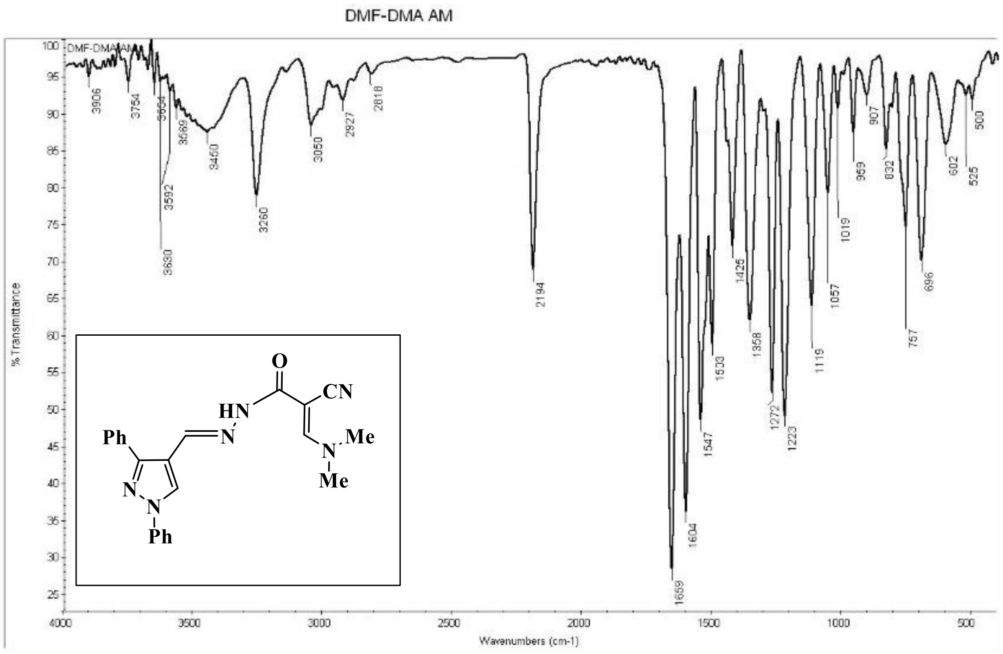

Figure S68: IR spectrum of compound 11

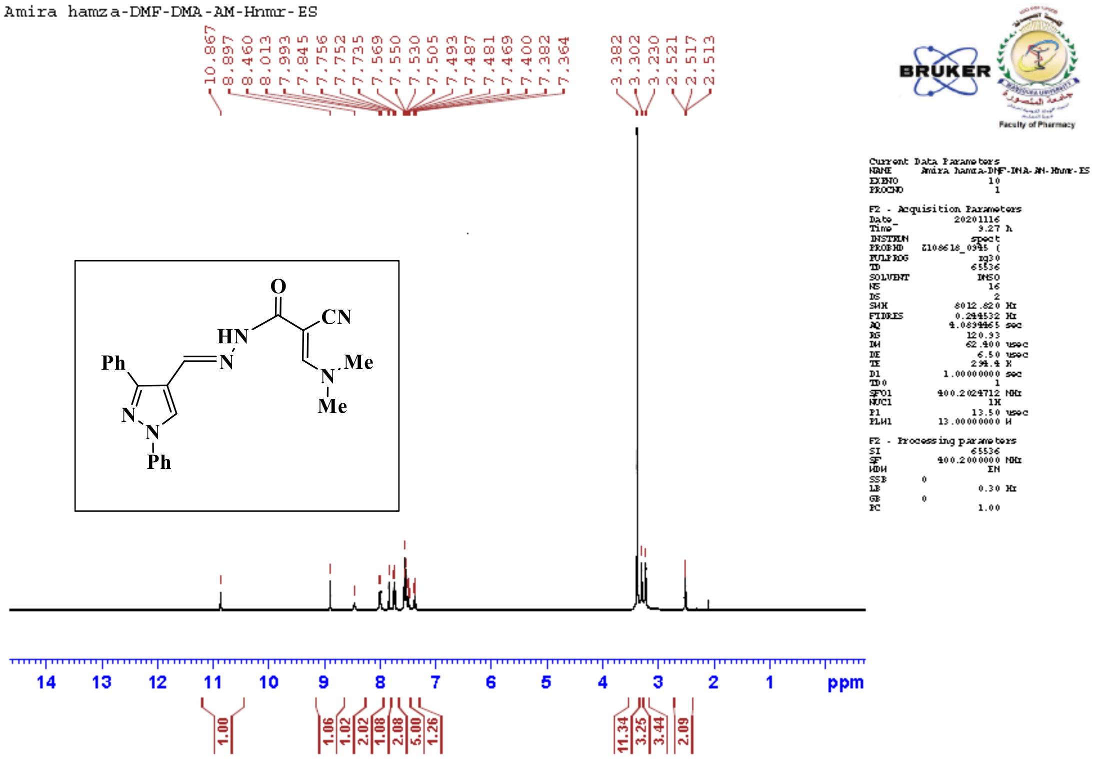

Figure S69: ¹H NMR spectrum of compound 11

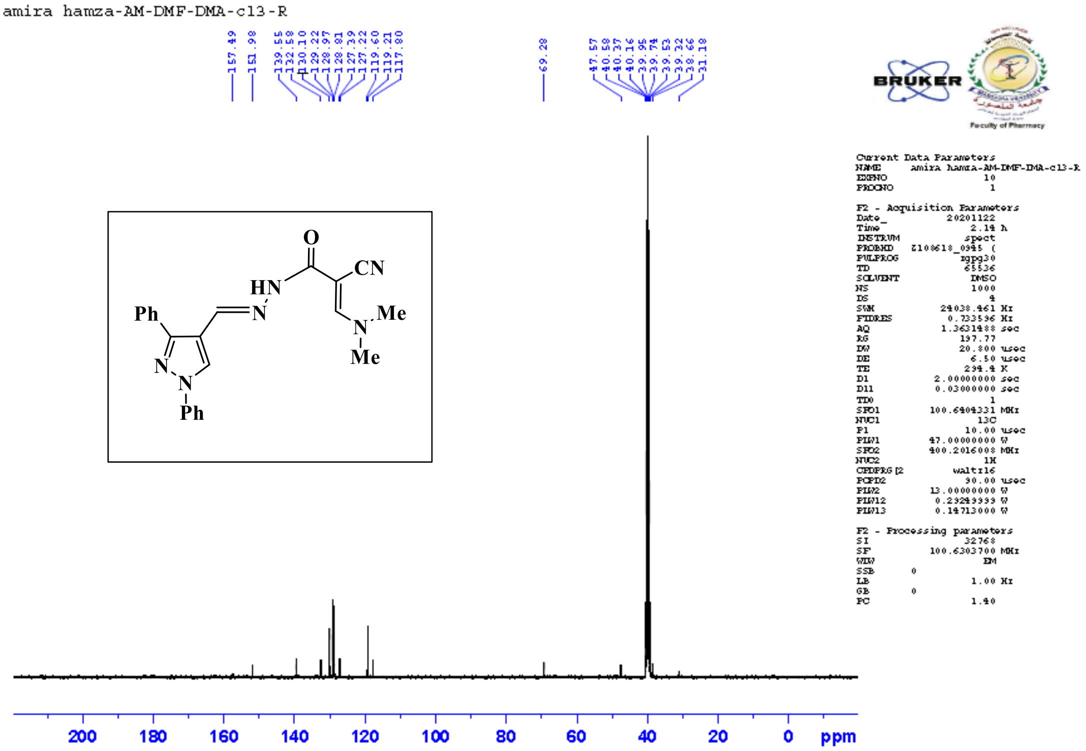

Figure S70S: ^{13}C NMR spectrum of compound 11

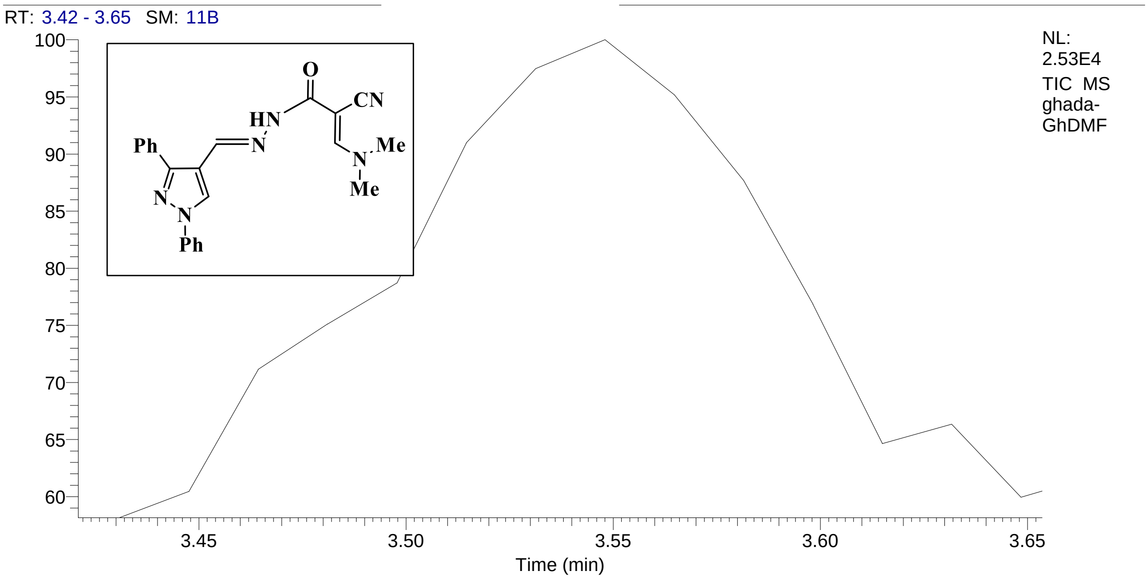

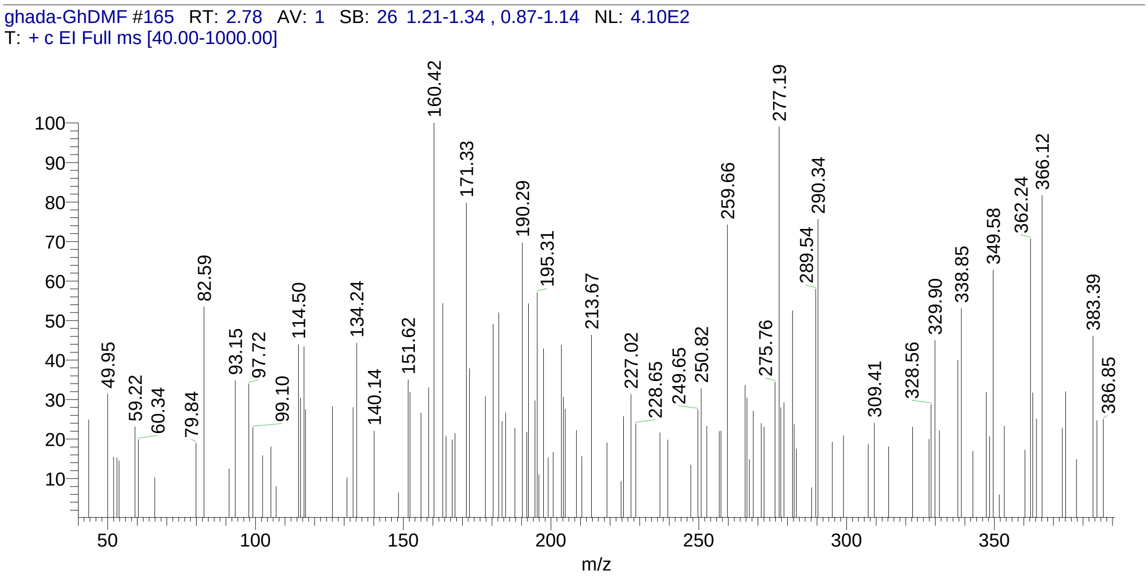

Figure S71: Mass spectrum of compound 11

4-((dimethylamino)methylene)-6-(1,3-diphenyl-1*H*-pyrazol-4-yl)pyridazine-3,5(4*H*,6*H*)-dione (12)

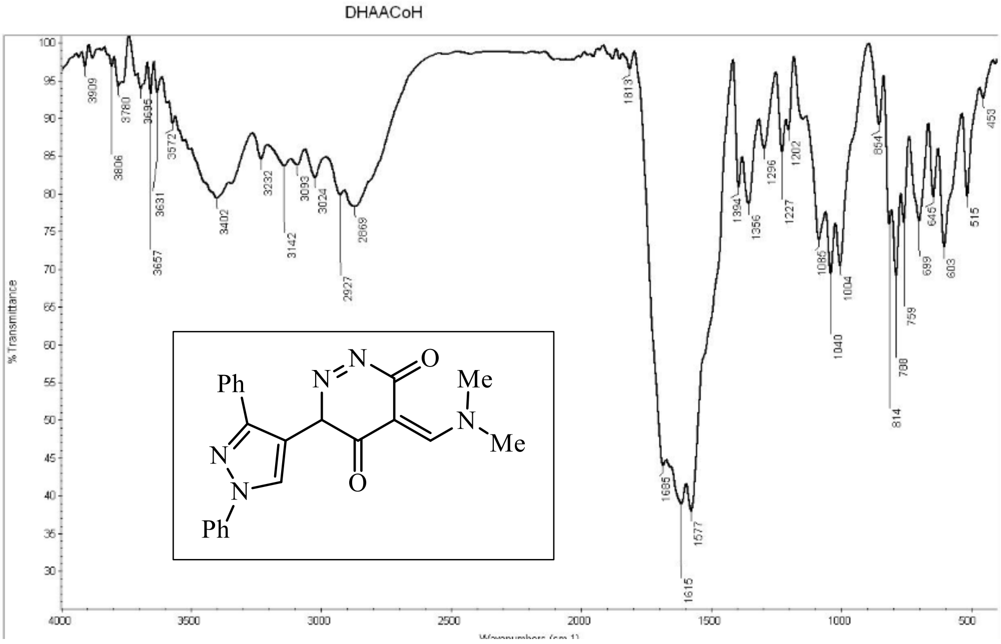

Figure S72: IR spectrum of compound 12

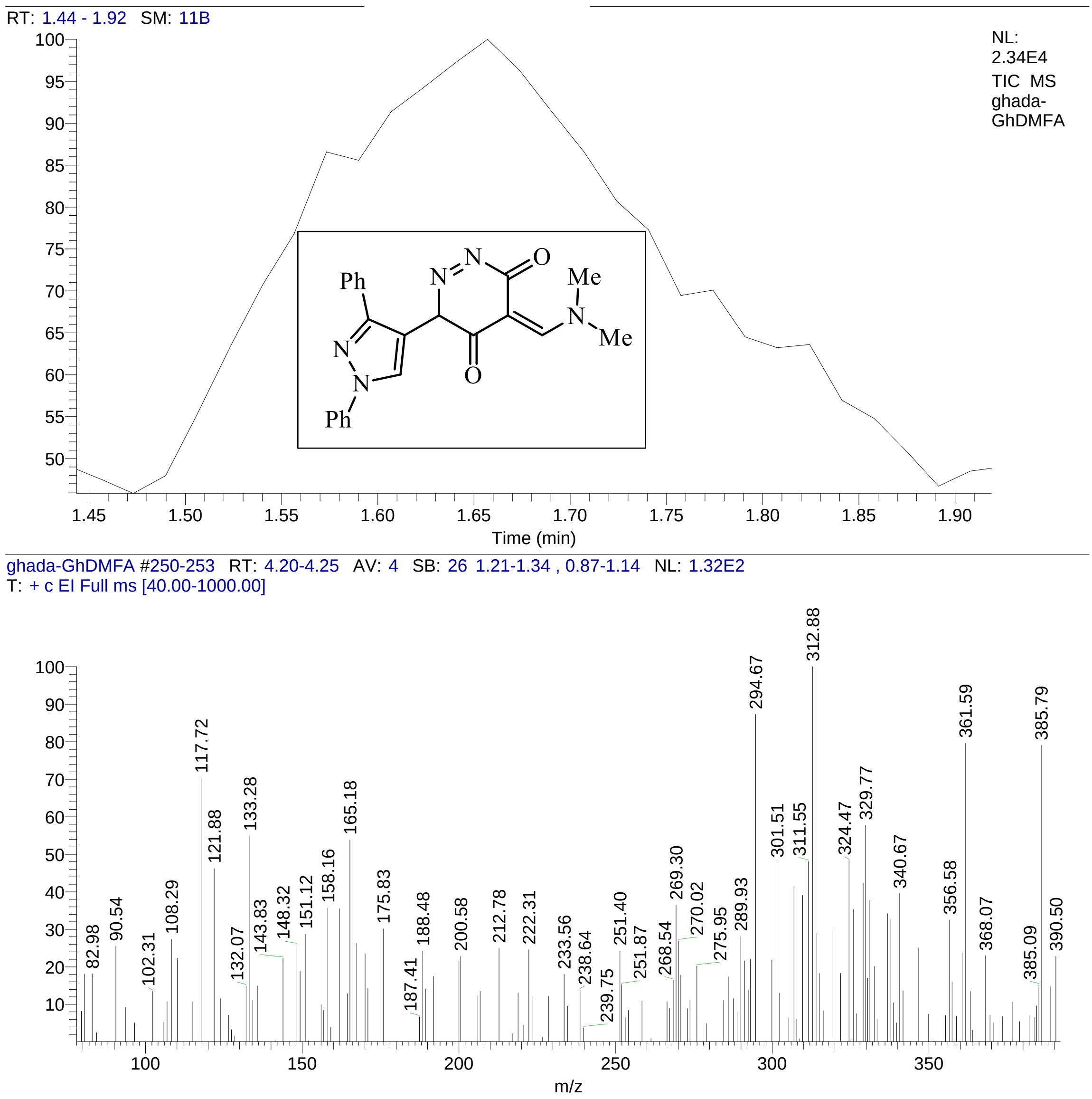

Figure S73: Mass spectrum of compound 12
